# CO-EXPRESSED WITH PSI ASSEMBLY1 (CEPA1) is a photosystem I assembly factor in Arabidopsis

**DOI:** 10.1093/plcell/koae042

**Published:** 2024-02-21

**Authors:** David Rolo, Omar Sandoval-Ibáñez, Wolfram Thiele, Mark A Schöttler, Ines Gerlach, Reimo Zoschke, Joram Schwartzmann, Etienne H Meyer, Ralph Bock

**Affiliations:** Department Organelle Biology, Biotechnology and Molecular Ecophysiology, Max Planck Institute of Molecular Plant Physiology, 14476 Potsdam-Golm, Germany; Department Organelle Biology, Biotechnology and Molecular Ecophysiology, Max Planck Institute of Molecular Plant Physiology, 14476 Potsdam-Golm, Germany; Department Organelle Biology, Biotechnology and Molecular Ecophysiology, Max Planck Institute of Molecular Plant Physiology, 14476 Potsdam-Golm, Germany; Department Organelle Biology, Biotechnology and Molecular Ecophysiology, Max Planck Institute of Molecular Plant Physiology, 14476 Potsdam-Golm, Germany; Department Organelle Biology, Biotechnology and Molecular Ecophysiology, Max Planck Institute of Molecular Plant Physiology, 14476 Potsdam-Golm, Germany; Department Organelle Biology, Biotechnology and Molecular Ecophysiology, Max Planck Institute of Molecular Plant Physiology, 14476 Potsdam-Golm, Germany; Department Organelle Biology, Biotechnology and Molecular Ecophysiology, Max Planck Institute of Molecular Plant Physiology, 14476 Potsdam-Golm, Germany; Department Organelle Biology, Biotechnology and Molecular Ecophysiology, Max Planck Institute of Molecular Plant Physiology, 14476 Potsdam-Golm, Germany; Department Organelle Biology, Biotechnology and Molecular Ecophysiology, Max Planck Institute of Molecular Plant Physiology, 14476 Potsdam-Golm, Germany

## Abstract

Photosystem I (PSI) forms a large macromolecular complex of ∼580 kDa that resides in the thylakoid membrane and mediates photosynthetic electron transfer. PSI is composed of 18 protein subunits and nearly 200 co-factors. The assembly of the complex in thylakoid membranes requires high spatial and temporal coordination, and is critically dependent on a sophisticated assembly machinery. Here, we report and characterize CO-EXPRESSED WITH PSI ASSEMBLY1 (CEPA1), a PSI assembly factor in Arabidopsis (*Arabidopsis thaliana*). The *CEPA1* gene was identified bioinformatically as being co-expressed with known PSI assembly factors. Disruption of the *CEPA1* gene leads to a pale phenotype and retarded plant development but does not entirely abolish photoautotrophy. Biophysical and biochemical analyses revealed that the phenotype is caused by a specific defect in PSI accumulation. We further show that CEPA1 acts at the post-translational level and co-localizes with PSI in nonappressed thylakoid membranes. In native gels, CEPA1 co-migrates with thylakoid protein complexes, including putative PSI assembly intermediates. Finally, protein–protein interaction assays suggest cooperation of CEPA1 with the PSI assembly factor PHOTOSYSTEM I ASSEMBLY3 (PSA3). Together, our data support an important but nonessential role of CEPA1 in PSI assembly.

## Teaser

Arabidopsis CO-EXPRESSED WITH PSI ASSEMBLY1 interacts with PHOTOSYSTEM I ASSEMBLY3 and plays an important but nonessential role in assembly of photosystem I.

**IN A NUTSHELL**

**Background:** Photosystem I (PSI) is a large pigment–protein complex that participates in photosynthetic electron transfer. While its structure is well resolved, its assembly pathway is less clear. A set of proteins mediating the step-wise assembly of PSI subunits, known as PSI assembly factors, has been previously characterized, but many key players are likely still missing. As an entry point into the discovery of yet-unknown PSI assembly factors, we screened for genes that are coexpressed with known PSI assembly factor-encoding genes in Arabidopsis. This approach led to the identification of COEXPRESSED WITH PSI ASSEMBLY1 (CEPA1).

**Questions:** What is the fate of PSI when CEPA1 is absent? How does CEPA1 function?

**Findings:** CEPA1 is targeted to the thylakoid membranes where PSI accumulates. Arabidopsis mutants lacking CEPA1 grow autotrophically but suffer from delayed development and exhibit pigment deficiency. This phenotype is caused by a strong decrease in PSI content. CEPA1 does not regulate plastid PSI gene expression, but instead acts at the posttranslational level. CEPA1 associates with PSI assembly intermediates in the thylakoid membrane and interacts with the PSI assembly factor PHOTOSYSTEM I ASSEMBLY3 (PSA3). The currently available data suggest a model in which CEPA1 is cleaved out of the nascent PSI complexes once its assembly function is fulfilled.

**Next steps:** The precise role of CEPA1 in PSI assembly will be further studied, in particular, by investigating the relationship between CEPA1 and PSA3 and by characterizing CEPA1-containing protein complexes.

## Introduction

Oxygenic photosynthesis converts light energy to chemical energy and is conserved from cyanobacteria to land plants (Stirbet et al. 2020). The photosynthetic apparatus, mainly composed of the thylakoid membrane-embedded complexes photosystem II (PSII), cytochrome *b*_6_*f* (Cyt*b*_6_*f*), photosystem I (PSI), and ATP synthase (ATPase), conducts the “light reactions” of photosynthesis (Johnson 2016). PSI mediates the electron transfer from the plastocyanin shuttle in the thylakoid lumen to ferredoxin in the stroma, which ultimately leads to the generation of reducing power in the form of NADPH that is then consumed by the Calvin–Benson–Bassham cycle (Bassham et al. 1950; Bengis and Nelson 1975; Caspy et al. 2020) and other anabolic reactions. In autotrophic eukaryotes, photosynthesis takes place in specialized organelles, the chloroplasts. The two photosystems are associated with their light-harvesting complex (LHC) to increase the cross-section of the antenna for light capture, thus ensuring efficient energy provision to drive photosynthetic electron transport.

In land plants, PSI–LHCI represents a large macromolecular complex of ∼580 kDa (Mazor et al. 2017; Caspy and Nelson 2018). The complex contains more than 200 ligands including 156 chlorophylls, 3 iron–sulfur (Fe_4_–S_4_) clusters, 2 phylloquinones, and a number of carotenoid and lipid molecules that all are necessary for efficient light harvesting and electron transport (Mazor et al. 2017). The ligands are attached to a set of 14 PSI core subunits and 4 LHCI subunits. As the result of plastid-to-nucleus gene transfer during evolution (Bock and Timmis 2008), some subunits are encoded in the plastid genome (i.e. the PSI subunits PsaA to PsaC, PsaI, and PsaJ), while the others are encoded in the nuclear genome (i.e. the PSI subunits PSAD-H, K, L, N, O, and the LHCI subunits LHCA1 to LHCA4) and post-translationally targeted to the chloroplast.

The intricate three-dimensional structure of PSI suggests that complex assembly in thylakoid membranes needs to be well coordinated in space and time. In contrast to the well-resolved structure of the complex, we still know relatively little about how PSI is assembled. PSI assembly is thought to be a fast process that mostly takes place in young developing leaf tissue. Once assembled, PSI is a very stable complex with a low turnover (Krech et al. 2012; Nelson et al. 2014; Schöttler and Tóth 2014; Schöttler et al. 2015; Li et al. 2018). For these reasons, the isolation of intermediates in PSI biogenesis that represent distinct steps of the PSI assembly process has proven to be challenging. A preliminary model of the assembly pathway was derived from the PSI structure, the functional characterization of PSI mutants, and the isolation of two putative PSI assembly intermediates in tobacco (*Nicotiana tabacum*) and Chlamydomonas (*Chlamydomonas reinhardtii*; Jensen et al. 2007; Ozawa et al. 2010; Schöttler et al. 2011; Wittenberg et al. 2017). In this model, the PSI assembly process starts with the co-translational insertion of the reaction center composed of the PsaA–PsaB heterodimer into the thylakoid membrane (Göhre et al. 2006). Then, the stromal ridge involved in the docking of ferredoxin and composed of the subunits PsaC, PSAD, and PSAE is incorporated, followed by addition of the small subunits PsaI, PSAH, and PSAL (and possibly PSAO), to form the stable intermediate PSI* (Wittenberg et al. 2017). Finally, the insertion of PSAF leads to maturation of the complex by addition of the last core subunits and the LHCI complex. Although the precise order in which those final subunits are added is not known, the PSI structure suggests that PsaJ may be added before LHCI (Mazor et al. 2017). LHCI may then associate loosely to the PSI core until it becomes buckled by PSAG and PSAK on either side of the core (Ozawa et al. 2010). PSAN associates near the lumenal loop of PSAF (after PSAF integration into the complex), however, it is currently unclear whether this occurs before or after insertion of the final subunits of PSI–LHCI.

The integration of the different subunits at the right time and in the right place is critically dependent on a finely tuned PSI assembly machinery. In the past three decades, seven proteins have been characterized as being specifically involved in PSI assembly, and they are collectively referred to as PSI assembly factors. They comprise the two plastid-encoded hypothetical chloroplast reading frames number 3 (Ycf3; Boudreau et al. 1997; Ruf et al. 1997; Naver et al. 2001) and number 4 (Ycf4; Boudreau et al. 1997; Krech et al. 2012), and the five nucleus-encoded factors PALE YELLOW GREEN 7 (PYG7; Wilde et al. 2001; Stöckel et al. 2006; Heinnickel et al. 2016; Yang et al. 2017), Ycf3-INTERACTING PROTEIN 1 (Y3IP1; Albus et al. 2010), PSBP-DOMAIN PROTEIN 1 (PPD1; Liu et al. 2012; Roose et al. 2014), and PHOTOSYSTEM I ASSEMBLY 2 (PSA2; Fristedt et al. 2014; Wang et al. 2016) and 3 (PSA3; Shen et al. 2017). These assembly factors interact with PSI subunits and/or each other to mediate proper incorporation of the subunits into the nascent complex. Notably, the Ycf3–Y3IP1 module and the Ycf4 oligomeric scaffold are important for the early steps of PSI reaction center assembly, with PPD1 participating from the lumenal side, while PYG7 and PSA3 cooperate in the insertion of PsaC (Schöttler et al. 2011; Liu et al. 2012; Yang et al. 2015; Nellaepalli et al. 2018, 2021). At least some of these factors are likely also involved in later stages of the assembly process, as suggested by their association with PSI subcomplexes that contain subsequently assembled PSI subunits (Stöckel et al. 2006; Ozawa et al. 2009; Krech et al. 2012; Shen et al. 2017).

The complete elucidation of the composition of the PSI assembly machinery is indubitably an arduous but fundamental task on the long road toward the full description of the PSI assembly process. In this work, we have pursued a new approach to search for unknown PSI assembly factors. We reasoned that PSI assembly requires the proper coordination of the assembly factors involved during chloroplast biogenesis, and searched for genes that are co-expressed with the five previously identified nucleus-encoded PSI assembly factors in the model plant Arabidopsis (*Arabidopsis thaliana*) using the EnsembleNet tool (Hansen et al. 2018). The resulting list of co-expressed genes was then reduced to genes coding for plastid-localized proteins of uncharacterized function, and the corresponding mutants were screened for PSI defects. This approach led to the identification of CO-EXPRESSED WITH PHOTOSYSTEM I ASSEMBLY 1 (CEPA1). We report the functional characterization of CEPA1 and show that it is a PSI assembly factor. We show that disruption of *CEPA1* leads to a specific decrease in PSI accumulation, but is not lethal under autotrophic growth conditions. Protein–protein interaction assays indicate that CEPA1 cooperates with PSA3 in PSI assembly. Overall, our data suggest that CEPA1 acts post-translationally and associates with specific PSI assembly intermediates before it dissociates from the complex upon maturation of PSI–LHCI.

## Results

### Identification of CEPA1 as a candidate PSI assembly factor

To identify factors involved in PSI biogenesis, we looked for genes that are co-expressed with PSI assembly factors. To obtain a list of candidate genes, we used the “Gene set search” tool in the EnsembleNet suite, which predicts genes that may be functionally related to a given gene set, largely based on integration of co-expression networks in Arabidopsis (Hansen et al. 2018). Our query gene set consisted of the five known nuclear genes that code for PSI assembly factors: *PYG7*, *Y3IP1*, *PPD1*, *PSA2*, and *PSA3*.

The original EnsembleNet output was a list of 880 genes connected to at least two of the query genes, among which 865 were nucleus-encoded genes. Two hundred and seventy-eight genes were connected to all five query genes, and 262 of them code for proteins that are targeted to the plastid (based on published data and/or SUBA4 prediction; Hooper et al. 2017), where the photosynthetic machinery is located. The list was then narrowed down to 29 genes that were uncharacterized in that they had no annotation, or only predicted functions and/or features that could be compatible with a role in PSI assembly (e.g. presence of a transmembrane domain, or a putative protein–protein interaction domain) according to the TAIR database (Reiser et al. 2022). The final candidate gene list contained 22 genes that we preliminarily named “Co-Expressed with PSI Assembly (CEPA)” genes (Supplementary Table S1). As part of the CEPA short-list, the At3g56010 gene was named *CO-EXPRESSED WITH PSI ASSEMBLY 1* (*CEPA1*; Table 1).

**Table 1.** *CEPA1* is co-expressed with all five previously identified nuclear genes encoding PSI assembly factors

GOJI	AGI locus	Alias	References
0.2398	At1g22700	*PYG7*	Stöckel et al. (2006), Yang et al. (2017)
0.2923	At5g44650	*Y3IP1*	Albus et al. (2010)
0.1875	At4g15510	*PPD1*	Liu et al. (2012), Roose et al. (2014)
0.3505	At2g34860	*PSA2*	Fristedt et al. (2014), Wang et al. (2016)
0.2789	At3g55250	*PSA3*	Shen et al. (2017)

The EnsembleNet “Gene set search” output was based on a query of all five known nuclear-encoded PSI assembly factors (Hansen et al. 2018). The Gene Ontology Jaccard Index (GOJI) scores estimating the relation between *CEPA1* and the nuclear genes coding for PSI assembly factors were obtained by searching the *CEPA1* gene in the EnsembleNet “Gene search” option. A GOJI > 0.178 means that the genes are potentially functionally related (Hansen et al. 2018). The studies in which the PSI assembly factors were characterized in land plants are indicated.

**Figure 1.**
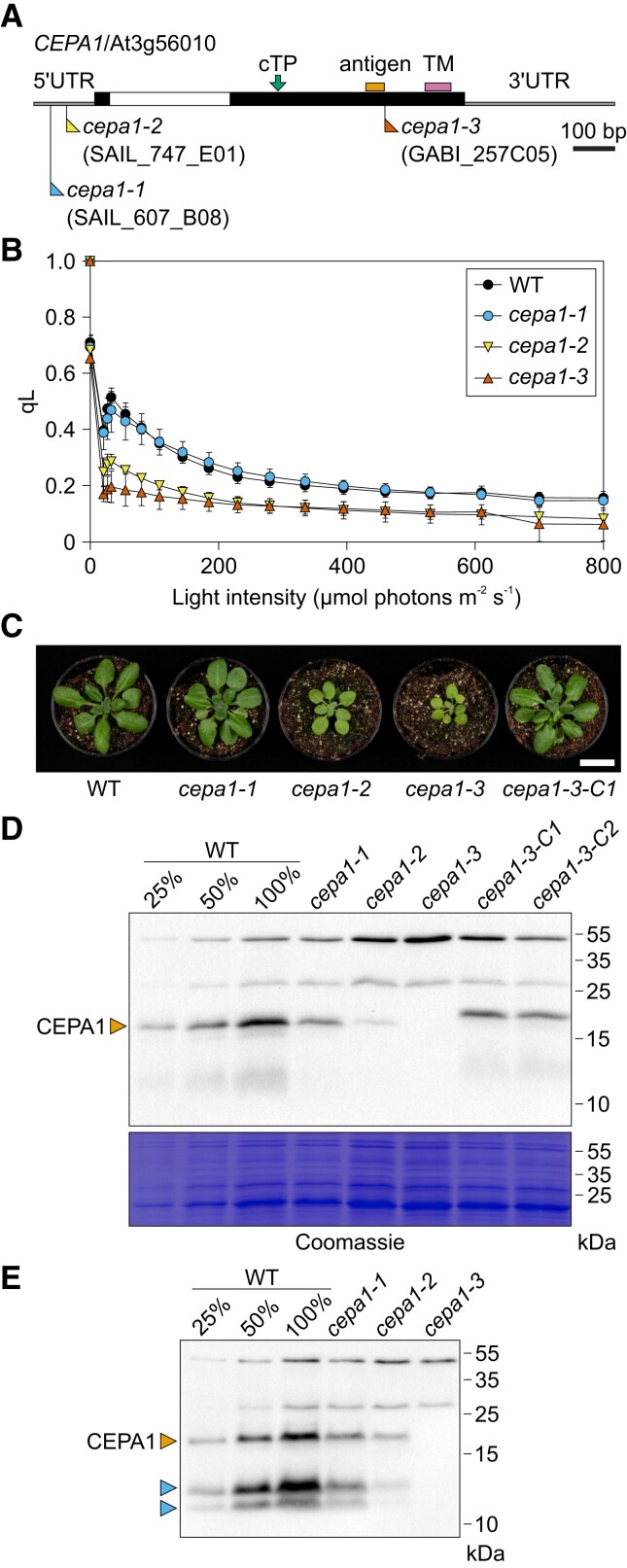
Identification of *CEPA1* as a candidate PSI-related gene through qL screening of T-DNA insertion lines. **A)** Physical map of the *CEPA1* gene with relevant features indicated. The blue, yellow, and red flags indicate the T-DNA insertion sites in *cepa1-1*, *cepa1-2*, and *cepa1-3*, respectively. The filled and open boxes represent the exons and the intron, respectively. The green arrow shows the cTP cleavage site. The orange box marks the region coding for the antigenic peptide (antigen) used for raising anti-CEPA1 antibodies, and the pink box indicates the predicted transmembrane (TM) domain. Gene sequence is reported in Supplementary Table S2. Scale bar: 100 bp. **B)** Light response curve of the redox state of the PSII acceptor side (qL) in the *cepa1-1*, *cepa1-2*, and *cepa1-3* mutants, and the wild type (WT). Measurements were conducted on five seedlings per line, grown for 10 d on 0.5 MS + 1% (w/v) sucrose in long-day regime. Error bars represent the standard deviation (Sd). **C)** Development of the WT, the three T-DNA insertion mutants and the complemented line *cepa1-3-C1*. The image was taken after 4 wk of growth on soil in long-day conditions. Scale bar: 2 cm. **D)** Immunoblot to assess CEPA1 expression in the WT, the T-DNA insertion mutants and two complemented lines. Antibodies recognizing the CEPA1 protein were raised in rabbits against a CEPA1-specific peptide. Proteins from isolated thylakoid membranes of 3-wk-old plants (equal to 1 *µ*g chlorophyll) were separated by SDS-PAGE, and subjected to immunoblotting with antibodies against CEPA1. To assess protein accumulation in the mutants, a dilution series of the WT sample (25%, 50%, and 100%) was loaded. The membrane used for immunoblotting was stained with Coomassie as a loading control. The orange arrowhead indicates the signal corresponding to the mature CEPA1. **E)** Accumulation of CEPA1 in thylakoid membranes of 5-wk-old plants with fully developed rosettes by immunoblotting. The orange and two blue arrowheads indicate the signal corresponding to the mature CEPA1 and putative CEPA1 fragments, respectively.

Consistent with *CEPA1* being co-expressed with PSI assembly factors, RNA-seq datasets show a higher expression of *CEPA1* in early developing leaves in Arabidopsis, where chloroplast biogenesis mostly takes place (Klepikova et al. 2016; Supplementary Fig. S1A). The *CEPA1* gene encodes a putative precursor protein of 21.9 kDa, which is predicted to give rise to a mature protein of 16.3 kDa after import into the chloroplast and cleavage of the putative chloroplast transit peptide (cTP) at position C51-L52 according to TargetP-2.0 (Almagro Armenteros et al. 2019; Fig. 1A, Supplementary Fig. S1, B and C and Supplementary Table S2). In agreement with this prediction, a semi-tryptic peptide starting at amino acid position L52 is reported in the PPDB database (Sun et al. 2009). Moreover, in the Arabidopsis PeptideAtlas (van Wijk et al. 2021), the same peptide, annotated as PAp06471115, is the most abundant peptide in the proteomic datasets, and therefore, likely corresponds to the tryptic peptide derived from the N-terminus of the mature CEPA1 after cTP cleavage (Supplementary Fig. S1B). CEPA1 has a single transmembrane domain toward its C-terminal end, predicted by DeepTMHMM (Hallgren et al. 2022; Fig. 1A, Supplementary Fig. S1, B and C and Supplementary Table S2), but no conserved functional domain has been assigned to CEPA1 sequence and structure (Supplementary Fig. S1C). Finally, CEPA1 peptides were previously identified in proteomic datasets from chloroplasts and thylakoid membrane fractions (Peltier et al. 2004; Tomizioli et al. 2014), in line with the presence of a cTP and a membrane-spanning segment. Thus, the CEPA1 features are consistent with a potential role in thylakoid membranes, where PSI is embedded.

To preliminarily screen the CEPA candidate genes for PSI assembly factors, at least one T-DNA insertion line per gene was selected and homozygous mutant lines were isolated (Supplementary Table S1). For the *CEPA1* gene, three T-DNA insertion lines were obtained: *cepa1-1* (SAIL_607_B08), *cepa1-2* (SAIL_747_E01), and *cepa1-3* (GABI_257C05), and their genotypes were confirmed by PCR amplification of the T-DNA insertion site in the *CEPA1* locus and amplicon sequencing of the borders of the insertion (Fig. 1A, Supplementary Fig. S2 and Supplementary Table S2).

All homozygous T-DNA insertion lines of the CEPA short list were then preliminarily screened for PSI-deficient mutants based on their qL light response curves (LRC) by chlorophyll-*a* fluorescence imaging measurements, referred to as qL screening. The qL parameter, a measure for the redox state of the PSII acceptor side (Kramer et al. 2004), is expected to decrease strongly upon reduced PSI accumulation, due to overexcitation of PSII and rapid reduction of the PSII acceptor side and the plastoquinone pool already under light-limited conditions (Albus et al. 2010). In the case of the *CEPA1* gene, the *cepa1-2* and *cepa1-3* mutants exhibited substantially lower qL values than the wild type (WT), especially at low light intensity (Fig. 1B). By contrast, no differences in the qL parameter were observed between the *cepa1-1* mutant and the WT. As no mutants for any of the other co-expressed genes displayed the qL LRC typical of mutants with low PSI accumulation, the *CEPA1* gene was selected for further analysis.

Given the CEPA1 features and the phenotype of the *cepa1-2* and *cepa1-3* mutants in the qL screening (Fig. 1, Supplementary Fig. S1), we considered CEPA1 as a promising candidate PSI assembly factor, and decided to further investigate its function in Arabidopsis. We first assessed the impact of *CEPA1* disruption on plant development. While the *cepa1-2* and *cepa1-3* mutants survive in soil, they develop more slowly and have paler leaves than the WT, in line with a defect in photosynthesis (Fig. 1C). By contrast, *cepa1-1* is phenotypically indistinguishable from the WT.

Next, antibodies were raised against a CEPA1 peptide (LLDVLKESDKKSKRK) for analysis of CEPA1 accumulation in thylakoid samples from 3-wk-old WT and *cepa1* mutants by immunoblot assays (Fig. 1, D and E). Two cross-reacting proteins of higher molecular weight were visible in all lines (∼50 and ∼30 kDa) and served as convenient loading control. A specific signal between 15 and 25 kDa corresponding in size to CEPA1 is detected in the WT, but not in the *cepa1-3* mutant (Fig. 1D). CEPA1 protein amounts are around half of the WT level in the *cepa1-1* mutant and less than 25% of the WT level in the *cepa1-2* mutant. These findings suggest that *cepa1-3* represents a knock-out allele, while *cepa1-1* and *cepa1-2* are knock-down mutants. CEPA1 accumulation levels in the mutants can be explained by the different T-DNA insertion sites. The T-DNA resides upstream in the 5′UTR of *CEPA1* in the *cepa1-1* mutant, closer to the coding region in the *cepa1-2* mutant, and in the second exon in the *cepa1-3* mutant (Fig. 1A). The severity of the mutant phenotype observed for *cepa1-2* and *cepa1-3* correlates with the residual CEPA1 accumulation levels in these two mutants. Notably, the 50% reduction in CEPA1 accumulation in the *cepa1-1* mutant does not affect the phenotype under standard growth conditions (Fig. 1, C and D). Two additional signals at lower molecular weight than the mature CEPA1 protein (between 10 and 15 kDa) are detected in the immunoblots. These smaller signals are more prominent in 5-wk-old plants (blue arrows in Fig. 1E) and follow the same accumulation trend as the mature CEPA1. Thus, they are likely attributable to CEPA1 fragments (e.g. degradation products) that still contain the antigenic sequence recognized by the antibodies.

To confirm that the phenotype observed in the mutants is specifically caused by the disruption of *CEPA1* expression, a *P_UBQ_:CEPA1-FLAG* cassette (in vector pDR016; Supplementary Fig. S3A) was constructed and introduced into the *cepa1-3* mutant by *Agrobacterium*-mediated floral-dip transformation. Two independent complemented lines, named *cepa1-3-C1* and *cepa1-3-C2*, were selected on medium supplemented with phosphinothricin (to which resistance is conferred by the *pat* resistance gene in the transformation vector; Supplementary Fig. S3B), and cassette insertion into the genome was confirmed by PCR genotyping (Supplementary Fig. S3C). The expression of the CEPA1-FLAG protein in the complemented lines was confirmed by immunoblot analysis (Fig. 1D). The size increase of the recombinant protein in comparison to the native CEPA1 (due to the presence of the FLAG tag) can be readily visualized. The expression of CEPA1-FLAG in the *cepa1-3* background is sufficient to restore WT-like growth and development (Fig. 1C). These data demonstrate that *CEPA1* disruption is specifically responsible for the phenotype of the mutants.

### CEPA1 is conserved in photosynthetic eukaryotes

Even though PSI is relatively well conserved throughout evolution, some structural adaptations have occurred, especially the shift from multimeric to monomeric PSI forms and the appearance of membrane-intrinsic light-harvesting antenna proteins in photosynthetic eukaryotes (Amunts and Nelson 2008, 2009; Minagawa and Tokutsu 2015). Consequently, PSI assembly has co-evolved with PSI structure. For instance, Y3IP1, PPD1, PSA2, and PSA3 are present in photosynthetic eukaryotes, but not in prokaryotes. Thus, we were interested in assessing the evolutionary conservation of CEPA1.

**Figure 2.**
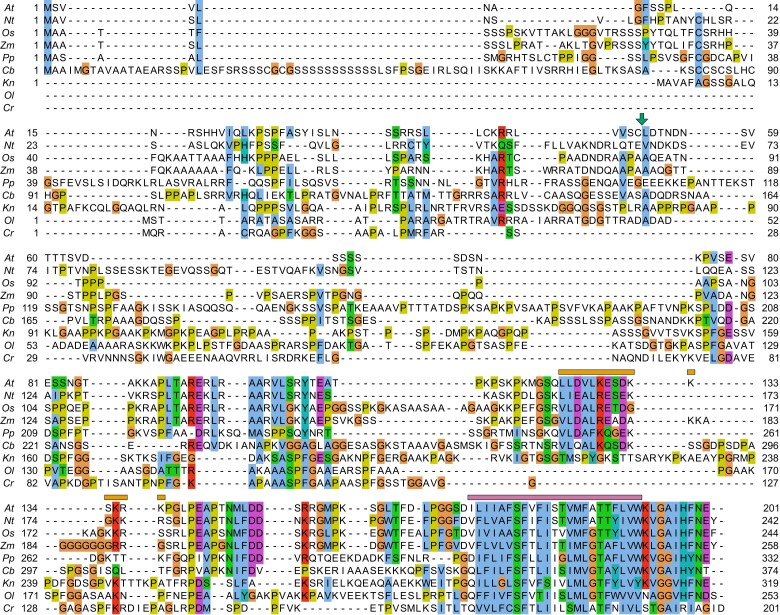
Conservation of CEPA1 in photosynthetic eukaryotes. Putative homologs of CEPA1 were selected from BLASTp searches with the AtCEPA1, CbCEPA1, and OlCEPA1 protein sequences as queries. The alignment of CEPA1 homologous sequences from *A. thaliana* (*At*), *N. tabacum* (*Nt*), *O. sativa* (*Os*), *Z. mays* (*Zm*), *P. patens* (*Pp*), *C. braunii* (*Cb*), *K. nitens* (*Kn*), *O. lucimarinus* (*Ol*), and Chlamydomonas (*C. reinhardtii*; *Cr*) was performed using Clustal Omega online (Sievers et al. 2011). The output alignment was edited with Jalview (version 2.11.1; Waterhouse et al. 2009). Conserved amino acids are highlighted according to the ClustalX color scheme (Thompson et al. 1997): hydrophobic (i.e. A, F, I, L, M, V, and W; in blue), positively charged (i.e. K and R; in red), negatively charged (i.e. D and E; in purple), polar (i.e. N, Q, S, and T; in green), aromatic (i.e. H and Y; in turquoise), glycine (G; in orange) and proline (P; in yellow). The green arrow, orange box, and pink box indicate the predicted cTP cleavage site in AtCEPA1, the antigen selected for AtCEPA1-specific antibody production (LLDVLKESDKKSKRK), and the transmembrane domain in AtCEPA1 (ILIIAFSFVFISTVMFATTFLVW), respectively. Note the sequence divergence between Chlamydomonas and land plants. However, the C-terminal part including the transmembrane domain is relatively well conserved among all organisms presented here.

To identify putative orthologs of CEPA1, the CEPA1 protein sequence from Arabidopsis (AtCEPA1) was used to conduct BLASTp searches (Boratyn et al. 2013). The Arabidopsis sequence was aligned with sequence hits in BLASTp from tobacco, rice (*Oryza sativa*), maize (*Zea mays*), and moss (*Physcomitrium patens*) (Fig. 2). The C-terminal region of CEPA1 that contains the predicted transmembrane domain is relatively well conserved in all these organisms (Fig. 2). When using the AtCEPA1 sequence as a query in BLASTp, no homolog was found in photosynthetic prokaryotes and in the green alga Chlamydomonas, a model organism for unicellular photosynthetic eukaryotes. However, we obtained a hit in *Chara braunii*, a Charophyte that is part of the lineage considered to be closest to land plants (Fig. 2; Nishiyama et al. 2018).

Considering that putative homologs in the distantly related organisms cyanobacteria and Chlamydomonas might be too divergent to give a direct hit with the AtCEPA1 sequence in BLASTp, the *C. braunii* CEPA1 (CbCEPA1) sequence was then entered as a query in BLASTp. When including only land plants in the search database, the original AtCEPA1 was present in the hit list as well as all the other putative homologs exemplarily included in Fig. 2. When excluding land plants, no hit was found in cyanobacteria and Chlamydomonas. However, results contained a hit in several green algae such as the multicellular *Klebsormidium nitens*, a member of the Klebsormidiophyceae, and interestingly, in the unicellular alga *Ostreococcus lucimarinus*, a member of the Chlorophyta like Chlamydomonas (Fig. 2). Thus, the sequence of the putative *O. lucimarinus* CEPA1 homolog (OlCEPA1) was used as a final query in BLASTp. There was no hit in the land plant and cyanobacteria databases. However, there was a protein hit in Chlamydomonas (XP_001689895.2; Fig. 2). This protein is associated to the locus Cre01.g014000 in the Chlamydomonas genome (Merchant et al. 2007). Even though it was not identified as a direct hit with the AtCEPA1 sequence in BLASTp, this protein could represent the Chlamydomonas CEPA1 (CrCEPA1) homolog or at least a related ancestor. In line with this assumption, CrCEPA1 is considered to be the homolog of AtCEPA1 in the Chlamydomonas proteome with a probability of 99.89% according to HHpred analysis (Zimmermann et al. 2018). Moreover, screens of the mutant collection from the Chlamydomonas library project (CLiP) revealed poor or arrested growth in autotrophic medium of mutants whose Cre01.g014000 gene was disrupted (with the exception of mutants in which only the 3′UTR region was affected; Supplementary Fig. S4 and Supplementary Table S3; Li et al. 2016, 2019; Fauser et al. 2022). For this reason, the Cre01.g014000 gene had been annotated as *LIGHT GROWTH SENSITIVE 1* (*LGS1*; Fauser et al. 2022).

In conclusion, CEPA1 is conserved among photosynthetic eukaryotes, from green algae to land plants, but likely absent from prokaryotes.

### The *cepa1* mutants are specifically deficient in PSI accumulation

**Figure 3.**
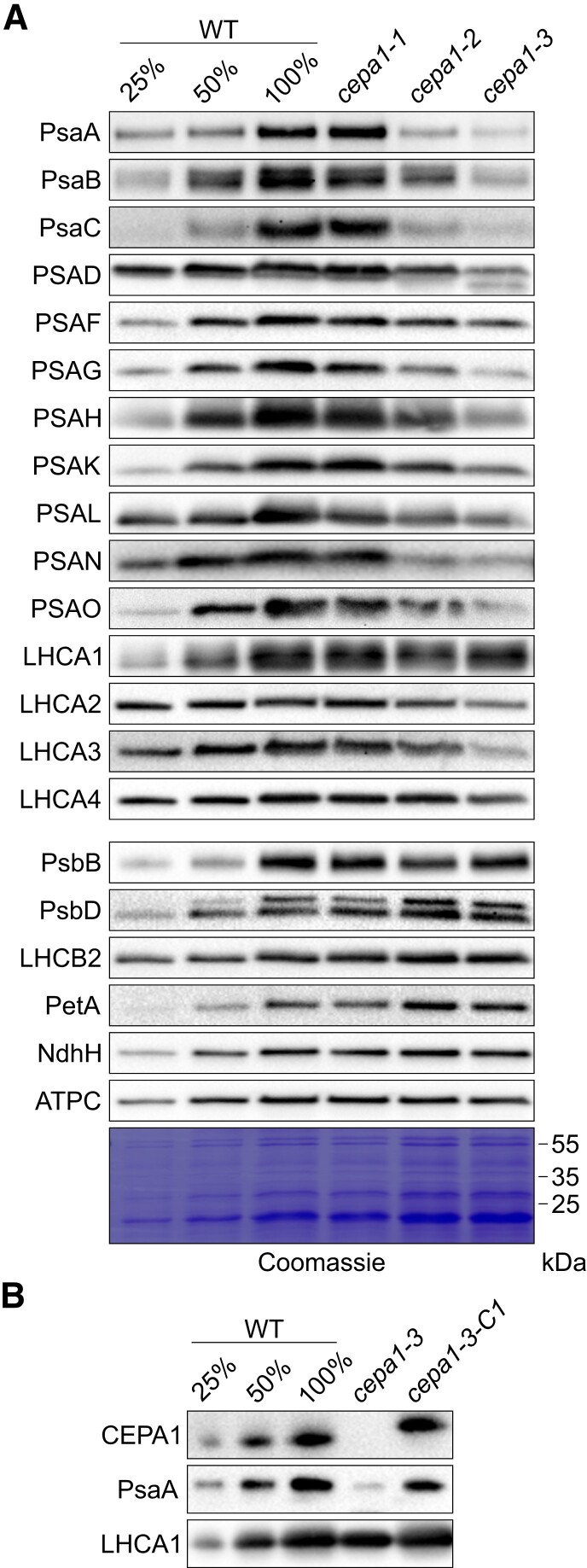
The *cepa1-2* and *cepa1-3* mutants are specifically deficient in the accumulation of PSI subunits in thylakoid membranes. **A)** Immunoblot analysis of selected subunits of thylakoid protein complexes. Thylakoid membrane proteins isolated from the WT and T-DNA insertion lines (equal to 1 *µ*g chlorophyll) were separated by SDS-PAGE and subjected to immunoblotting using antibodies against CEPA1 and diagnostic thylakoid complex subunits. Antibodies against all PSI core subunits (except PSAE, PsaI, and PsaJ, due to unavailable antibodies) and LHCI subunits were used, as well as antibodies against PsbB and PsbD (PSII core), LHCB2 (LHCII), PetA (Cyt*b*_6_*f*), NdhH (NDH), and ATPC (ATPase). Note that all PSI subunits accumulate less in *cepa1-2* and *cepa1-3*, while the other complexes are largely unaffected. One blotted membrane was stained with Coomassie as a loading control. Because samples were loaded based on equal chlorophyll amounts, the *cepa1-2* and *cepa1-3* (that accumulate less chlorophyll) are slightly overloaded in comparison to the WT. **B)** Immunoblots to assess restoration of CEPA1 and PSI–LHCI accumulation in a complemented line. Thylakoid membrane proteins isolated from the WT, *cepa1-3* and *cepa1-3-C1* (equal to 1 *µ*g chlorophyll) were separated by SDS-PAGE and subjected to immunoblotting using antibodies against CEPA1, PSI (PsaA), and LHCI (LHCA1). WT-like accumulation of CEPA1 and PSI–LHCI is fully restored in the *cepa1-3-C1* complemented line. The FLAG tag of the CEPA1-FLAG protein accumulating in *cepa1-3-C1* explains the size shift detected with antibodies against CEPA1. To assess protein accumulation in the mutants, a dilution series of the WT sample (25%, 50%, and 100%) was loaded.

To investigate why *CEPA1* disruption disturbs plant development, we wanted to examine whether the acceptor side limitation of PSII in *cepa1* mutants is due to inefficient accumulation of downstream complexes. To this end, we used thylakoid protein samples for immunoblotting with antibodies against diagnostic complex subunits. These analyses revealed that the amounts of all PSI subunits are decreased in *cepa1-2* and more strongly in *cepa1-3* compared to in the WT (Fig. 3A). While all core subunits are decreased to similar extents in the mutants, LHCI subunit accumulation seems to be less affected. No significant deficiency in any other thylakoid complex subunits is observed in *cepa1-2* and *cepa1-3*. Consistent with its phenotype, *cepa1-1* accumulates all complex subunits to WT-like levels. In the complemented line *cepa1-3-C1*, WT-like levels of the PSI reaction center protein PsaA, essential for PSI accumulation, are restored (Fig. 3B). The weak band of lower molecular weight detected in *cepa1-3* with antibodies against PSAD likely represents a degradation intermediate of PSAD, and is not always observed. For instance, young *cepa1-3* seedlings grown under aseptic conditions on plates (i.e. in more controlled conditions) do not accumulate this product (Supplementary Fig. S5A). Additionally, when photosynthetic complex assembly is arrested by treatment with lincomycin, an inhibitor of plastid gene expression, PSI accumulation remains relatively stable (as evidenced by the abundance of PsaC and PSAD), and so does LHCII accumulation (LHCB2), while the PsbA (D1) subunit of PSII decreased due to its fast turnover (reviewed by Li et al. 2018). The putative PSAD degradation product is visible 4 d after the treatment, but is not more pronounced in *cepa1-3* than in the WT (Supplementary Fig. S5A). Therefore, the presence of the PSAD degradation product appears to be rather a consequence of photodamage (caused by the general PSI deficiency) than a specific subunit instability in the absence of CEPA1.

**Figure 4.**
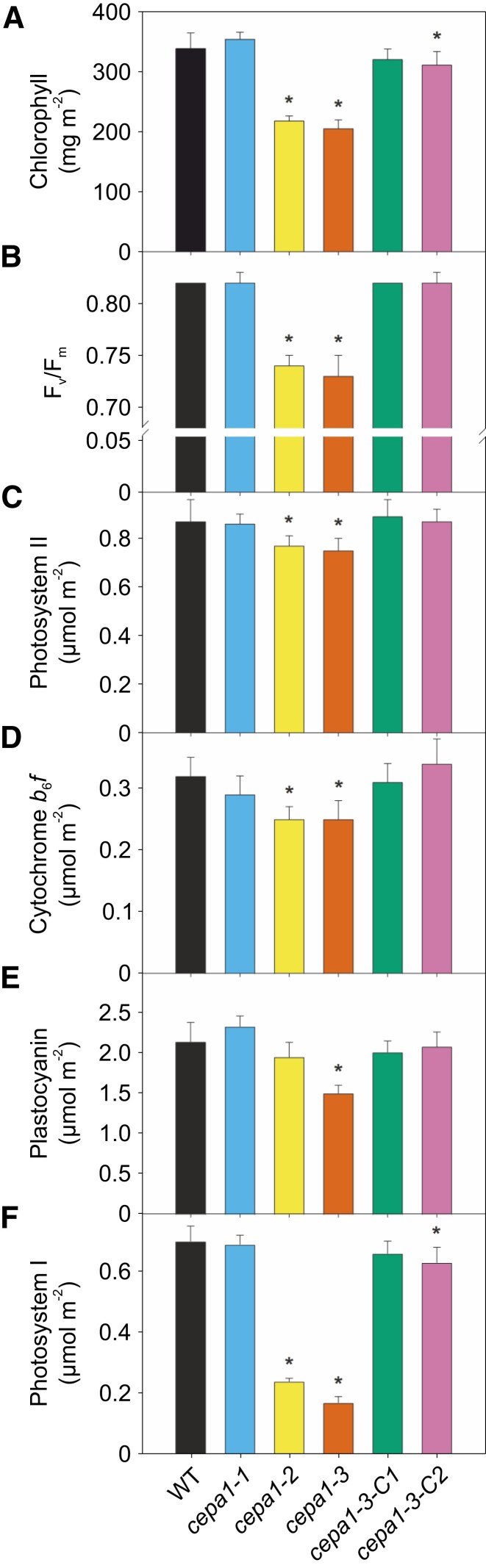
Analysis of chlorophyll contents and various photosynthetic parameters in the WT, three *cepa1* mutant lines and two complemented lines. **A)** Chlorophyll content per leaf area. **B)** Maximum quantum efficiency of PSII in the dark-adapted state (*F*_v_/*F*_m_). **C)** PSII content per leaf area. **D)** Cytochrome *b*_6_*f* complex content per leaf area. **E)** Plastocyanin content per leaf area. **F)** PSI content per leaf area. Thylakoid isolations performed for complex quantifications: *n* = 10 for the WT, *n* = 5 for *cepa1-1* and *n* = 6 for all other lines. Data were subjected to one-way ANOVA using a pairwise multiple comparison procedure (Holm–Sidak method) in SigmaPlot (version 14.5; Systat). Significant differences relative to the WT are indicated by asterisks (*P*-value < 0.05). Error bars represent the standard deviation (Sd).

We then conducted a detailed analysis of photosynthesis of plants grown under long-day conditions. The chlorophyll content per leaf area (Fig. 4A), the maximum quantum efficiency of PSII in the dark-adapted state (*F*_v_/*F*_m_; Fig. 4B), and the contents of PSII (Fig. 4C), the Cyt*b*_6_*f* complex (Fig. 4D), the lumenal redox carrier plastocyanin (Fig. 4E), and PSI (Fig. 4F) were determined and normalized to a leaf area basis. The *cepa1-1* line, which shows the least pronounced reduction in protein abundance, is indistinguishable in all parameters from the WT, in line with its normal growth (Fig. 1C). For the two more severely affected mutants, *cepa1-2* and *cepa1-3*, very similar defects are observed. Chlorophyll contents per leaf area are significantly reduced (by 35% and 40%) in the two mutant lines relative to the WT, and also *F*_v_/*F*_m_ is moderately but significantly decreased. Both PSII and the Cyt*b*_6_*f* complex show only small reductions in their contents (by 10% and 20%) relative to the WT, which is not visible in the immunoblots, whose loading was normalized to chlorophyll amounts (Fig. 3A). For plastocyanin, a significant reduction is only obtained for the most severely affected line *cepa1-3*. As expected, the PSI content is strongly reduced in both *cepa1-2* and *cepa1-3*, to 35% and 25% of the WT content, respectively. While *cepa1-3-C1* is indistinguishable from the WT in all analyzed parameters, in *cepa1-3-C2*, complementation is only partial, in that for both PSI and chlorophyll content per leaf area, small but significant reductions compared to the WT can still be detected (Fig. 4, A and F).

**Figure 5.**
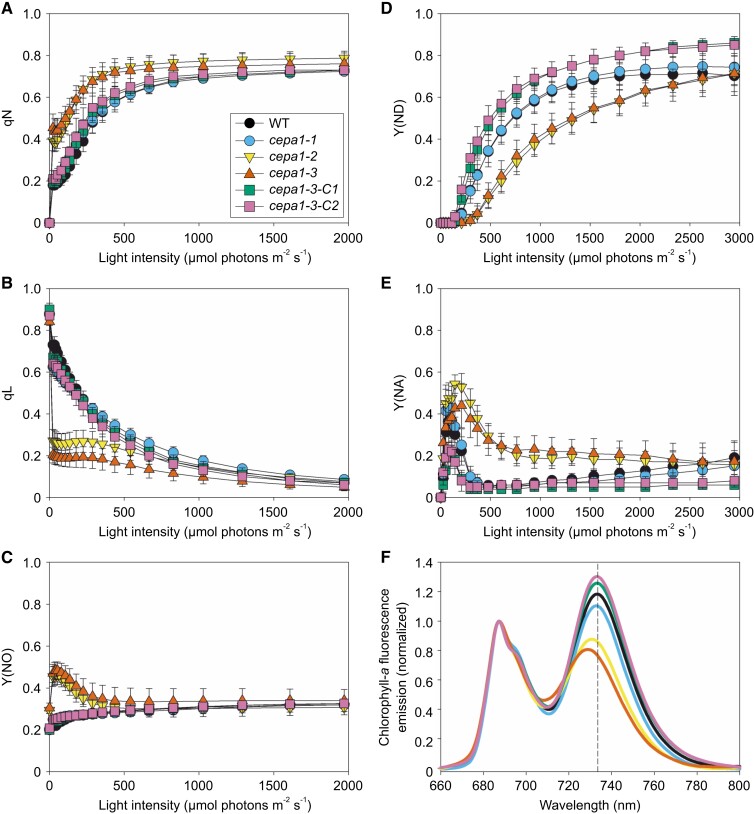
Functional parameters of photosynthesis in the WT, the three *cepa1* mutant lines and two complemented lines. **A)** LRC of nonphotochemical quenching (qN). **B)** LRC of the redox state of the PSII acceptor side (qL). **C)** LRC of nonregulated dissipation of excitation energy in PSII (Y(NO)). **D)** LRC of the donor-side limitation of PSI (Y(ND)). **E)** LRC of the acceptor-side limitation of PSI (Y(NA)). **F)** 77K chlorophyll-*a* fluorescence emission spectra of isolated thylakoids. The vertical dotted line marks the wavelength of 733 nm, where the maximum emission of PSI LHCI for intact PSI in WT is observed. For LRCs, individual plants were measured: *n* = 24 for the WT, *n* = 15 for *cepa1-1* and *n* = 18 for all other lines. For 77K fluorescence, same thylakoid isolations as in Fig. 4 were used. Error bars represent the standard deviation (Sd).

To assess the effect of reduced PSI content on functional parameters of photosynthesis, LRCs of the chlorophyll-*a* fluorescence parameters qN (a measure for photoprotective nonphotochemical quenching of excess excitation energy; Fig. 5A), qL (Fig. 5B), and Y(NO) (a measure for the nonregulated dissipation of excitation energy in PSII; Fig. 5C) were determined. Induction of qN is strongly shifted to lower light intensities in both *cepa1-2* and *cepa1-3*. Furthermore, in both mutant lines, qL decreases strongly in low light, in line with the previous observation made in the preliminary qL screening (Fig. 1B). With increasing light intensity, when electron transport between both photosystems is more and more rate-limited by the Cyt*b*_6_*f* complex, the qL curves of the WT and the *cepa1* mutants become increasingly similar. Finally, in low light, Y(NO) is increased in both *cepa1-2* and *cepa1-3*, likely because the stronger induction of qN cannot fully compensate for the overexcitation of PSII, relative to PSI, as illustrated by the qL LRC.

LRCs of the donor and acceptor side limitations of PSI (Y(ND) and Y(NA), respectively), show pronounced differences between *cepa1-2* and *cepa1-3* and the other lines. The onset of Y(ND), which indicates the accumulation of photo-oxidized P_700_^+^ due to insufficient reduction with electrons released by PSII via the Cyt*b*_6_*f* complex (Fig. 5D), is clearly shifted to higher light intensities in the mutants. This finding is consistent with the strong reduction in PSI content in both lines that is expected to result in a larger number of PSII, Cyt*b*_6_*f*, and plastocyanin per PSI, and thus leads to rapid re-reduction of photo-oxidized P_700_^+^. On the other hand, the acceptor-side limitation of PSI is clearly more pronounced in both *cepa1-2* and *cepa1-3* (Fig. 5E). Likely, due to the imbalanced excitation of the photosystems resulting in inefficient electron transport in low light, the reductive activation of the Calvin–Benson–Bassham cycle is impaired.

Finally, the antenna association with both photosystems was analyzed by 77 K chlorophyll-*a* fluorescence emission measurements (Fig. 5F). While in the WT, *cepa1-1* and both complemented lines, emission signals of PSI–LHCI with a similar maximum emission amplitude at 733 nm wavelength are observed, the amplitude of the signal is much lower in the *cepa1-2* and *cepa1-3* mutant lines, in agreement with their reduced PSI contents. The PSI–LHCI emission maximum is slightly shifted to lower wavelengths, indicating the presence of uncoupled LHCI, which emit between 700 and 730 nm wavelength. No shift of the emission maximum of PSII–LHCII or additional peaks corresponding to the presence of free LHCII are observed, demonstrating that, in line with *cepa1* mutations having only minor effects on PSII accumulation, no uncoupled LHCII accumulates.

Because PSI accumulation is not affected when CEPA1 is depleted to approximately half of the WT level (as in *cepa1-1*), the potential excess of CEPA1 may become relevant when growth conditions are more challenging. Therefore, we assessed whether the phenotypes of the *cepa1* mutants are more severe when grown in the cold, a condition known to induce PSI photoinhibition (Lima-Melo et al. 2021). While *cepa1-1* was still comparable to the WT in terms of development, PSI subunit accumulation and qL LRC (Supplementary Fig. S6), *cepa1-3* displayed pale emerging leaves, while the more developed leaf tissues are light green. The PSI subunits of these plants accumulate to levels below the detection limit of our immunoblots, but the qL LRC of *cepa1-3* is not null, indicating that PSI still accumulates to some extent. Given that the emerging leaf tissue is more severely affected, the strong decrease in PSI is likely attributable to slower assembly of PSI in *cepa1-3*.

Taken together, both biochemical and biophysical analyses suggest that CEPA1 is specifically involved in PSI core accumulation while other complexes are only very mildly, and likely indirectly, affected.

### CEPA1 acts post-translationally

**Figure 6.**
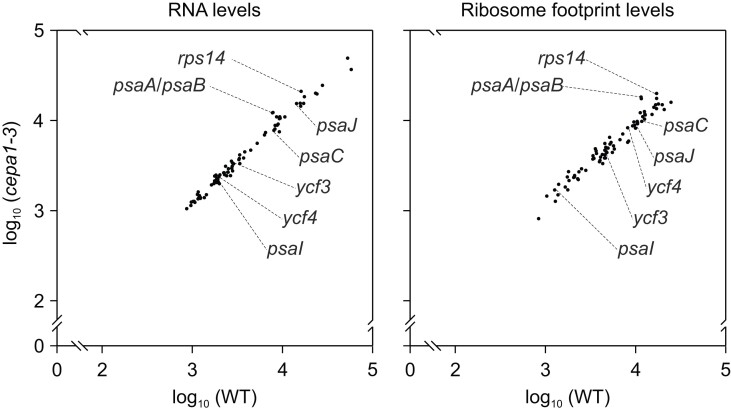
*CEPA1* knockout does not induce substantial defects in the expression of plastid-encoded genes. Chloroplast transcriptome and ribosome profiling in the WT and the *cepa1-3* null mutant. Both average RNA levels and ribosome footprint levels in chloroplasts of the WT and *cepa1-3* are plotted against each other. No major changes are observed in *cepa1-3* in comparison to the WT (including genes coding for the PSI subunits PsaC, PsaI, and PsaJ, and the PSI assembly factors Ycf3 and Ycf4), except for a slight upregulation of transcripts and ribosome footprints from the *psaA-psaB-rps14* gene cluster (possibly representing a regulatory feedback response to the PSI deficiency of the mutant). For more details, see Supplementary Data Set 1.

The PSI subunits PsaA-C, PsaI, and PsaJ (Schöttler et al. 2007, 2017) are encoded in the plastid genome. In addition, two PSI assembly factors, Ycf3 and Ycf4, are encoded in the plastid genome (Ruf et al. 1997; Krech et al. 2012). Therefore, a possible function of the chloroplast-localized CEPA1 in PSI-related plastid gene expression could explain the decrease of PSI in *cepa1* mutants. To address this possibility, chloroplast transcript accumulation and translation were analyzed in the WT and *cepa1-3* by microarray-based ribosome profiling (Fig. 6;Supplementary Data Set 1; Trösch et al. 2018). We did not observe any substantial change (of more than 2-fold) in plastid transcript accumulation or ribosome footprint levels between *cepa1-3* and the WT (Fig. 6). There is only a mild upregulation of *psaA*, *psaB*, and *rps14* gene expression in the mutant at the transcript and ribosome footprint levels, which, however, cannot account for the observed strong PSI defects, and presumably, solely represents a feedback response to the reduced PSI levels (Fig. 6). Also, at the level of translational efficiency the fold changes for all chloroplast genes are well below 2-fold in *cepa1-3* compared to the WT (Supplementary Fig. S7). Thus, the *CEPA1* knockout does not induce changes in the expression of chloroplast genes coding for PSI subunits and assembly factors that could explain its PSI deficiency. Hence, we conclude that CEPA1 affects PSI accumulation at the post-translational level.

### CEPA1 integrates into thylakoid membranes and is enriched in stroma lamellae

**Figure 7.**
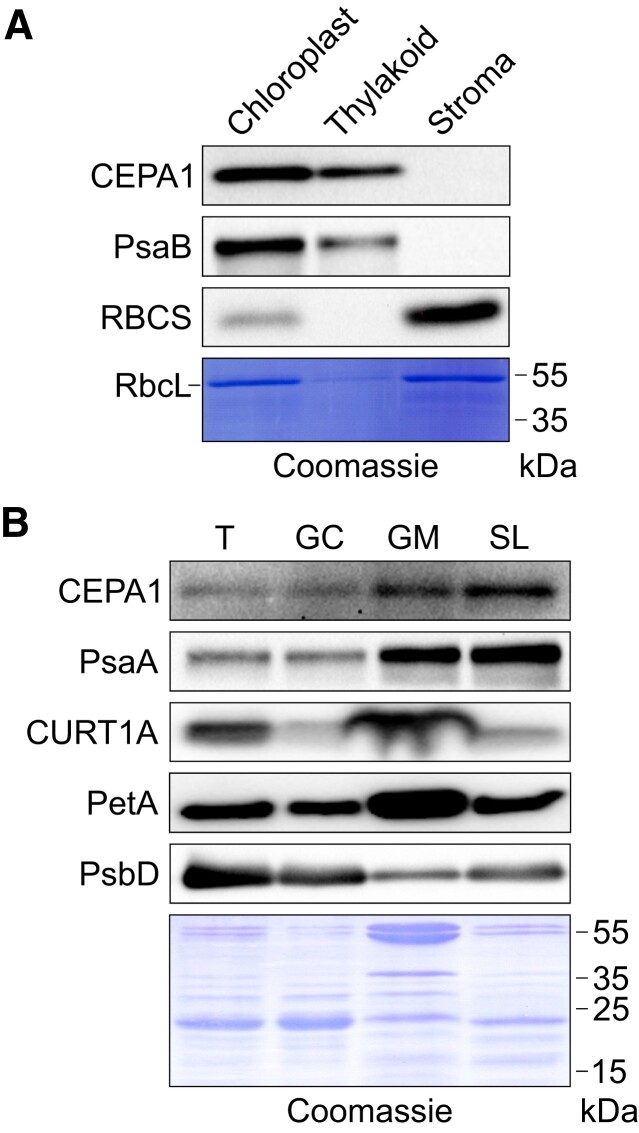
CEPA1 incorporates into thylakoid membranes and is enriched in nonappressed regions. **A)** Immunoblot analysis of chloroplast fractions. WT chloroplasts were isolated and fractionated into membranes and stroma. Proteins from intact chloroplasts, and membrane and stroma fractions (equivalent to 1 *µ*g chlorophyll for intact chloroplasts and the membrane fraction, and 10 *µ*g protein for the stroma fraction) were separated by SDS-PAGE and subjected to immunoblotting using antibodies against CEPA1 and selected marker proteins. CEPA1 accumulates in membranes, but not in the stroma. As expected, PsaB and RBCS are exclusively found in the membranes and stroma, respectively. The PVDF membrane used for immunoblotting was stained with Coomassie, and the region containing the band corresponding to the large subunit of RuBisCO (RbcL) is shown below the blots. **B)** Immunoblots with fractionated thylakoid samples. WT thylakoid membranes (T) were isolated and fractionated into grana cores (GC), grana margins (GM), and stroma lamellae (SL). Proteins from the total and fractionated thylakoid membranes (equal to 1 *µ*g chlorophyll) were separated by SDS-PAGE and subjected to immunoblotting with antibodies against CEPA1 and diagnostic thylakoid complex subunits. CEPA1 is enriched in nonappressed regions. As expected, PsaA (PSI) is enriched in nonappressed regions, CURT1A and PetA (Cyt*b*_6_*f*) in grana margins, and PsbD (PSII) in grana cores. The membrane used for immunoblotting was stained with Coomassie as a loading control.

As PSI resides in the thylakoid membranes, PSI assembly factors are assumed to be in close proximity to or embedded in the thylakoid membranes. As mentioned before, CEPA1 is predicted to be integrated into thylakoid membranes and was detected in thylakoid isolations by immunoblotting (Fig. 1D). To confirm that CEPA1 is a membrane-anchored protein, we purified WT chloroplasts and separated them into membrane and stroma fractions to be analyzed by immunoblot assays. These experiments confirmed that CEPA1 accumulates in the thylakoid membrane fraction, but not in the stroma (Fig. 7A). As controls, the intrinsic PSI subunit PsaB and the small subunit of RuBisCO (RBCS) were employed as markers for thylakoid membrane and stromal fractions, respectively.

The photosynthetic complexes are heterogeneously distributed within the thylakoid membranes, known as lateral heterogeneity (Anderson 2012). PSI accumulates in stroma lamellae and grana margins (Rantala et al. 2020). Data from a previous proteomic analysis of thylakoid subfractions suggest that CEPA1 is enriched in stroma lamellae (Tomizioli et al. 2014). To address the subthylakoid localization of CEPA1, thylakoid membranes from WT plants were fractionated into appressed and nonappressed membranes and the abundance of CEPA1 in the different regions was assessed by immunoblot analysis. Indeed, CEPA1 is enriched in the stroma lamellae, and to a certain extent, in the grana margins (Fig. 7B). Additionally, PsaA (a marker for PSI, mainly present in stroma lamellae and grana margins), CURVATURE THYLAKOID1A (CURT1A; mainly present in grana margins), PetA (a marker for Cyt*b*_6_*f*, mainly present in grana margins), and PsbD (a marker for PSII, mainly present in the grana core) were enriched in their respective fractions, as previously reported (Puthiyaveetil et al. 2014).

**Figure 8.**
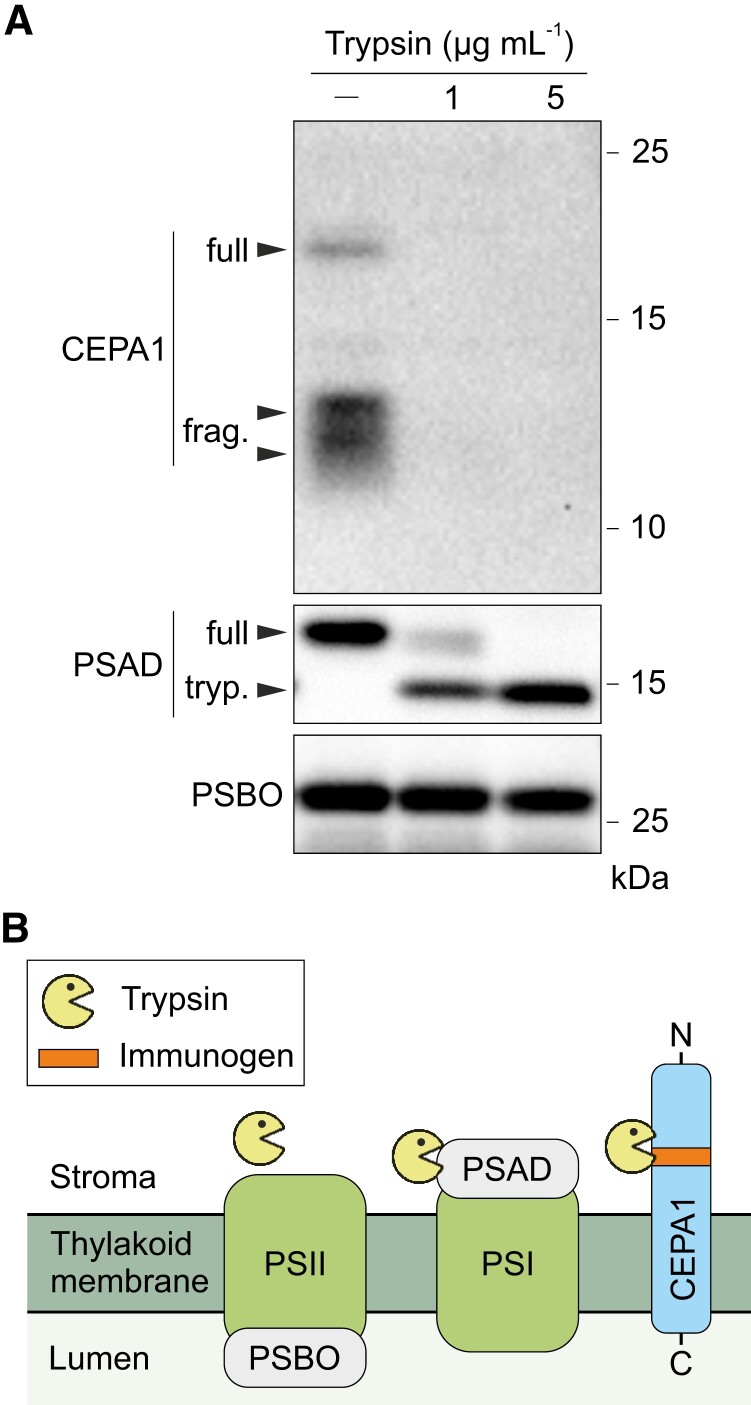
The N-terminal region of CEPA1 faces the stroma. **A)** Trypsin protection assay. Thylakoid membranes freshly isolated from WT leaves were incubated with 0, 1 or 5 *µ*g mL^−1^ trypsin for 30 min at 37 °C, and proteins (equal to 1 *µ*g chlorophyll) were subsequently separated by SDS-PAGE, transferred to PVDF membranes and immunodecorated with antibodies against CEPA1, PSAD (stromal protein) and PSBO (lumenal protein). PSBO remained intact, while a PSAD tryptic cleavage product (tryp.) was observed after trypsin treatment. The mature CEPA1 (full) and its potential fragments (frag.) were sensitive to the trypsin treatment. **B)** Scheme of the CEPA1 topology in thylakoid membranes. The immunogenic region of CEPA1 recognized by the specific antibodies (in orange) was not protected from trypsin digestion (neither in the full-length CEPA1 nor in its potential fragments), indicating that the N-terminal part of CEPA1 is exposed to the stroma. PSI, II: photosystems I and II.

Finally, we conducted a trypsin protection assay to reveal the topology of CEPA1 in the thylakoid membrane. To this end, WT thylakoid membranes were freshly isolated and subjected to trypsin treatment. The fate of PSBO (a lumenal protein), PSAD (a protein at the stromal surface), and CEPA1 was assessed by immunoblotting. Because trypsin has access to stromal but not lumenal domains, PSBO remained intact while PSAD was digested, as expected (Fig. 8A). CEPA1 was also digested by trypsin, suggesting that the region upstream of the transmembrane domain containing the immunogenic region used to generate the specific antibodies faces the stroma (Fig. 8, A and B). The two potential CEPA1 fragments were also sensitive to the trypsin treatment (Fig. 8A), suggesting that they contain peptides upstream of the transmembrane domain.

In conclusion, the nucleus-encoded CEPA1 translocates to the chloroplast, integrates into thylakoid membranes, accumulates mainly in the nonappressed regions where most of PSI is present, and exposes its N-terminal domain to the stroma.

### Organization of PSI and CEPA1-associated protein complexes

**Figure 9.**
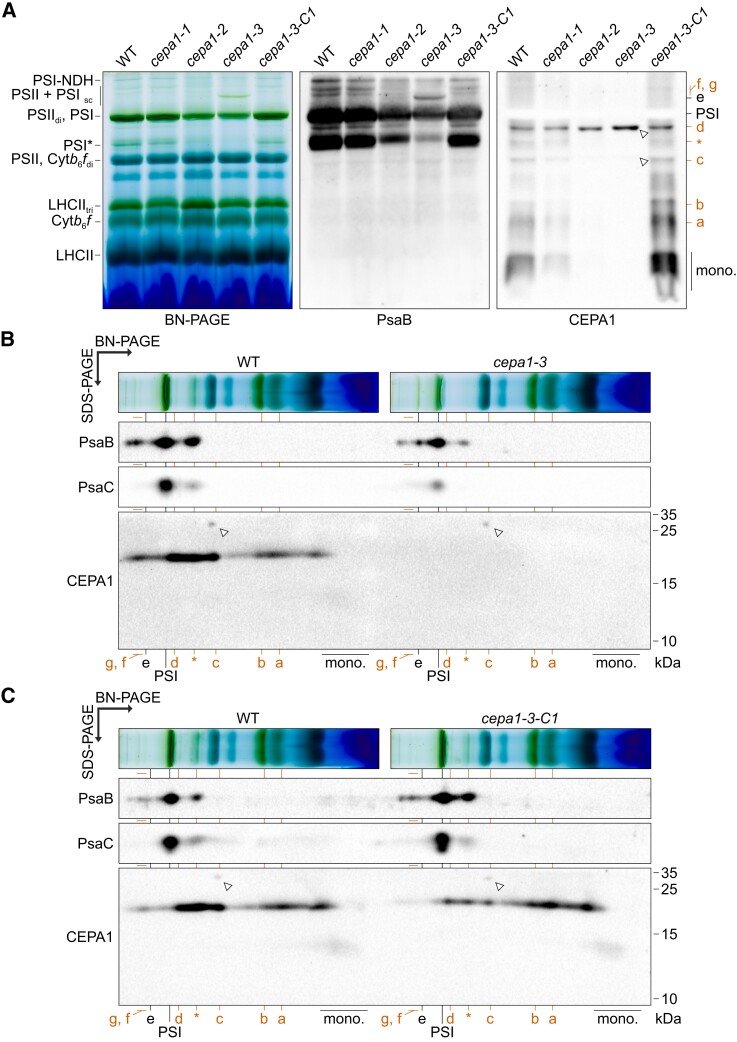
Organization of native thylakoid complexes solubilized in 1% (w/v) β-DM, and identification of CEPA1-associated complexes. Native complexes from thylakoid membranes (equal to 5 *µ*g chlorophyll) of 4-wk-old WT plants, T-DNA insertion mutants and a complemented mutant grown in short-day conditions were solubilized in 1% (w/v) β-DM and separated by BN-PAGE. **A)** Scan and immunoblot analyses to assess the PSI and CEPA1 accumulation profiles in BN-PAGE. The native gel was scanned (left panel) and subjected to immunoblotting with antibodies against PsaB (middle panel) and CEPA1 (right panel). The main complexes are identified from low to high molecular weight: protein monomers (mono.), LHCII monomers (LHCII), cytochrome *b*_6_*f* complex monomers (Cyt*b*_6_*f*), LHCII trimers (LHCII_tri_), photosystem II monomer (PSII), and Cyt*b*_6_*f* dimer (Cyt*b*_6_*f*_di_), photosystem I* intermediate (PSI*; Wittenberg et al. 2017), mature PSI–LHCI (PSI) and PSII–LHCII dimer (PSII_di_), PSII and PSI supercomplexes (PSII + PSI_sc_), and PSI–NDH megacomplex (PSI–NDH). The mature PSI–LHCI and PSI* are decreased in *cepa1-2* and *cepa1-3* in comparison to the WT. CEPA1 co-migrates with complexes marked by red letters (namely (a) to (c), PSI* (*), (d), (f), and (g), from low to high molecular weight). Complex (e) represents a complex containing PsaB but not CEPA1. Open triangles indicate cross-reaction signals (as evidenced by their presence also in the null mutant *cepa1-3*). **B, C)** Immunoblot analyses of the second-dimension profile of *cepa1-3* (B) and *cepa1-3-C1* (C), respectively, in comparison to the WT. First dimension lanes were reduced and denatured, and complex proteins from both lanes were separated next to each other in the same SDS-PAGE for the second dimension. After transfer of the proteins to a PVDF membrane, the membrane was cut, and the upper (>35 kDa) and lower (<35 kDa) parts were subjected to immunoblotting with antibodies against PsaB and CEPA1, respectively. A separate membrane was prepared for immunoblotting with antibodies against PsaC.

As all data described above pointed to decreased PSI accumulation in the *cepa1* mutants, we next investigated the native organization of thylakoidal protein complexes. To this end, thylakoid membranes of the WT, the *cepa1* mutants and the complemented lines were isolated, solubilized with 1% (w/v) dodecyl-β-D-maltoside (β-DM) and separated by blue-native PAGE (BN-PAGE; Fig. 9). The intensity of the green band corresponding to co-migrating PSII dimers and mature PSI–LHCI was found to be decreased in *cepa1-2* and more severely so in *cepa1-3* in comparison to the WT (“PSII_di_, PSI” in Fig. 9A). A weaker band which might represent the PSI* assembly intermediate described in tobacco (Wittenberg et al. 2017) is present in the WT, and is less abundant in the mutants (“PSI*” in Fig. 9A). In agreement with all previous data, the BN-PAGE profiles of *cepa1-1* and the complemented line *cepa1-3-C1* are similar to that of the WT (Fig. 9A).

Protein complexes were then transferred from the native gel to a membrane and immunodecorated with antibodies against PsaB, an essential subunit of the PSI reaction center. The PsaB signal intensity decreases similarly strongly in PSI and PSI* in *cepa1-2* and *cepa1-3* compared to the WT (Fig. 9A). The signals were confirmed to be specific to PsaB by separating the complex subunits in the second denaturing dimension followed by immunoblotting (Fig. 9B). Interestingly, the PSI*/PSI ratio does not seem to be increased in *cepa1-2* and *cepa1-3*, suggesting that PSI* is probably not the limiting step in the formation of mature PSI in the absence of CEPA1. Additionally, a PsaB-containing complex running slightly below the mature PSI–LHCI, and annotated here as complex (d), follows the same changes in signal intensity as PSI and PSI* in the different genotypes (Fig. 9A). This complex may represent another PSI assembly intermediate (rather than a degradation product of mature PSI–LHCI), given its accumulation pattern. On the other hand, there are PsaB-containing complexes that are larger than PSI–LHCI and annotated here as complexes (e), (f), and (g). They co-migrate with PSI and PSII supercomplexes (Fig. 9). Complex (e) is more abundant in *cepa1-3* than in the WT, while the opposite is the case for complexes (f) and (g) (Fig. 9, A and B).

Because CEPA1 was found to act at the post-translational level, we also examined the presence of CEPA1 in thylakoidal protein complexes by employing our antibodies against CEPA1. In the first dimension, several signals were detected, with the overall intensity following the trend of CEPA1 accumulation in *cepa1-1* (∼50% of WT levels), *cepa1-2* (<25% of WT levels), and *cepa1-3* (complete absence of CEPA1; Figs. 1D and 9A). There is a relatively strong signal in the free protein fraction in the WT and the complemented line, supporting the potential excess abundance of CEPA1 (“mono.” in Fig. 9A). The ratio of free CEPA1 to complex-associated CEPA1 seems to be reduced in *cepa1-1* compared to the WT. Since CEPA1 is depleted to half of the WT levels in *cepa1-1* (in the absence of a phenotype), a higher proportion of the total CEPA1 appears to be associated with complexes in this mutant, resulting in a smaller fraction of free CEPA1 than in the WT. Interestingly, CEPA1 seems to be associated with the assembly intermediate PSI* and other putative intermediate complexes in the range of 100 to 200 kDa, annotated here as complexes (a) and (b) (Fig. 9A). Two other signals, migrating near complexes (c) and (d), represent nonspecific cross-reactions, as they also appear in *cepa1-3* null mutant (open triangles; Fig. 9).

In the second dimension (2D SDS/BN-PAGE), CEPA1 was confirmed to co-migrate with complexes (a), (b), and (*) (Fig. 9B). The nonspecific signal observed near complex (c) is attributed to the 30 kDa-cross-reaction protein detected by CEPA1 antibodies (Figs. 1D and 9, B and C). Near this cross-reaction, there is a clear CEPA1-specific signal, which likely originates from complex (c), similar to what was observed in the first dimension. Interestingly, PPD1 was suggested to associate with the mature PSI–LHCI (Liu et al. 2012), but later proposed, together with PSA2 and PSAG, to be part of a complex with similar migration pattern as complex (c) (Fristedt et al. 2014; Roose et al. 2014). Notably, because the same membrane was cut in half for immunoblotting with the PsaB (upper part, >35 kDa proteins) and the CEPA1 (lower half, <35 kDa proteins) antibodies, the cross-reactions of ∼50 kDa usually detected by CEPA1 antibodies cannot be observed on this blot (Figs. 1D and 9, B and C). In spite of this, there is a CEPA1-size signal present at the level of complex (d) in the WT, which is even more clearly visible in *cepa1-3-C1* (Fig. 9, B and C). Interestingly, PSA3 was reported to co-migrate with a similar complex in maize (Shen et al. 2017). Additionally, even though PYG7 was proposed to co-migrate with the mature PSI–LHCI in sucrose gradients and native gels (Stöckel et al. 2006; Yang et al. 2017), the resolution does not permit to distinguish whether PYG7 associated rather with complex (d) than the mature PSI–LHCI. Since PSA3 and PYG7 cooperate in PSI assembly (Shen et al. 2017), PYG7 might also be associated with this complex instead of, or in addition to, the mature PSI–LHCI. PYG7 participates in the insertion of the essential subunit PsaC (Yang et al. 2017). However, despite the absence of CEPA1 from *cepa1-3*, the mutant survives on soil, and PsaC accumulation is not abolished and the subunit is present in the mature PSI–LHCI complex (Figs. 1C , 3A, and 9B). Therefore, CEPA1 is not essential for PsaC insertion into PSI. Unfortunately, none of the previously described antibodies against PPD1, PSA2, PYG7, and PSA3 gave a satisfying signal in immunoblot analyses to be able to confirm the putative co-migration of these proteins with CEPA1.

As expected, the *cepa1-3-C1* migration profile in BN-PAGE is similar to that of the WT, with recovery of PSI, PSI* and complex (d) amounts to WT levels, while complex (e) is virtually absent (Fig. 9, A and C). Also, CEPA1-FLAG is clearly present in complexes (a), (b), (c), (d), and PSI*, while its association with complexes (f) and (g) is less obvious in *cepa1-3-C1*.

**Figure 10.**
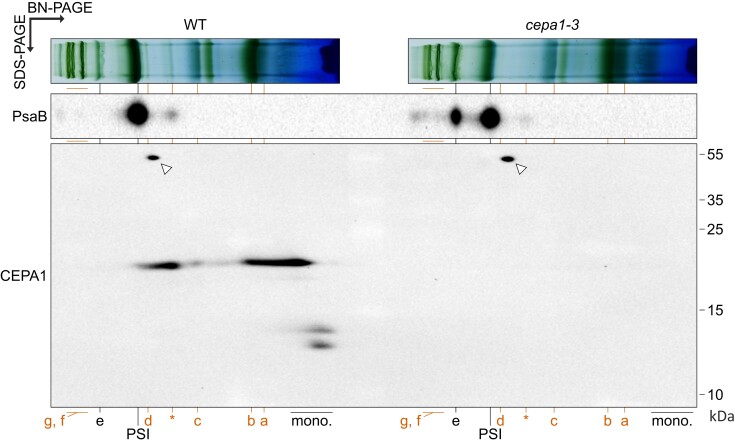
Native profile of the mature form and putative degradation fragments of CEPA1. Complexes from thylakoid membranes of 6-wk-old WT and *cepa1-3* plants grown in short-day conditions were solubilized in 1% (w/v) β-DM, and separated by BN-PAGE. The first-dimension lanes were then reduced and denatured, and complex subunits from both lanes were separated next to each other in the same SDS-PAGE for the second dimension, and subjected to immunoblotting with antibodies against PsaB (PSI core subunit) and CEPA1. The mature photosystem I–LHCI (PSI) is indicated. Red letters indicate the CEPA1-associating complexes (*cf.* Fig. 9). Note that there is clear segregation between the CEPA1 mature form associating mostly with complexes and the putative CEPA1 fragments (in the 10 to 15 kDa range) mostly found in the monomer fraction. Open triangles indicate cross-reaction signals (*cf.* Fig. 9).

In the second dimension, a relatively small proportion of CEPA1-FLAG migrates in the monomeric fraction in the WT and *cepa1-3-C1* compared to the observation in the first dimension (“mono.” in Fig. 9, A and C). In addition, there are traces of CEPA1 fragments (in the 10 to 15 kDa range) in the monomeric fraction (Figs. 1E and 9, B and C). Because the CEPA1 fragments were more abundant in older tissues (Fig. 1E), the 2D SDS/BN-PAGE profiles of thylakoid complexes from 6-wk-old WT and *cepa1-3* plants grown in short day conditions were examined (Fig. 10). Despite the cross-reaction signal near complex (c) being not visible on this blot, here the cross-reaction near complex (d) can clearly be attributed to the nonspecific signal of ∼50 kDa detected by the CEPA1 antibodies, and be distinguished from the CEPA1-specific signal in complex (d) (Figs. 1D and 10). As expected, PSI* is less abundant (relative to mature PSI–LHCI) than in younger material (Figs. 9B and 10). CEPA1 seems to co-migrate with complexes (a), (b), (c), (d), and PSI*, but not with complex (e). Again, signals for CEPA1 fragments in the 10 to 15 kDa range are detected in the monomer fraction, but not in mature CEPA1-associated complexes. Thus, there seems to be a clear segregation between the mature CEPA1 (mostly associating with complexes) and CEPA1 fragments (mostly present as monomers). Also, the accumulation of CEPA1 fragments relative to mature CEPA1 is increased in older material compared to in younger material (Figs. 9B and 10). Thus, proportionally more CEPA1 might be associated with complexes in young tissue for assembly of PSI subcomplexes, in agreement with the high importance of the PSI assembly machinery in young developing leaves. However, the accumulation of mature CEPA1 and CEPA1 fragments in thylakoid membranes rapidly decreased upon lincomycin treatment (that blocks the synthesis of plastid gene products including the essential PSI subunits encoded by *psaA*, *psaB*, and *psaC*). This observation may indicate a relatively fast turnover of CEPA1 and the instability of CEPA1 in the absence of de novo synthesized PSI subunits and/or PSI assembly intermediates (Supplementary Fig. S5B).

The above-described findings raise the question whether CEPA1 depends on active PSI biogenesis to associate with high-molecular weight complexes. We addressed this question by analyzing the CEPA1 migration pattern in native membranes from etiolated *cepa1-3*, *cepa1-3-C1* and WT seedlings, where no photosystems accumulate (Supplementary Fig. S8). In etiolated *cepa1-3-C1*, small amounts of CEPA1-FLAG were found to be anchored to the membranes. However, they are present only in the low-molecular weight region of the gel. The virtual absence of CEPA1 from the etiolated WT may be attributable to the low expression of *CEPA1* in the absence of light, when no PSI assembly takes place. Together, these results suggest that CEPA1 does not associate with larger complexes in the absence of active PSI biogenesis.

In conclusion, the *CEPA1* gene disruption leads to lower accumulation of mature PSI–LHCI, but does not completely abolish its assembly. Also, CEPA1 is not limiting for the transition from the PSI* intermediate to mature PSI. CEPA1 co-migrates with several complexes in BN-PAGE, including PSI*, but not the mature PSI–LHCI.

### Analysis of PSI–LHCI supercomplexes and subcomplexes

**Figure 11.**
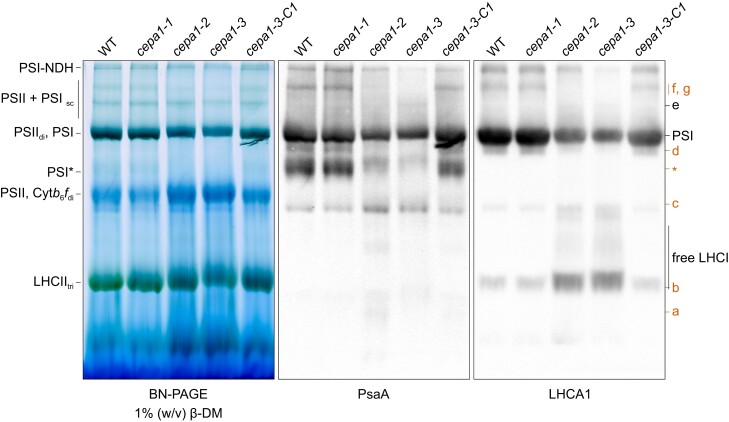
Native PSI and LHCI migration profiles in *cepa1* mutants and the WT. Native protein complexes from isolated thylakoid membranes (equal to 5 *µ*g chlorophyll) of the wild type (WT), the T-DNA insertion mutants and a complemented line were solubilized in 1% (w/v) β-DM and separated by BN-PAGE. The native gel was scanned (left panel) and subjected to immunoblotting with antibodies against PsaA (PSI core subunit, middle panel) and LHCA1 (LHCI subunit, right panel). The main complexes are (from low to high molecular weight): LHCII trimers (LHCII_tri_), PSII monomers (PSII), and cytochrome *b*_6_*f* dimers (Cyt*b*_6_*f*_di_), photosystem I intermediate PSI* (Wittenberg et al. 2017), mature photosystem I–LHCI (PSI) and photosystem II–LHCII dimers (PSII_di_), PSII and PSI supercomplexes (PSII + PSI_sc_), and PSI–NDH complex (PSI–NDH). Red letters indicate CEPA1-associated complexes (*cf.* Fig. 9). Complex (d) contains both the PSI core and LHCI subunits.

Because the CEPA1-associated complex (d) contains the PSI reaction center, and its accumulation decreases similarly to the mature PSI–LHCI and PSI*, we hypothesized that it may represent a PSI intermediate. Since the late PSI assembly intermediate described in Chlamydomonas was proposed to contain a loosely associated LHCI in vivo (Ozawa et al. 2010), we assessed whether LHCI subunits co-migrate with complex (d). Thylakoid membrane complexes from all lines were separated by BN-PAGE in a larger gel for better separation of PSI–LHCI and complex (d), followed by immunoblotting (Fig. 11). PsaA co-migrated with complex (d) and was decreased in the mutants relative to the WT, as had been observed with PsaB (Figs. 9A and 11). Interestingly, LHCA1 also co-migrated with complex (d) and followed a similar trend in intensity decrease as PsaA in the mutants (Fig. 11). As expected, both PsaA and LHCA1 co-migrated with the mature PSI–LHCI complex, and were decreased in *cepa1-2* and *cepa1-3* (“PSII_di_, PSI” in Fig. 11). Also, only PsaA co-migrated with PSI*, as LHCI was shown not to be part of this assembly intermediate (“PSII_di_, PSI” in Fig. 11; Wittenberg et al. 2017). In agreement with the 77K chlorophyll-*a* emission profiles (Fig. 5F), the amounts of free LHCI are increased in *cepa1-2* and *cepa1-3* compared to the WT (Fig. 11). Indeed, as LHCI accumulation is less affected than that of the PSI core in the mutants (Fig. 3), there is an excess of LHCIs relative to the PSI cores. With the currently available data, the strongest signal from the uncoupled LHCI cannot be attributed with certainty to complexes (a) or (b), even though it migrates in a similar size range (Figs. 9 and 11). The accumulation of complex (e) in *cepa1-3* is less pronounced in Fig. 11 than in Fig. 9A, but a faint signal corresponding to PsaA and LHCA1 at the level of this complex is observed in the immunoblots (Fig. 11).

**Figure 12.**
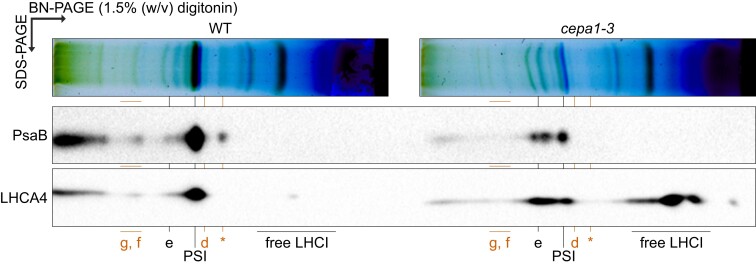
Second-dimension separation of digitonin-solubilized PSI and LHCI from *cepa1-3* and the WT. Thylakoid membranes (equal to 8 *µ*g chlorophyll) were isolated from the WT and *cepa1-3*, protein complexes were solubilized in 1.5% (w/v) digitonin, and separated in large pore BN-PAGE. First-dimension lanes were reduced and denatured, complex subunits from both lanes were separated next to each other in the same SDS-PAGE (as second dimension), and subjected to immunoblotting with antibodies against PsaB (PSI core subunit) and LHCA4 (LHCI subunit). Red letters indicate CEPA1-associated complexes (*cf.* Fig. 9). Note that complex (d) contains both PSI core and LHCI subunits.

Next, digitonin-solubilized thylakoid membranes from *cepa1-3* and the WT were separated in large pore BN-PAGE for better resolution of high molecular weight complexes, followed by 2D SDS/BN-PAGE and immunoblotting against the PsaB and LHCA4 (LHCI subunit) proteins (Fig. 12). Here, LHCA4 clearly co-migrates with the two PsaB-associated complexes near complex (e) in *cepa1-3* (Fig. 12). Since both complexes contain the PSI reaction center, one of them may correspond to complex (e), while the other one could represent the PSI–LHCI–LHCII supercomplex. The band that could represent complex (f) and/or (g) seems to also contain LHCA1 (Fig. 11), which would fit with the association of PSI–LHCI with other thylakoid complexes.

Therefore, as complex (d) appears to contain LHCI subunits, CEPA1-associated complexes may include both early and late PSI assembly intermediates.

### CEPA1 cooperates with PSA3 in PSI assembly

CEPA1 acts at the post-translational level (Fig. 6) and co-migrates with complexes in native conditions (Fig. 9). This suggests that CEPA1 likely interacts with other PSI-related proteins to mediate PSI assembly. We, therefore, conducted co-IP experiments followed by LC-MS/MS identification to capture potential CEPA1-interacting proteins. To this end, freshly isolated and solubilized thylakoid membranes of *cepa1-3-C1* and the WT were incubated with FLAG antibody-coated beads. The correct pull-down of CEPA1-FLAG was verified by immunoblotting (Supplementary Fig. S9). Not only the mature CEPA1-FLAG protein but also the two CEPA1-FLAG fragments (in the 10 to 15 kDa range) were strongly enriched in the eluates (El.) of *cepa1-3-C1*, and showed the expected size shift (resulting from the epitope tag) compared to the CEPA1 fragments from the nonbinding fraction (NBF) of the WT (Supplementary Fig. S9). This observation supports the hypothesis that these two signals correspond to fragments of CEPA1 with N-terminal truncations, as the C-terminal FLAG is still present. As expected, CEPA1 was not detected in the eluates of the WT sample (Supplementary Fig. S9).

The co-IP eluates were then processed by LC-MS/MS. Proteins were identified and quantified, and proteins were considered as significantly enriched if they were at least 2-fold more abundant in the *cepa1-3-C1* IP than in the WT IP (Table 2). Unsurprisingly, CEPA1 (i.e. CEPA1-FLAG in *cepa1-3-C1 vs.* native CEPA1 in the WT) was the most enriched protein in the *cepa1-3-C1* IP. Six other proteins were significantly enriched together with CEPA1-FLAG. Two of these proteins, TRANSLOCASE OF THE OUTER MITOCHONDRIAL MEMBRANE 40 (TOM40) and mitochondrial NADH dehydrogenase subunit 9 (Nad9), were considered as contaminants given that they are mitochondrial proteins. The other four proteins localize to the plastid. RIBOSOMAL PROTEIN L12-C (RPL12-C) is a plastid 50S ribosomal subunit and EMBRYO DEFECTIVE 3136 (EMB3136) is annotated as a ribosomal L10 family protein. As CEPA1 does not regulate plastid translation (Fig. 6), these two proteins are likely nonspecific interactors. Interestingly, the two PSI assembly factors PPD1 and PSA3 were also significantly enriched (Table 2). As mentioned above, CEPA1 might associate with the same complexes as PPD1 (complex (c) in Fig. 9; Roose et al. 2014) and PSA3 (complex (d) in Fig. 9; Shen et al. 2017) in BN-PAGE. Additionally, *PSA3* is directly linked to *CEPA1* in the *CEPA1* co-expression network from ATTED-II, in agreement with *CEPA1* being co-expressed with PSI assembly factors (Supplementary Fig. S10; Obayashi et al. 2022). However, no other PSI assembly factors or PSI subunits were significantly enriched in *cepa1-3-C1* IP experiments.

**Table 2.** Summary of proteins co-purified with CEPA1-FLAG

Alias	AGI locus	Localization	*P*-value		log_2_(LFQ)					
					*cepa1-3-C1* IP			Wild-type IP		
				FC	#1	#2	#3	#1	#2	#3
CEPA1	At3g56010	Plastid	<0.001	20.3	28.15	28.03	28.09	23.79	23.86	23.61
PPD1	At4g15510	Plastid	0.002	7.5	24.41	24.12	24.03	*20*.*75*	*22*.*04*	*21*.*06*
RPL12-C	At3g27850	Plastid	0.015	2.8	25.80	25.57	25.88	24.94	24.15	23.76
PSA3	At3g55250	Plastid	<0.001	2.7	22.00	22.00	22.18	20.52	20.77	*20*.*52*
EMB3136	At5g13510	Plastid	0.006	2.0	25.25	25.18	25.48	24.53	23.98	24.37
TOM40	At3g20000	Mitochondrion	0.044	4.9	23.25	23.28	23.26	*19*.*54*	22.28	*21*.*09*
Nad9	AtMg00070	Mitochondrion	0.013	2.9	23.93	23.54	23.25	22.65	21.76	21.73

Proteins in co-IP eluates were identified by mass spectrometry. Identified peptides were searched against a database including the *A. thaliana* proteome (Araport11), CEPA1-FLAG and common contaminants using the MaxQuant software (version 1.6.0.13). Protein abundance was assessed with the MaxLFQ algorithm, and “reverse sequences”, “proteins only identified by site”, and “low confidence proteins” were further filtered out. The enrichment analysis of proteins from *cepa1-3-C1* vs. the WT was done with the Perseus software (version 1.5.8.5), and was based on three biological replicates per line. Proteins were considered to be significantly enriched when they are at least 2-fold more abundant in the *cepa1-3-C1* IP than in the WT IP (i.e. fold change (FC) of LFQ intensity (*cepa1-3-C1* IP/WT IP) ≥ 2) with a Student's *t*-test *P*-value < 0.05. The log_2_(LFQ) values are indicated for each of the three biological replicates. The log_2_ values in italics were generated by computational imputation (from normal distribution of the total dataset) to replace missing values. For more details, see Materials and methods.

**Figure 13.**
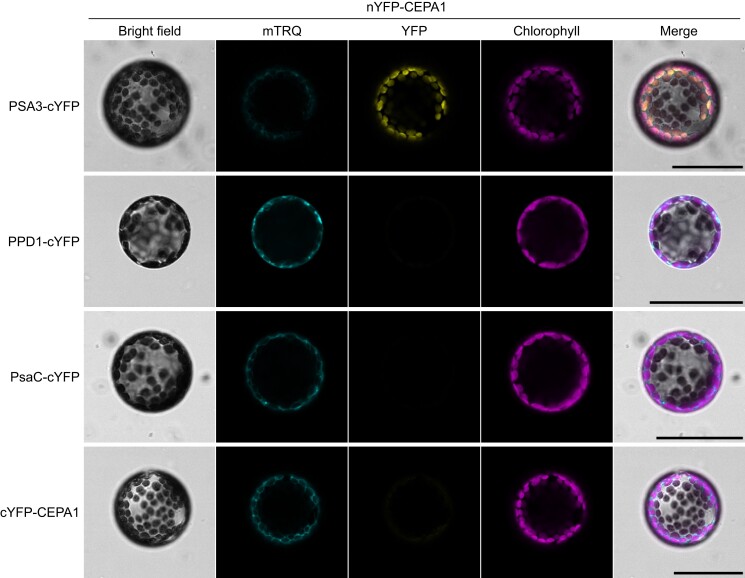
CEPA1 and PSA3 interaction revealed by BiFC assay. *N. benthamiana* leaves were infected with *A. tumefaciens* strains harboring the p19 silencing suppressor, the OEP7-mTRQ (fluorescent marker of the chloroplast envelope), the nYFP fusion to CEPA1, and the cYFP fusion to candidate interaction partners. After 3 d, protoplasts were prepared, and YFP fluorescence complementation was analyzed by confocal microscopy. For each sample, the bright field, mTRQ (cyan), YFP (yellow), and chlorophyll fluorescence (magenta) signals are shown. Among 50 observed protoplasts showing an OEP7-mTRQ signal, the CEPA1–PSA3 interaction gave a medium to strong YFP signal in nearly all of them. Scale bar: 50 *µ*m.

The direct interaction of CEPA1 with PSA3 and PPD1 was tested by bimolecular fluorescence complementation (BiFC) assays (Kudla and Bock 2016). To this end, we constructed a set of vectors each containing four cassettes to express (i) the RNA silencing suppressor p19, (ii) the OUTER ENVELOPE MEMBRANE PROTEIN 7-mTurquoise2 (OEP7-mTRQ; localizing to the chloroplast envelope) as a marker for transformed cells, (iii) the N-terminal YELLOW FLUORESCENT PROTEIN (YFP) moiety (nYFP: V2-D174) fused to the mature CEPA1, and (iv) the C-terminal YFP moiety (cYFP: G175-K239) fused to the mature interaction candidates. Because the mature sequences of the proteins of interest were used, the AtRBCS cTP sequence was cloned upstream of the fusion protein to confer targeting to the chloroplast. Individual *Nicotiana benthamiana* leaves were infiltrated with vector-harboring *Agrobacterium tumefaciens* cells, and 3 d later, protoplasts were prepared and observed by confocal microscopy. A clear YFP signal was observed for the nYFP-CEPA1/PSA3-cYFP binary test, while there was no to little signal in the nYFP-CEPA1/PPD1-cYFP test (Fig. 13). Because PPD1 is a lumenal protein, and the N-terminal domain of CEPA1 is in the stroma (Fig. 8B), we also assessed the possible CEPA1-nYFP/PPD1-cYFP interaction, but no YFP signal was observed either (Supplementary Fig. S11). The PSA3–PsaC interaction was previously supported by BiFC, but not by split-ubiquitin assays (Shen et al. 2017). We, therefore, also tested the interaction between CEPA1 and PsaC, which however, did not produce a YFP signal (Fig. 13). Finally, our BiFC data suggest that CEPA1 does not dimerize in thylakoid membranes (Fig. 13).

In conclusion, PSA3 and PPD1 were found in CEPA1-containing complexes by co-IP, and PSA3, but not PPD1, was confirmed to interact directly with CEPA1 in BiFC assays.

## Discussion

PSI biogenesis in thylakoid membranes is mediated by an intricate assembly machinery composed of plastid-encoded and nucleus-encoded proteins that participate in the proper folding, insertion, and stabilization of the different PSI subunits. Because the action of this machinery needs to be highly coordinated in time and space, genes which are co-expressed with known PSI assembly factors represent putative candidates for players participating in PSI biogenesis. In this work, genes that are predicted to be functionally related to the small set of known nucleus-encoded PSI assembly factors were obtained. The list was narrowed down to 22 genes coding for plastid-localized proteins with uncharacterized functions, and with T-DNA insertion lines available (Supplementary Table S1). To select putative candidates involved in PSI assembly, T-DNA insertion lines were subjected to a qL screening by chlorophyll-*a* fluorescence imaging. Mutants for one of the candidates, named *CEPA1*, displayed qL LRCs typical of low PSI accumulation mutants (Fig. 1B; Albus et al. 2010). The three *cepa1* T-DNA insertion lines express ∼50% (*cepa1-1*), <25% (*cepa1-2*), and 0% (*cepa1-3*) of CEPA1 WT levels (Fig. 1D). Because the *cepa1-1* phenotype is indistinguishable from the WT, CEPA1 may be present in excess in the plant, at least under the growth conditions tested here (Fig. 1B; Supplementary Fig. S6). Growth and development of *cepa1-2* and *cepa1-3* are delayed, and leaves are paler than in the WT as a result of inefficient photosynthetic electron transfer (Figs. 1C, 4, and 5). Expression of CEPA1-FLAG in the *cepa1-3* background is sufficient to restore WT-like PSI accumulation and, consequently, photosynthetic activity and normal plant development (Figs. 1C, 4, and 5). The *cepa1* mutant phenotype was linked to a specific decrease in PSI accumulation, and we propose here that CEPA1 is a PSI assembly factor.

### CEPA1 is specifically required for PSI accumulation

All photosynthetic defects in the strongly affected mutant lines *cepa1-2* and *cepa1-3* can be explained by a reduced accumulation of PSI (Figs. 3 and 4F). The possibility of defective plastid PSI-related gene expression at the transcriptional and/or post-transcriptional levels in the absence of CEPA1 was largely excluded by the transcript profiling and ribosome profiling assays conducted (Fig. 6). The mild upregulation of *psaA* and *psaB* expression and ribosome footprint levels in *cepa1-3* is likely caused by the mutant's response to the deficiency in PSI accumulation. The *rps14* gene follows the same trend, as it is part of the *psaA-psaB-rps14* gene cluster. Thus, CEPA1 participates in the accumulation of PSI at the post-translational level, i.e. in PSI assembly and/or stabilization.

No indications exist for an impaired function of the residual PSI complexes in the mutants. For example, any defect in plastocyanin binding and/or oxidation should result in an onset of Y(ND) already in low light, while in the mutant plants, the onset of Y(ND) was shifted to higher light intensities, in line with a higher number of PSII and Cyt*b*_6_*f* complexes per PSI, leading to its more efficient reduction (Fig. 5D). Similarly, the more pronounced Y(NA) under light-limited conditions is incompatible with a defect in ferredoxin binding and reduction, because in that case, differences in Y(NA) would be expected to become even more pronounced with increasing light intensities and electron transport rates (Fig. 5E).

### CEPA1 co-localizes with PSI and associates with PSI subcomplexes

CEPA1 codes for a protein harboring a cTP (M1-C51) that translocates the protein to the chloroplast, and is anchored in thylakoid membranes by a single transmembrane domain (Figs. 1A and 7A; Supplementary Fig. S1B). CEPA1 co-localizes with PSI in nonappressed membranes, consistent with a function linked to this complex (Fig. 7B). The presence of its long N-terminal region (L52-D169) in the stroma and its relatively short C-terminal tail (K193-E201) in the lumen suggest that CEPA1 acts at the stromal side in PSI assembly (Fig. 8).

In native gels, CEPA1 co-migrates with several complexes that contain PSI subunits and/or assembly factors, including the PSI* assembly intermediate previously described in tobacco (Fig. 9; Wittenberg et al. 2017). Complex (d) is slightly smaller than the mature PSI–LHCI and may contain both PSI and LHCI subunits, even though the resolution in the second dimension does not allow us to unambiguously distinguish complex (d) from mature PSI–LHCI (Figs. 11 and 12). Several observations of PSI assembly factors co-migrating with a complex of similar size (Ycf4, PPD1, PYG7, and PSA3) were previously reported in land plants (Krech et al. 2012; Liu et al. 2012; Shen et al. 2017; Yang et al. 2017). The PSI core is surrounded by an LHCI belt composed of 4 and 10 LHCAs in land plants and Chlamydomonas, respectively (Mazor et al. 2017; Ozawa et al. 2018). Despite this difference in structural organization, complex (d) might represent the PSI intermediate previously described in Chlamydomonas (Ozawa et al. 2010). Indeed, it was proposed that this PSI intermediate associates loosely with LHCI in vivo, and then becomes stabilized after insertion of PSAG and PSAK. Also, this complex represents a later intermediate than PSI*, as it contains the PSAF subunit that is critical for the transition of PSI* to mature PSI–LHCI (Wittenberg et al. 2017).

CEPA1 was also found in complex (c) of ∼250 kDa, migrating near the PSII monomer and the Cyt*b*_6_*f* in native gels. The PSI assembly factors PPD1 and PSA2 in Arabidopsis, and to a lesser extent, PSA3 in maize, associate with a complex of similar size (Fristedt et al. 2014; Roose et al. 2014; Shen et al. 2017). Additionally, CEPA1 also associates with complexes (a) and (b) in the range of 100 to 200 kDa. A complex of ∼160 kDa was previously suggested to correspond to the PsaA–PsaB heterodimer, as PsaA/PsaB-size proteins were identified in this complex in a pulse-chase experiment (Yang et al. 2017). However, while the PsaA–PsaB apoproteins would indeed account for 160 kDa, the reaction center with all co-factors would be around 255 kDa, which is closer to complex (c), but no co-migration of PsaB with this complex was detected (Fig. 9, B and C). Thus, complex (c) may not contain PSI subunits, but could still be relevant for PSI assembly, for example, by representing a module composed of PSI assembly factors. However, it should be noted that the estimate of complex sizes in blue-native gels can differ from the theoretical complex sizes, especially when hydrophobic proteins come into play.

CEPA1 also interacts with complexes (f) and (g), which migrate in the range of PSII and PSI supercomplexes. Because they contain PsaB, they could represent PSI assembly complexes composed of PSI subunits and assembly factors, similar to the large Ycf4–PSI assembly complex of 1,500 kDa characterized in Chlamydomonas (Ozawa et al. 2009). However, while Ycf4 oligomerizes in vivo to form a scaffold (Nellaepalli et al. 2021), potentially leading to a very large structure, CEPA1 does not seem to dimerize (Fig. 13). It also seems possible that the large complexes (f) and (g) correspond to PSI–LHCI associations with other thylakoid-embedded complexes. On the other hand, complex (e) overaccumulates in *cepa1-3*, but does not co-migrate with CEPA1. It may represent the PSI–LHCI–LHCII supercomplex, as an increase in this complex was previously observed in PSI-deficient mutants due to imbalanced light harvesting (Pesaresi et al. 2009; Roose et al. 2014).

The identification of nascent PSI subunits that may associate with CEPA1 would be an important step toward resolving the precise molecular mode of action of the CEPA1 protein. Unfortunately, despite multiple attempts, no enrichment of specific PSI subunits in CEPA1-FLAG co-IP assays was observed, despite testing of a range of different IP conditions (including the use of crosslinking agents, and tests of different washing and elution buffers, incubation times and temperatures). This failure could be attributable to physiological reasons (e.g. a weak and/or very short-lived interaction of CEPA1 with PSI subunits) and/or technical limitations (e.g. the position and nature of the epitope tag, or conformational changes of the bait upon crosslinking). The further optimization of the IP assays and the exploration of alternative methods to test for physical interactions of CEPA1 with PSI subunits will be a focus of future research.

### Targeted degradation of CEPA1 as part of its function?

Antibodies raised against CEPA1 recognized, in addition to the full-length protein, two specific signals within the 10 to 15 kDa range (Fig. 1E). These two CEPA1 fragments were also isolated in co-IP experiments with the *cepa1-3-C1* complemented line (Supplementary Fig. S9). Thus, they consist of at least (i) the C-terminal part, because they still possess the FLAG tag, (ii) the transmembrane domain, and (iii) the antigenic region recognized by our CEPA1-specific antibodies, as they are visible in immunoblots with isolated thylakoid membranes. The CEPA1 fragments accumulate in the monomeric protein fraction in BN-PAGE, as opposed to mature CEPA1 which co-migrates with high-molecular-weight complexes (Fig. 10), possibly suggesting that the fragments represent degradation products. We, therefore, speculate that the mature CEPA1 transiently associates with PSI assembly complexes, and dissociates from these complexes after its N-terminal region is cleaved off. The exact site of this cleavage remains to be determined.

### Cooperation of CEPA1 with other eukaryotic PSI assembly factors

Both PPD1 and PSA3 were co-purified with CEPA1 by co-IP (Table 2). Given the orientation of CEPA1 in the thylakoid membrane, it is not surprising that the stromal PSA3 but not the lumenal PPD1 was shown to interact directly with CEPA1 by BiFC (Figs. 8B and 13). Interestingly, in the *CEPA1* co-expression network extracted from the ATTED-II database (Obayashi et al. 2022), *CEPA1* and PYG7 are linked to PSA3 (Supplementary Fig. S10). This network is consistent with the cooperative action of PSA3 and PYG7 at the stromal side of PSI (Shen et al. 2017). Despite being conserved in land plants and Chlamydomonas (Fig. 2), CEPA1 is not part of the GreenCut2 list that contains the nucleus-encoded proteins that are specifically conserved in photosynthetic eukaryotes (Karpowicz et al. 2011). Of all previously described nucleus-encoded PSI assembly factors, only PSA3, despite being conserved in Chlamydomonas, is not present in this set, presumably due to its relatively high sequence divergence (Shen et al. 2017).

The putative CEPA1 homolog in Chlamydomonas identified in this work is encoded by locus Cre01.g014000 (Supplementary Fig. S4). In a screen for mutants in photosynthesis, Li et al. (2019) classified Cre01.g014000 as one of the 21 “higher-confidence genes without a known function in photosynthesis”, while Fauser et al. (2022) clustered *CrCEPA1*/*LGS1* with the “non-photoautotrophic light sensitive” genes, many of which code for proteins involved in the biogenesis or regulation of the photosynthetic machinery. Additional lines of evidence point to Cre01.g014000 coding for a genuine homolog of AtCEPA1. Mackinder et al. (2017) generated Chlamydomonas strains overexpressing tagged PSBP4, the homolog of PPD1 from Arabidopsis, and conducted co-immunoprecipitation (co-IP) assays. Interestingly, CrCEPA1/LGS1 was identified among the co-purified proteins, in agreement with AtPPD1 being pulled down in AtCEPA1-FLAG co-IP in our present study. Thus, PPD1, although probably not interacting directly with CEPA1, may still be part of a CEPA1-containing PSI assembly complex, likely without making direct contact with CEPA1. Finally, in a very recent study, the proteomic and transcriptomic profile of Chlamydomonas mutants defective in photosynthesis revealed that disruption of *CrCEPA1/LGS1* leads to a specific decrease in PSI subunit accumulation, but not in *psaA* transcript maturation (and the gene was named *PHOTOSYSTEM I REQUIRED 1*, *PIR1*; Kafri et al. 2023). However, in line with our primary BLASTp search using the AtCEPA1 sequence, none of the aforementioned studies could associate any Arabidopsis homolog to CrCEPA1/LGS1/PIR1 (Mackinder et al. 2017; Li et al. 2019; Fauser et al. 2022; Kafri et al. 2023). The precise molecular function of CrCEPA1/LGS1/PIR1 in Chlamydomonas remains unclear.

The conservation of CEPA1, PSA3, and PPD1 in photosynthetic eukaryotes, but not prokaryotes, suggests that their function is specific to eukaryotic PSI assembly. However, accumulation of the PSI subunits PSAG, PSAH, PSAN, and PSAO, that were newly acquired in eukaryotes, does not seem to be affected more strongly in the mutants than that of all other subunits (that are also present in prokaryotic PSI; Fig. 3A). The CEPA1 interaction partner PSA3 cooperates with PYG7, which is conserved from prokaryotes to eukaryotes (Wilde et al. 2001; Stöckel et al. 2006; Shen et al. 2017; Yang et al. 2017). Together, PSA3 and PYG7 participate in the insertion of the essential PSI subunit PsaC in the early steps of PSI assembly (Shen et al. 2017; Yang et al. 2017). CEPA1 is unlikely to participate in PsaC insertion, given that the *cepa1-3* null mutant does not lose photoautotrophy (Fig. 1C), and still accumulates PsaC in mature PSI–LHCI (Fig. 9B), and CEPA1 does not interact directly with PsaC (Fig. 13). How other PSI assembly factors respond to the absence of CEPA1 is currently unknown. Preliminary mass spectrometric analyses of total protein samples from *cepa1-3*, *cepa1-3-C1* and the WT failed to reproducibly detect most of the known PSI assembly factors, with the exception of PSA3, which was consistently detected in all four replicates of each genotype. PSA3 seems to be slightly upregulated in *cepa1-3* compared to the WT and *cepa1-3-C1*, although more thorough protein quantification will have to be conducted in the future. Similarly, investigating CEPA1 accumulation in the absence of PSA3 may provide additional insight in the relationship between the two assembly factors.

### A working model for CEPA1 function in PSI assembly

**Figure 14.**
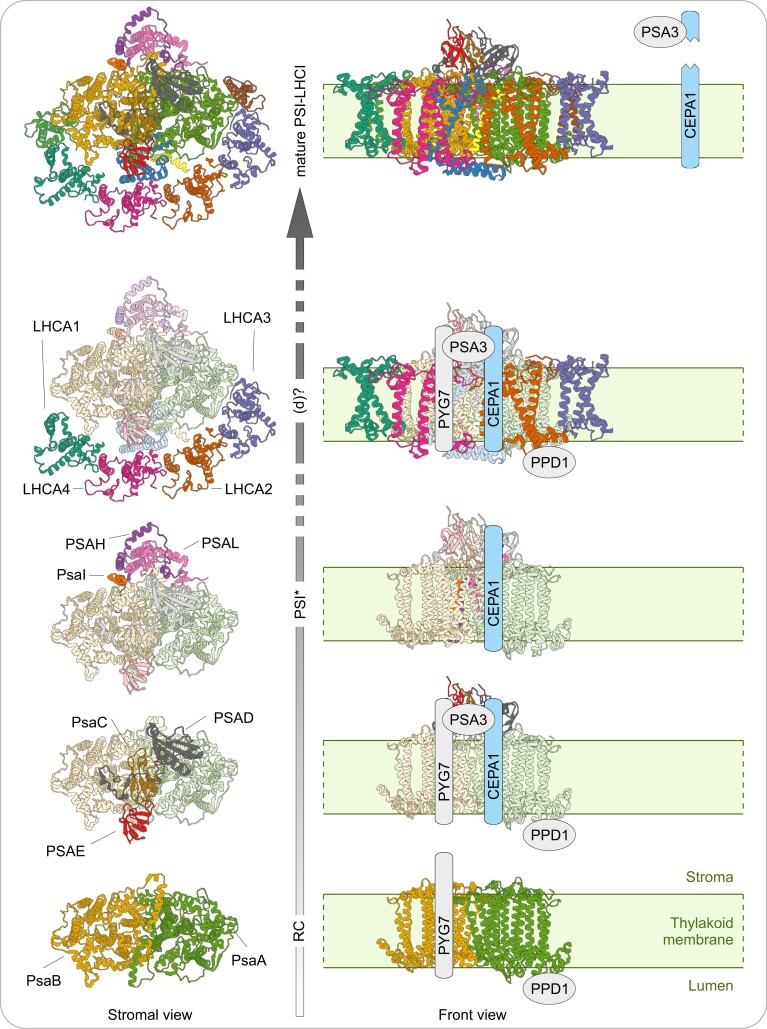
Proposed model of CEPA1 function in PSI assembly. The PSI–LHCI complex and its subunits are represented according to the three-dimensional structure from *P. sativum* (PDB ID: 5L8R; Mazor et al. 2017) available in the RCSB PDB website (Berman et al. 2000). No PSI subunit could be identified as a possible client protein of CEPA1. Instead, CEPA1 was co-immunoprecipitated with PSA3 and PPD1, and PSA3 interacts directly with CEPA1 in BiFC assays. Thus, CEPA1 may anchor PSA3 to the sites of PSI assembly in the thylakoid membrane. In addition, CEPA1 was found to co-migrate with the PSI assembly intermediates PSI* and complex (d) in native gels, suggesting its direct or indirect involvement in multiple steps in PSI assembly after insertion of the reaction center heterodimer. Finally, CEPA1 is likely cleaved out of the nascent PSI complex prior to maturation into the final PSI–LHCI. RC: PSI reaction center. See text for details.

Based on the observations discussed above, we propose a model for CEPA1 function in PSI assembly (Fig. 14). CEPA1 integrates in thylakoid membranes and may associate with several complexes, at least some of which represent PSI assembly intermediates, including PSI*. Given the general defect in PSI assembly in the mutants and the association of CEPA1 with at least two intermediate PSI complexes (i.e. PSI* and complex (d)), CEPA1 may be involved in multiple steps in PSI assembly, possibly from PSI reaction center formation to the final steps of PSI–LHCI assembly. However, CEPA1 is not associated with mature PSI–LHCI (Figs. 9 and 10), consistent with a function as an assembly (rather than a stability) factor. In the absence of CEPA1, still 25% of mature and fully functional PSI–LHCI can be assembled, and none of the PSI core subunits appear to be absent. Also, LHCI is only mildly affected in the mutants. Thus, while not being absolutely essential for PSI biogenesis, CEPA1 may facilitate the insertion of PSI core subunits. It also seems possible that CEPA1 anchors soluble PSI assembly factor(s) to the sites of PSI assembly in the thylakoid membrane. Indeed, given the direct interaction of the soluble PSA3 with CEPA1, and of PSA3 with PYG7, CEPA1 may stabilize PSA3 either in cooperation or in competition with PYG7. PYG7 is thought to participate in the early steps of PSI assembly by protecting the stromal side of the PsaA–PsaB heterodimer, followed by insertion of PsaC as part of the stromal ridge, in cooperation with PSA3 (Shen et al. 2017; Yang et al. 2017; Nellaepalli et al. 2021). PPD1 has also been suggested to participate in the early steps of PSI assembly by binding PsaA and PsaB from the luminal side (Liu et al. 2012; Roose et al. 2014). CEPA1 could interact with PSA3 in a large complex that also contains PPD1, thus potentially explaining why PPD1 was identified by co-IP, but did not directly interact with CEPA1 in BiFC assays (Table 2 and Fig. 13). PYG7, PSA3, PPD1, and CEPA1 associate with a PSI subcomplex that is slightly smaller than the mature PSI–LHCI (Stöckel et al. 2006; Liu et al. 2012; Shen et al. 2017), and is annotated here as complex (d). This complex may be a late PSI assembly intermediate containing loosely associated LCHI subunits, as observed in Chlamydomonas (Ozawa et al. 2010). Finally, we propose that, when CEPA1 has fulfilled its function, part of its stromal N-terminal domain is cleaved off, thus detaching CEPA1 (and potentially PSA3) from the last PSI assembly complex it is associated with. Whether or not PSA3 accumulation and attachment to thylakoid membranes is affected in the absence of CEPA1, is an interesting question that will need to be addressed by generation of functional antibodies against PSA3 in Arabidopsis, or the construction of transgenic plants expressing a tagged PSA3 protein in the *cepa1-3* mutant background. It also will be important to further characterize the different CEPA1-associated complexes revealed in this study and to assess whether CEPA1 interacts with PSI subunits or additional PSI assembly factors.

In summary, our work described here has identified a membrane-bound PSI assembly factor that (i) is specific to photoautotrophic eukaryotes, (ii) acts in multiple assembly intermediates, and (iii) cooperates with the soluble assembly factor PSA3 to assist in PSI biogenesis at the stromal side of the thylakoid membrane.

## Materials and methods

### Plant material and growth conditions

The Arabidopsis (*A. thaliana*) WT ecotype Col-0 was used as a control. The T-DNA insertion lines *cepa1-1* (SAIL_607_B08 (N825971)), *cepa1-2* (SAIL_747_E01 (N833366)), and *cepa1-3* (GABI_257C05 (N424605)) were selected from the T-DNA express website (http://signal.salk.edu/cgi-bin/tdnaexpress; SAIL lines: Sessions et al. 2002; GABI-Kat lines: Kleinboelting et al. 2012), and ordered from the Nottingham Arabidopsis Stock Centre (NASC; https://arabidopsis.info/; Scholl et al. 2000). Homozygosity of T-DNA insertion lines was confirmed by polymerase chain reaction (PCR) amplification (PCRs 1-4 in Supplementary Table S4) and sequencing of the T-DNA insertion site, with primers generated with the iSect Primers tool (http://signal.salk.edu/tdnaprimers.2.html; O’Malley et al. 2015).

If not otherwise indicated, Arabidopsis plants were grown as follows. Seeds were soaked in germination solution (0.1% (w/v) gibberellic acid, 0.1% (w/v) agarose) in the dark at 4 °C for 2 d and then sown in pots filled with soil. Germinated seedlings were grown for 1 wk in long-day conditions (in a 16 h light/8 h dark cycle, light intensity of light-emitting diodes (LEDs; Valoya): 150 *µ*mol photons m^−2^ s^−1^, temperature: 21 °C during the day; 19 °C during the night) followed by 1 wk in short-day conditions (8 h light/16 h dark cycle). Seedlings were then grown in long-day or short-day conditions as indicated. Under all growth conditions, the light intensity was set to 120 *µ*mol photons m^−2^ s^−1^, and temperature was 21 °C during the day and 19 °C at night.

For preliminary screening of mutants based on the qL LRC by pulse-amplitude modulation (PAM) imaging (referred to as qL screening), seeds were surface-sterilized in chlorine gas for 4 h. Sterile seeds were sown in plates containing 0.5× MS agar medium (Murashige and Skoog 1962) supplemented with 1% (w/v) sucrose. Plates were placed for 2 d in the dark at 4 °C, and then moved to a Percival cabinet with a 16 h light/8 h dark cycle (light intensity of LEDs: 100 *µ*mol photons m^−2^ s^−1^, temperature: 21 °C during the day; 19 °C during the night) for germination and growth. After 10 d, seedling phenotypes were assessed by qL screening, followed by transfer to soil. For selection of the complemented lines *cepa1-3*-*C1* and *cepa1-3-C2*, the T1 population was grown on 0.5× MS medium supplemented with 10 *µ*g mL^−1^ phosphinothricin (PPT), and resistant lines were further selected by qL screening. Two independent T1 transformants, *cepa1-3-C1* and *-C2*, were kept for seed propagation, and biochemical characterization was done with the T3 generation. The presence of the T-DNA harboring the *CEPA1-FLAG* expression cassette was assessed by PCR and confirmed by DNA sequencing (PCR 7 in Supplementary Table S4).

### Nucleic acid isolation and PCR

The extraction of genomic DNA (gDNA) from Arabidopsis leaf material was performed with the Extract-N-Amp solutions (Sigma–Aldrich). For each gDNA isolation, a piece of leaf was incubated in 20 *µ*L of Extraction Solution (Sigma–Aldrich) at 95 °C for 10 min, followed by 5 min at 20 °C. Finally, 20 *µ*L of Dilution Solution (Sigma–Aldrich) supplemented with 1 mm MgCl_2_ (final concentration) was added, the samples were briefly vortexed, centrifuged at 16,100 × *g* for 30 s, and the supernatant containing the extracted gDNA was saved in a fresh microtube.

Total plant RNA from Arabidopsis leaf material was isolated with the NucleoSpin RNA Plant kit (Macherey-Nagel) and first-strand complementary DNA (cDNA) was synthesized with the SuperScript III First-Strand Synthesis System (Invitrogen) using 500 ng of RNA and oligo(dT)_18_ primer according to the manufacturer's protocols.

PCRs for genotyping were performed with the DreamTaq DNA polymerase (Thermo Scientific), while PCRs to generate fragments for sequencing or cloning were performed with the Phusion DNA polymerase (Thermo Scientific), with the primers listed in Supplementary Table S4.

For PCR genotyping, amplicons were separated by 1% (w/v) agarose gel electrophoresis with a GeneRuler 1 kb DNA Ladder (Thermo Scientific) as a size marker, and image acquisition was performed with the Quantum CX5-Epi UV imaging system (Vilber) and monitored with the Quantum CX5 software (version 17.06; Vilber).

For amplicon sequencing, DNA was isolated from the agarose gel using the NucleoSpin Gel and PCR Clean-up kit (Macherey-Nagel) according to the manufacturer's protocol. DNA sequences were obtained by Sanger sequencing (LGC Genomics, Berlin, Germany), and chromatogram visualization and in silico alignment of the obtained sequences with the WT *CEPA1* sequence from Araport11 were performed with the SeqMan Pro 17 software (version 17.2.0; DNASTAR Lasergene).

### EnsembleNet search

Candidate genes for PSI assembly were obtained using EnsembleNet (https://aranet.sbs.ntu.edu.sg/ensemblenet.html; Hansen et al. 2018). The loci for all five previously described nucleus-encoded assembly factors for PSI (*PYG7*, *Y3IP1*, *PPD1*, *PSA2*, and *PSA3*) were used as input in the “Gene set search” tool from EnsembleNet. The obtained list of candidate genes was curated by restricting it to nucleus-encoded genes, and candidates that cluster with all five assembly factors based on the Gene Ontology Jaccard Index (GOJI) (Hansen et al. 2018). The list of candidate genes was then filtered to include only genes coding for plastid-localized proteins. Finally, the list was further reduced by considering only proteins with uncharacterized function, putative transmembrane proteins and/or proteins with predicted protein–protein interaction domains in TAIR (https://www.arabidopsis.org/; Reiser et al. 2022), resulting in a final list of 22 candidate genes. The GOJIs of *CEPA1* and all five nuclear genes coding for PSI assembly factors were obtained by searching for the *CEPA1* gene in the EnsembleNet “Gene search” tool.

### Mutant complementation

Vector pDR016 harboring the *P_UBQ_:CEPA1-FLAG* cassette and the *pat* cassette (phosphinothricin resistance) for plant selection, flanked by the LB and RB T-DNA regions, and the *nptII* cassette (kanamycin resistance) for selection in bacteria, was constructed using an In-Fusion HD Cloning kit (Clontech) according to the manufacturer's protocol with minor modifications. The *CEPA1* coding sequence (CDS) without the stop codon was amplified by PCR from WT Arabidopsis cDNA as a template, including the backbone-flanking upstream region (15 bp) in the forward primer, and the FLAG epitope sequence, the stop codon and the backbone-flanking downstream region (15 bp) in the reverse primer (PCR 8 in Supplementary Table S4).The pVL15 backbone vector (harboring all the elements except the *CEPA1-FLAG* sequence; and generated previously; Loiacono et al. 2022) was linearized by double-digestion with the FastDigest EcoRI and Acc65I (Thermo Scientific) restriction enzymes. An insert: backbone ratio of 10:1 (∼40 ng of each) was mixed with 1 *µ*L of 5× In-Fusion HD Enzyme Premix (Clontech) in a 5 *µ*L In-Fusion reaction on ice. The cloning reaction was incubated at 50 °C for 20 min, and then placed on ice for 5 min.

A 1 *µ*L aliquot of the cloning reaction was used to transform *Escherichia coli* TOP10 (Invitrogen) competent cells. Transformed cells were selected on LB agar medium supplemented with 50 *µ*g mL^−1^ kanamycin overnight at 37 °C, and resistant colonies were confirmed to harbor pDR016 by colony PCR (PCR 7 in Supplementary Table S4). Finally, pDR016 was isolated from a bacterial culture of a positive colony using the NucleoSpin Plasmid kit (Macherey-Nagel) according to the manufacturer's protocol, and correctness of the sequence was confirmed by Sanger sequencing.

A volume equal to 200 ng of pDR016 was used to transform Agrobacterium (*A. tumefaciens*) GV3101 electrocompetent cells. Transformed cells were selected on YEB agar medium supplemented with 50 *µ*g mL^−1^ of gentamycin, 50 *µ*g mL^−1^ rifampicin, and 50 *µ*g mL^−1^ kanamycin at 28 °C for 2 d, and resistant colonies were confirmed to harbor the pDR016 vector by colony PCR. A positive colony was inoculated into fresh medium with the appropriate antibiotics.

Arabidopsis *cepa1-3* mutant plants were transformed with the pDR016-harboring Agrobacterium by the floral dip method according to Clough and Bent (1998). Selection of the complemented lines *cepa1-3-C1* and *C2* was done as described above.

### Thylakoid membrane isolation and subfractionation

Thylakoid membrane isolation was conducted according to Chen et al. (2016) and Järvi et al. (2011) with the following modifications. Arabidopsis rosettes were dark-adapted for 16 h and harvested at 4 °C under dim light. Detached leaves were ground in 10 mL of grinding buffer (50 mm HEPES/KOH, 1 mm MgCl_2_, 330 mm D-sorbitol, 2 mm EDTA, 5 mm sodium ascorbate, 0.05% (w/v) bovine serum albumin (BSA), pH 7.5) per gram of leaves with a pestle directly in a prechilled mortar. Homogenates were filtered through two layers of 20 *µ*m-pore nylon mesh and two layers of Miracloth (Merck Millipore), and the filtrate was centrifuged at 1,000 × *g* at 4 °C for 5 min. The pellet was resuspended in grinding buffer and centrifuged at 1,000 × *g* at 4 °C for 5 min. The pellet was resuspended in shock buffer (50 mm HEPES/KOH, 5 mm MgCl_2_, 5 mm D-sorbitol, pH 7.5) and centrifuged at 5,000 × *g* at 4 °C for 5 min. The pellet was then resuspended in storage buffer (50 mm HEPES/KOH, 10 mm MgCl_2_, 100 mm D-sorbitol, pH 7.5) and centrifuged at 5,000 × *g* at 4 °C for 5 min. Finally, the thylakoid membranes were resuspended in storage buffer.

For thylakoid subfractionation, freshly isolated thylakoid membranes from 4-wk-old WT plants were separated into grana cores, grana margins and stroma lamellae according to Puthiyaveetil et al. (2014) with the following modifications. Isolated thylakoids (equal to 1 mg chlorophyll) were centrifuged at 5,000 × *g* at 4 °C for 5 min, and resuspended in storage buffer to a final concentration of 0.5 mg chlorophyll mL^−1^. Thylakoid membranes were solubilized in a final concentration of 1% (v/v) digitonin (Serva) with gentle stirring in the dark on ice for 5 min, and centrifuged at 950 × *g* at 4 °C for 1 min. The supernatant containing the solubilized material was centrifuged at 40,000 × *g* at 4 °C for 30 min. The supernatant containing the nonappressed membranes was transferred to a fresh microtube, while the pellet containing the grana cores was resuspended in storage buffer and saved. The suspension of nonappressed membranes was centrifuged at 150,000 × *g* at 4 °C for 90 min, yielding a loose (grana margins) and a solid (stroma lamellae) pellet that were collected in storage buffer. The chlorophyll contents of total thylakoid membranes, grana core, grana margin, and stroma lamella isolations were measured in 80% (v/v) acetone (Porra et al. 1989) with the UV-Vis V-730 spectrophotometer (JASCO) run with the SpectraManager software (version 2.13.00; JASCO).

### Chloroplast isolation and fractionation

Chloroplasts were isolated from 4-wk-old WT leaves according to Armbruster et al. (2013) with the following modifications. All steps were performed in dim light at 4 °C. Leaves were ground in 10 mL of homogenization buffer (20 mm tricine/KOH, 450 mm D-sorbitol, 10 mm NaHCO_3_, 10 mm EDTA, 0.1% (w/v) BSA, pH 8.4) per g of fresh leaves in a blender. The homogenate was filtered through two layers of 20 *µ*m-pore nylon mesh and two layers of Miracloth (Merck Millipore), and the filtrate was centrifuged at 300 × *g* at 4 °C for 5 min. The resulting pellet was resuspended in 1.6 mL of 1× chloroplast resuspension (CR) buffer (100 mm tricine/KOH, 1,500 mm D-sorbitol, 25 mm MgCl_2_, 12.5 mm EDTA, pH 8.4) by gentle agitation. The chloroplast suspension was then loaded onto a 40/60/80% (v/v) Percoll (GE Healthcare) gradient employing a cut-off pipette tip, and centrifuged at 6,500 × *g* at 4 °C for 20 min. Intact chloroplasts were collected from the interphase between the 60% and 80% (v/v) Percoll phases, and transferred into two fresh microtubes. The chloroplast suspensions were diluted in three volumes of 1× CR buffer and centrifuged at 1,500 × *g* at 4 °C for 2 min. The pellet containing the intact chloroplasts from one tube was resuspended in 1.6 mL of 1× CR buffer supplemented with cOmplete EDTA-free Protease Inhibitor (Sigma–Aldrich; 1 tablet per 50 mL of 1× CR buffer) softly with a brush and saved. The chloroplasts from the second tube were lysed by resuspending the pellet in 1.6 mL of hypotonic buffer (50 mm HEPES/KOH, 5 mm MgCl_2_, protease inhibitor (1 tablet per 50 mL), pH 7.5), vortexing for 1 min and incubating on ice for 20 min. Disrupted chloroplasts were centrifuged at 16,100 × *g* at 4 °C for 30 min. The supernatant containing the stroma fraction was transferred into a fresh microtube, while the pellet containing the thylakoid membranes was resuspended in 1.6 mL of storage buffer and transferred into another fresh microtube. The chlorophyll contents of intact chloroplast and thylakoid membrane fractions were measured in 80% (v/v) acetone (Porra et al. 1989). The protein content in the stromal fraction was quantified by the BCA assay (Pierce BCA Protein Assay, Thermo Scientific) according to the manufacturer's protocol.

### Photosynthetic measurements

The preliminary screening by measurement of the qL LRC on 10-d-old seedlings (five individuals per line), referred to as “qL screening”, was done with the IMAGING-PAM *M-Series* (MAXI version; Walz) using the ImagingWin software (version 2.56; Walz). Seedlings were dark-adapted for 30 min prior to the measurement. The light intensity was increased in 20 steps from 0 to 800 *µ*mol photons m^−2^ s^−1^, with intervals of 150 s from 0 to 30 *µ*mol photons m^−2^ s^−1^, 120 s until 60 *µ*mol photons m^−2^ s^−1^, 90 s until 110 *µ*mol photons m^−2^ s^−1^, and 60 s until 800 *µ*mol photons m^−2^ s^−1^.

LRCs of chlorophyll-*a* fluorescence parameters in 4- to 5-wk-old plants were determined with the fiberoptics version of the DUAL-PAM-100 (Walz). Leaves were dark-adapted for 30 min prior to the measurement. Then, under light-limited conditions, the light intensity was increased in 150 s intervals. Under light-saturated conditions above 500 *µ*mol photons m^−2^ s^−1^, the light intensity was increased every 60 s. PSI-related measurements were performed with the plastocyanin-P_700_ version of the Dual-PAM instrument (Schöttler et al. 2007). Plants were directly taken from the growth chambers, and leaves were preilluminated at growth light intensity for 3 min prior to measurements, to activate the Calvin–Benson–Bassham cycle and thereby avoid an acceptor-side limitation of PSI. After 10 s in the dark, the maximum difference absorbance signals of redox-active plastocyanin and PSI were determined by far-red illumination for 8 s, followed by a short saturating light pulse. The light intensity was then stepwise increased as described above for the chlorophyll-*a* fluorescence measurements. The fractions of PSI reaction centers limited at their donor side, Y(ND), and acceptor side, Y(NA), were determined according to published procedures (Schreiber and Klughammer 2016). After the LRCs had been completed, the chlorophyll content of the measured leaf section was determined in 80% (v/v) acetone (Porra et al. 1989).

PSII, cytochrome *b*_6_*f* complex, and PSI were quantified spectroscopically in thylakoids isolated as described previously (Schöttler et al. 2004). PSII and cytochrome *b*_6_*f* complex contents were determined from difference absorbance signals of cytochromes *b*_559_ (PSII), *f*, and *b*_6_ (cytochrome *b*_6_*f* complex) measured with the V-750 spectrophotometer equipped with a head-on photomultiplier (JASCO). After complete oxidation of all cytochromes by the addition of 1 mm potassium ferricyanide(III), cytochrome *f*, and the high-potential form of cytochrome *b*_559_ were reduced by the addition of 5 mm sodium ascorbate, followed by the addition of 10 mm sodium dithionite to reduce the low-potential forms of cytochrome *b*_559_ and cytochrome *b*_6_. Difference absorbance spectra were calculated, baseline-corrected and deconvolved according to published procedures (Kirchhoff et al. 2002; Lamkemeyer et al. 2006). The PSI content was determined from difference absorbance signals of P_700_ as described previously (Schöttler et al. 2007). Plastocyanin contents, relative to PSI, were determined in leaves by in vivo difference absorption spectroscopy in the far-red range of the spectrum and then recalculated based on the absolute PSI quantification in isolated thylakoids (Schöttler et al. 2007). Finally, all complex contents were re-normalized to a leaf area basis with the known chlorophyll contents per leaf area.

77K chlorophyll-*a* fluorescence emission spectra were measured using the F-8300 spectrofluorometer (JASCO) on freshly isolated thylakoids equivalent to 10 *μ*g chlorophyll mL^−1^. The sample was excited at 430 nm wavelength with a bandwidth of 10 nm, and the emission spectrum was recorded between 650 and 800 nm wavelength in 0.5 nm intervals with a bandwidth of 1 nm.

For statistics of photosynthetic measurements, ANOVA analyses using an “All Pairwise Multiple Comparison Procedure (Holm-Sidak method)” were performed using SigmaPlot (version 14.5; Systat).

### RNA and ribosome footprint profiling

RNA and ribosome footprint profiling was performed using 4-wk-old WT and *cepa1-3* rosette leaves for RNA extraction and ribosome footprint isolation. The analyses were performed as described previously (Trösch et al. 2018; Schuster et al. 2020).

### Trypsin protection assay

Freshly isolated thylakoid membranes (equal to 5 *µ*g chlorophyll) were resuspended in storage buffer at 0.1 *µ*g chlorophyll *µ*L^−1^, then supplemented with a final concentration of 0, 1, or 5 *µ*g mL^−1^ trypsin. Samples were incubated at 37 °C for 30 min. An equal volume of 4× Laemmli buffer (200 mm Tris/HCl, 40% (v/v) glycerol, 8% (w/v) SDS, 200 mm dithiothreitol (DTT), 0.004% (w/v) bromophenol blue, pH 6.8) was added to the samples, followed by boiling at 95 °C for 5 min and centrifugation at 16,100 × *g* for 5 min. Samples of the supernatant (equal to 1 *µ*g chlorophyll) were separated by SDS-PAGE and subjected to immunoblotting.

### Polyacrylamide gel electrophoresis

SDS-polyacrylamide gel electrophoresis (SDS-PAGE) was conducted according to Laemmli (1970). Isolated thylakoids, isolated chloroplasts, fractionated chloroplasts, and subfractionated thylakoids were mixed with an equal volume of 4× Laemmli buffer at 0.1 *µ*g chlorophyll *µ*L^−1^ (or 1 *µ*g protein *µ*L^−1^ for stromal fraction). Co-IP eluates were mixed with DTT to a final concentration of 50 mm, while 20 *µ*L of NBF were mixed with Pierce Lane Marker Non-Reducing Sample Buffer (Thermo Scientific) to a final concentration of 1× and DTT to a final concentration of 50 mm. The samples were then incubated at 40 °C for 30 min and centrifuged at 16,100 × *g* at room temperature for 5 min. The supernatants were loaded on a 15% resolving/4% stacking SDS-polyacrylamide gel. The PageRuler Plus Prestained Protein Ladder (Thermo Scientific) was loaded as a molecular weight marker. Electrophoresis was performed in 1× Tris–Glycine–SDS (TGS) buffer (25 mm Tris, 192 mm glycine, 0.1% (w/v) SDS, pH 8.3) at room temperature at an initial voltage of 25 V until the blue dye front enters the resolving gel and the voltage was then increased to 100 V until satisfying separation was obtained based on the ladder profile and the blue dye migration.

For in-gel trypsin digestion, co-IP eluate samples (20 *µ*L) were supplemented with DTT to a final concentration of 50 mm, incubated at room temperature for 15 min, centrifuged at 16,100 × *g* for 5 min, and the supernatant was loaded on a 12% resolving/4% stacking SDS-polyacrylamide gel, followed by short electrophoretic separation.

Blue-native PAGE (BN-PAGE) and large pore BN-PAGE were conducted according to Järvi et al. (2011). Briefly, thylakoid membranes were centrifuged at 5,000 × *g* at 4 °C for 5 min, and thylakoid pellets were resuspended in 25BTH20G buffer (25 mm Bis-Tris/HCl, 20% (v/v) glycerol, 1× protease inhibitor (1 tablet per 50 mL), pH 7.0). Thylakoid membranes (at a final concentration of 0.5 *µ*g chlorophyll *µ*L^−1^) were solubilized in a final concentration of 1% (w/v) β-DM (Roth) in the dark on ice for 15 min, or 1.5% (w/v) digitonin (Serva) on a rotating wheel in the dark at 4 °C for 1 h. The β-DM or digitonin-solubilized samples were centrifuged at 16,100 × *g* at 4 °C for 30 min and 1 h, respectively, and the upper half of the supernatant was transferred to a fresh microtube and mixed with a final concentration of 1× Serva blue G loading buffer (10 mm Bis-Tris/HCl, 50 mm 6-aminocaproic acid, 3% (w/v) sucrose, 0.5% (w/v) Coomassie G powder, pH 7.0). The samples were then loaded on a 5% to 12.5% resolving gradient/4% stacking BN-polyacrylamide gel. The anode section was filled with 50 mm Bis-Tris/HCl (pH 7.0), while the cathode section was filled with blue cathode buffer (15 mm Bis-Tris/HCl, 50 mm tricine, pH 7.0, supplemented with a final concentration of 0.01% (w/v) Coomassie Brilliant Blue G250 (Serva)) for the first half of the run, and in clear cathode buffer (without Coomassie) for the second half. Electrophoresis was performed at 4 °C as follows: at 75 V for 30 min, 100 V for 30 min, 125 V for 30 min, 150 V for 1 h, 175 V for 30 min, and 200 V until the blue migration front had reached the bottom of the gel.

For second-dimension 2D BN/SDS-PAGE, lanes of interest from the first-dimension BN-PAGE were excised and incubated in clear 1× Laemmli buffer (without bromophenol blue) with mild agitation at room temperature for 1 h. Two gel lanes were placed horizontally next to each other, on top of a 15% resolving/4% stacking SDS-polyacrylamide gel. Pieces of Whatman paper wetted with the PageRuler Plus Prestained Protein Ladder (Thermo Scientific) were placed between the two gel lanes, and the setup was fixed with 0.1% (w/v) agarose. Electrophoresis was performed similar to one-dimensional SDS-PAGE.

### Coomassie staining and immunoblotting

Proteins separated by 1D or 2D SDS-PAGE were transferred to polyvinylidene difluoride (PVDF) membranes in Towbin buffer (25 mm Tris, 192 mm glycine, pH 8.3) supplemented with 20% (v/v) methanol at 4 °C and 400 mA for 90 min with stirring. Membranes subjected to Coomassie staining were incubated in staining solution (40% (v/v) methanol, 7% (v/v) acetic acid, 0.1% (w/v) Coomassie Brilliant Blue R250 (Serva)) for 5 min. The stained membranes were washed in destaining solution (staining solution without Coomassie) at least three times for 5 min each, until the signal-to-background intensity was satisfying. Finally, membranes were rinsed in distilled water two times for 5 min each, air dried and scanned. Membranes subjected to immunoblot assays were incubated in Tris-buffered saline (TBS; 20 mm Tris/HCl, 150 mm NaCl, pH 7.6) supplemented with 0.1% (v/v) Tween 20 (TBS-T) two times for 5 min each. The membranes were then blocked in TBS-T supplemented with 2% (w/v) milk for 30 min, and washed four times for 5 min each. The membranes were incubated with appropriate primary antibody dilutions (Supplementary Table S5) for 2 h, and washed four times for 5 min each. Finally, membranes were incubated with secondary antibody dilutions (Supplementary Table S5) for 1 h, and washed four times for 5 min each. All washes and incubations were performed at room temperature on a shaker with mild agitation.

Horseradish peroxidase-coupled secondary antibodies were detected by chemiluminescence assays using the ECL Prime Western Blotting Detection Reagents A:B (1:1, GE Healthcare), and image acquisition was performed with the ChemiDoc MP imaging system (Bio-Rad) with the Image Lab software (version 5.2.1; Bio-Rad).

For 1D BN-PAGE, gels were incubated in 1× clear Laemmli buffer (without bromophenol blue) at room temperature for 1 h with mild agitation, and proteins were transferred to PVDF membranes as described above. Membranes were washed in distilled water two times for 5 min each, air dried and scanned for use as a profile reference for the subsequent immunoblots. The green and blue stains resulting from the presence of chlorophylls in complexes and Serva blue G binding, respectively, were removed by incubating the membranes in 100% methanol at least two times for 10 min each (until most of the color had disappear), and membranes were rehydrated in distilled water two times for 5 min each. Finally, the membranes were subjected to immunoblotting as described above.

### Antibodies

Antibodies used in this study are listed in Supplementary Table S5. Antibodies against CEPA1 from Arabidopsis were produced by BioGenes (Berlin, Germany), using the CEPA1 peptide sequence C-LLDVLKESDKKSKRK based on its immunogenic potential (manufacturer's prediction) and specificity in the Arabidopsis proteome (BLASTp search). The antigenic peptides were synthesized with an additional N-terminal cysteine for conjugation with the *Limulus polyphemus* hemocyanin (LPH) carrier. Two rabbits were immunized with the conjugated peptide for several boosts. The antisera were tested by immunoblotting using thylakoid membrane proteins from the WT and the *cepa1-3* null mutant (negative control), until satisfying detection of the protein of interest had been achieved. Finally, the antiserum of the chosen rabbit was purified with the antigenic peptide bound to a column to reduce background signals. The final stock of purified antibodies against CEPA1 (0.3 *µ*g *µ*L^−1^) was stored in antibody storage buffer (25 mm Tris, 192 glycine, 250 mm NaCl, 0.1% (v/v) ProClin 300 (Sigma–Aldrich), pH 7.5), and was used to prepare the antibody dilution (Supplementary Table S5).

### Chilling, etiolation, and lincomycin treatments

For all treatments, seeds were surface sterilized with a solution of 1% (w/v) sodium hypochlorite, washed with sterile water, sown on 0.5× MS agar medium supplemented with 1% (w/v) sucrose, and stratified for 2 d.

For chilling stress experiments, plates were placed for 5 d under standard conditions (16 h light/8 h dark cycle; light intensity: 100 *µ*mol photons m^−2^ s^−1^, temperature: 22 °C), and then transferred to the low-temperature condition (16 h light/8 h dark cycle; light intensity: 100 *µ*mol photons m^−2^ s^−1^, temperature: 12 °C during the day and 10 °C during the night). After 22 d of cultivation under cold conditions, the samples were harvested for phenotyping, qL screening and thylakoid membrane isolation as described above.

Etiolation experiments were conducted according to Sandoval-Ibáñez et al. (2021). The plates were exposed to light (95 *μ*mol photons m^−2^ s^−1^) for 1 h, covered with two layers of aluminum foil (for etiolation), and cultivated for 5 d (temperature: 22 °C). Samples were then harvested under nonphotosynthetic light (green light). The samples were ground in liquid nitrogen, and the membranes were extracted employing the same method as described above for thylakoid isolation. For BN-PAGE, the samples were solubilized in 1% (w/v) β-DM and resolved in a 8% to 13.8% resolving gradient gel as reported by Sandoval-Ibáñez et al. (2022).

For lincomycin treatment, plates were placed for 12 d under standard conditions. Plants were then collected and infiltrated twice in a solution containing water or 1 mm lincomycin for 20 min. The plants were then incubated at 4 °C overnight, and transferred to low light (5 *μ*mol photons m^−2^ s^−1^) for 0, 2 or 4 d. For each time-point, material was harvested, ground in liquid nitrogen, and thylakoid membranes were isolated as described above.

### CEPA1-FLAG co-IP

The CEPA1-FLAG co-IP was performed with three independent isolations of *cepa1-3-C1* and WT thylakoid membranes using Anti-FLAG M2 Magnetic Beads (Sigma–Aldrich). The beads (40 *µ*L per replicate) were transferred to a fresh microtube, and washed in 400 *µ*L of 25BTH20G buffer on a rotating wheel two times at room temperature for 5 min. A third wash was done in 400 *µ*L 25BTH20G supplemented with 0.1% (w/v) β-DM on a rotating wheel at room temperature for 5 min.

Thylakoid membranes isolated from 4-wk-old *cepa1-3-C1* and WT plants growing in short-day conditions were harvested (three independent isolations per line). Thylakoid membranes (equal to 120 *µ*g chlorophyll) were transferred to fresh microtubes, centrifuged at 5,000 × *g* at 4 °C for 5 min, and thylakoid pellets were resuspended in ice-cold 25BTH20G buffer. Thylakoid membranes (0.1 *µ*g chlorophyll *µ*L^−1^) were solubilized in a final concentration of 1% (w/v) β-DM in the dark on ice for 15 min. Solubilized membranes were centrifuged at 16,100 × *g* at 4 °C for 30 min, and the upper part of the supernatant (equal to 100 *µ*g chlorophyll) was transferred to the bead-containing tube. The co-IP samples were then incubated on a rotating wheel at 4 °C for 2 h. The supernatant containing the NBF was transferred to a fresh microtube. The beads were then washed three times in 1 mL of 25BTH20G supplemented with 0.1% (w/v) β-DM on a rotating wheel at 4 °C for 5 min each, followed by three additional washes in the same conditions without β-DM. Proteins were eluted two times by incubating the beads in 50 *µ*L of 1× Pierce Lane Marker Non-Reducing Sample Buffer (Thermo Scientific) at 95 °C for 10 min. The two eluates were combined into a fresh microtube (100 *µ*L eluate sample). The NBF and the eluted samples were further analyzed by immunoblot assays and LC-MS/MS.

### LC-MS/MS analysis of co-IP samples

Proteins eluted from co-IP samples were separated by SDS-PAGE and subjected to in-gel trypsin digestion according to Shevchenko et al. (2006). Briefly, proteins in the gel slices were incubated in destaining solution (50% (v/v) acetonitrile (ACN), 50 mm NH_4_HCO_3_) in 30 min washes with shaking at 1,200 × *g* (until no visible staining remained), followed by incubation in reduction solution (10 mm DTT, 50 mm NH_4_HCO_3_) at 56 °C for 30 min and incubation in alkylation solution (55 mm iodoacetamide, 50 mm NH_4_HCO_3_) in the dark for 30 min, and finally digested in trypsin solution (10 *μ*g mL^−1^ Trypsin/LysC (Promega), 0.001% (v/v) trifluoroacetic acid (TFA), 50 mm NH_4_HCO_3_) at 37 °C overnight, with dehydration in 100% ACN between each step. Tryptic peptides were extracted from the gel pieces, first in 30% (v/v) ACN, 1% (v/v) TFA, and subsequently in 100% ACN. The extracts were combined in a fresh tube and dried in a vacuum centrifuge (Concentrator Plus, Eppendorf). Near-dry tryptic peptides were dissolved in 0.1% (v/v) TFA and desalted with C_18_ ZipTip columns (Merck Millipore). Desalted peptides were dried in the vacuum centrifuge and resuspended in 50 *µ*L of 4% (v/v) ACN and 0.1% (v/v) formic acid (FA).

Tryptic peptide samples were processed by liquid chromatography coupled to tandem mass spectrometry (LC-MS/MS), employing the ACQUITY M-Class system (Waters) ultra performance LC (UPLC) with the ACQUITY Console software (version 1.74.2662; Waters), and the Q Exactive Plus (Thermo Scientific) mass spectrometer with the Exactive Series Tune software (version 2.11 QF1 Build 3006; Thermo Scientific). Methods and injection sequences were configured with the Xcalibur software (version 4.4.16.14; Thermo Scientific). A seesaw cycle was run between each sample to limit the carryover. For UPLC, the two solvents A and B were 0.1% (v/v) FA in water and 0.1% FA (v/v) in ACN, respectively. In each run, 3 *µ*L of peptide sample was injected into the UPLC system. The peptides were first trapped in a nanoEase M/Z C_18_ trap column (Waters) with a mobile phase of 4% solvent B at a flow rate of 10 *µ*L min^−1^ for 1.5 min. Peptides were then separated on a nanoEase M/Z C_18_ analytical column (Waters) at a flow rate of 300 nL min^−1^ with a linear gradient of 4% to 35% solvent B for 30 min, 35% to 80% solvent B for 2 min, 80% to 96% solvent B for 1 min, and constant 96% solvent B for 3 min, before columns were re-equilibrated in 4% solvent B for 15 min. Tryptic peptides were ionized by a nano electrospray source (Nanospray Flex Ion Source, Thermo Scientific) and analyzed in the mass spectrometer in positive ion mode. The precursor ions were selected in the quadrupole in the 200 to 2,000 *m*/*z* range at a resolution of 70,000 (at *m*/*z* 200), and precursors ions with single or ≥5 charge states were excluded. Precursor ions were fragmented in the high-energy collisional dissociation (HCD) cell with an automatic gain control (AGC) target value of 100,000 ions. Product ions were analyzed in the Orbitrap with a resolution of 17,500 (at *m*/*z* 200), and MS/MS scans were acquired using a data-dependent top15 method.

Protein identification and quantification from MS/MS spectra was performed with MaxQuant (version 1.6.0.13; Cox and Mann 2008). Tryptic peptides were searched against a database including the Araport11 protein list (Cheng et al. 2017) supplemented with the CEPA1-FLAG sequence and a list of common contaminants (e.g. trypsin, BSA, keratin). In the search parameters, Trypsin/P was set as the protease with a maximum of two missed cleavages. The carbamidomethylation of cysteine was set as fixed modification, while methionine oxidation and protein N-terminal acetylation were set as variable modifications, with up to five modifications per peptide. The label-free quantification “LFQ” (corresponding to the MaxLFQ algorithm in MaxQuant; Cox et al. 2014) was set as quantification method, the minimum ratio count was set to 1, and the “Match between runs” option was activated. All other parameters were set as default. In the output, low-confidence proteins were removed as described in Beer et al. (2017).

The Perseus software (version 1.5.8.5; Tyanova et al. 2016) was used for statistical analysis of the enrichment of proteins in the CEPA1-FLAG IP (*cepa1-3-C1* IP against WT IP), with the help of the guidelines available in the Perseus online documentation (http://www.coxdocs.org/doku.php? id = perseus:user:use_cases:interactions&s[]=s0). LFQ intensity values were transformed with a log_2_(LFQ intensity value) function. Proteins only identified by site and reverse sequences were filtered out. All three replicates of each line were assigned to their corresponding group (i.e. “*cepa1-3-C1* IP” and “WT IP” groups). All proteins with a valid intensity value (log_2_(intensity value) ≠ NaN, i.e. intensity value > 0) in all three replicates of at least one group were selected. Among the selected proteins, missing intensity values in appropriate replicates were subsequently imputed from normal distribution of the total matrix with default parameters (width = 0.3, down shift = 1.8). The resulting final data set was analyzed to compare protein abundances in the *cepa1-3-C1* IP versus the WT IP. Proteins were considered as significantly enriched when they were at least 2-fold more abundant in the *cepa1-3-C1* IP than in the WT IP (i.e. log_2_(*cepa1-3-C1* IP/WT IP ≥ 1) with a *P*-value < 0.05 (two-sample Student's *t*-test).

### BiFC assay

Vectors were prepared following the MoBiFC protocol, based on the Golden Gate method, with some modifications (Velay et al. 2022). MoBiFC vectors obtained from Addgene (Weber et al. 2011; Engler et al. 2014; Velay et al. 2022) or generated in this study are listed in Supplementary Table S6. The CDSs of PsaC and the mature CEPA1 (without the stop codon) were amplified from Arabidopsis cDNA using primers that included a BbsI recognition site on each side of the sequence (PCRs 9 and 10 in Supplementary Table S4). The CDSs of mature PPD1 and PSA3 (without the stop codon) were chemically synthesized (Thermo) with silent mutations to remove internal BbsI or BsaI sites, and with a BbsI site on each side of the sequence.

Level 0 modules were cloned by mixing 50 fmol of CDS inserts with 25 fmol of pAGM1299 backbone in a 10 *µ*L cloning reaction (200 CEU of T4 DNA ligase (Thermo Scientific), 1× T4 DNA ligase buffer (Thermo Scientific)) supplemented with 6 U of BbsI-HF (NEB), and incubated in a thermocycler with the following program: 37 °C for 10 min, 30 cycles of 37 °C for 1 min, and 16 °C for 1 min, 16 °C for 10 min, 65 °C for 10 min. Samples were collected by a short spin, and cooled on ice. A 1 *µ*L aliquot was used to transform *E. coli* competent cells, and positive colonies (spectinomycin-resistant white colonies) were selected on LB agar supplemented with 100 *µ*g mL^−1^ spectinomycin, 20 *µ*g mL^−1^ X-Gal and 100 *µ*M IPTG (blue/white assay), followed by plasmid isolation.

Level 1 transcription units were cloned by mixing 50 fmol of level 0 modules with 25 fmol of pICH47742 (for nYFP fusions) or pICH47751 (for cYFP fusions) backbone, as indicated in Supplementary Table S7, in a 10 *µ*L cloning reaction supplemented with 6 U of BsaI-HFv2 (NEB), and incubated as described above. A 1 *µ*L aliquot was used to transform *E. coli* competent cells, and positive colonies (carbenicillin-resistant white colonies) were selected on LB agar supplemented with 100 *µ*g mL^−1^ carbenicillin, 20 *µ*g mL^−1^ X-Gal and 100 *µ*M IPTG (blue/white assay), followed by plasmid isolation.

Level 2 multigene units were cloned by mixing 50 fmol of appropriate level 1 transcription units with 25 fmol of the pAGM4673 backbone in a 10 *µ*L cloning reaction supplemented with 6 U of BbsI-HF, and incubated as described above. A 1 *µ*L aliquot was used to transform *E. coli* competent cells, and positive colonies (kanamycin-resistant white colonies) were selected in LB agar medium supplemented with 50 *µ*g mL^−1^ kanamycin (red/white assay), followed by plasmid isolation.

Agrobacterium GV3101 electrocompetent cells were transformed with 200 ng of level 2 multigene units, and selected on YEB agar medium supplemented with 50 *µ*g mL^−1^ of gentamycin, 50 *µ*g mL^−1^ rifampicin, and 50 *µ*g mL^−1^ kanamycin. Positive colonies were propagated in liquid YEB with appropriate antibiotics. The cultures were washed three times in distilled water, and the bacteria were resuspended in MMA solution (10 mm MES, 10 mm MgCl_2_, 0.1 mm acetosyringone, pH 5.6) at OD_600_ = 0.5. Individual leaves of 5-wk-old *N. benthamiana* plants were infiltrated with the Agrobacterium suspensions.

Protoplasts were prepared 3 d after infiltration by incubating pieces of transformed leaves in enzyme solution (20 mm MES, 20 mm KCl, 10 mm CaCl_2_, 400 mm mannitol, 0.1% (w/v) BSA, 1% (w/v) cellulase, 0.25% (w/v) macerozyme, pH 5.7) in the dark with gentle agitation for 1 h. The protoplast suspensions were centrifuged at 200 × *g* for 1 min, most of the supernatant was discarded, and the loose protoplast pellet was gently resuspended in the remaining supernatant.

Protoplast suspensions were assayed for fluorescence by confocal laser-scanning microscopy. The mTRQ (mTurquoise2 (FPbase ID: 7AV5G); excitation/emission: 440/460 to 500 nm), YFP (EYFP (FPbase ID: 8DNLG); excitation/emission: 514/524 to 554 nm), and chlorophyll (excitation/emission: 405/650 to 720 nm) fluorescence signals, together with the bright-field image, were acquired with a STELLARIS 8 multiphoton Microscope (Leica) using the Leica Application Suite X software (version 3.7.1.21655; Leica). Identical laser intensity and gain values were employed to capture all images. Images were merged and mounted with Fiji (version 1.54f; Schindelin et al. 2012).

### Assessment of protein features and evolutionary conservation

Subcellular protein localization was assessed using SUBA4 (https://suba.live/; Hooper et al. 2017). The cTP cleavage site of CEPA1 was predicted in Arabidopsis with TargetP-2.0 (https://services.healthtech.dtu.dk/service.php? TargetP-2.0; Almagro Armenteros et al. 2019) and in Chlamydomonas (*C. reinhardtii* with PredAlgo (http://lobosphaera.ibpc.fr/predalgo; Tardif et al. 2012). The transmembrane domain was predicted with DeepTMHMM (https://dtu.biolib.com/DeepTMHMM; Hallgren et al. 2022). The three-dimensional structure of CEPA1 was predicted by AlphaFold (https://alphafold.ebi.ac.uk/; Jumper et al. 2021; Varadi et al. 2022). CEPA1-specific peptides from proteomic datasets were retrieved from the PPDB database (http://ppdb.tc.cornell.edu/; Sun et al. 2009) and the Arabidopsis PeptideAtlas (build 2021-03; http://www.peptideatlas.org/builds/arabidopsis/; van Wijk et al. 2021). The *CEPA1* co-expression network was extracted from ATTED-II (version 11.1; https://atted.jp/; Obayashi et al. 2022). The structure of PSI–LHCI (PDB ID: 5L8R) was retrieved from the RCSB PDB website (https://www.rcsb.org/; Berman et al. 2000).

CEPA1 protein conservation in other organisms was assessed using the BLASTp service from the National Center for Biotechnology Information (NCBI; website version of 02.02.2022; https://blast.ncbi.nlm.nih.gov/Blast.cgi?PAGE=Proteins; Boratyn et al. 2013). The AtCEPA1 protein sequence from Arabidopsis was used as input for the query including all organisms in the search database and employing default search parameters. The CbCEPA1 protein sequence from *C. braunii* was used as input in two new queries with default search parameters, and (i) including only, or (ii) excluding land plants in the search database (NCBI taxonomy ID (taxid): 3193). The OlCEPA1 protein sequence from *O. lucimarinus* was used as input in a new query with default search parameters. The CrCEPA1 protein sequence from Chlamydomonas was used as input in a final query with default parameters including only the cyanobacteria search database (taxid: 1117).

The putative homolog of CEPA1 in Chlamydomonas was further assessed using HHpred (https://toolkit.tuebingen.mpg.de/tools/hhpred; Zimmermann et al. 2018) and the AtCEPA1 sequence as a query, selecting “Euk_Chlamydomonas_reinhardtii_27_Jul_2017” as the proteome reference and setting all other parameters as default.

Amino acid sequence alignments were produced using Clustal Omega (https://www.ebi.ac.uk/Tools/msa/clustalo/; Sievers et al. 2011) from the European Molecular Biology Laboratory's European Bioinformatics Institute platform (EMBL-EBI; Madeira et al. 2022). Alignment visualizations were generated using Jalview (version 2.11.1; Waterhouse et al. 2009), with amino acids highlighted according to the ClustalX standard color scheme (Thompson et al. 1997).

Information about mutants for the CEPA1 homolog in Chlamydomonas were retrieved from the CLiP (https://www.chlamylibrary.org/index; Li et al. 2019) and Chlamydomonas Resource Center websites (https://www.chlamycollection.org/).

### Statistical analysis

Statistical data are provided in Supplementary Data Set 2.

### Accession numbers

Nucleotide sequence data for genes relevant to this article can be found in the TAIR and Phytozome databases (https://phytozome-next.jgi.doe.gov/; Goodstein et al. 2012) under the accession numbers listed in Supplementary Table S8. Amino acid sequences of putative CEPA1 homologs can be found in the NCBI (https://www.ncbi.nlm.nih.gov/protein/; Sayers et al. 2022) and UniProt databases (https://www.uniprot.org/; The UniProt Consortium 2021) under the following accession numbers: AtCEPA1 (NP_191160.1/Q9LY44; Arabidopsis), CbCEPA1 (GBG62996.1; *Chara braunii*), CrCEPA1/LGS1 (XP_001689895.2; Chlamydomonas), KnCEPA1 (GAQ80375.1; *K. nitens*), NtCEPA1 (XP_016456372.1; *N. tabacum*), OsCEPA1 (XP_015632728.1; *O. sativa*), OlCEPA1 (XP_001416794.1; *O. lucimarinus*), PpCEPA1 (XP_024386960.1; *P. patens*) and ZmCEPA1 (NP_001168569.1; *Z. mays*).

## Supplementary Material

koae042_Supplementary_Data

## Data Availability

The data underlying this article are available in the article and in its online supplementary material, and will be shared on reasonable request to the corresponding author.

## Supplementary data

The following materials are available in the online version of this article.

**Supplementary Figure S1.** Properties of the *CEPA1* gene in *A. thaliana*.

**Supplementary Figure S2.** Sequencing of the left borders of the T-DNA insertion sites in the *cepa1-1*, *cepa1-2*, and *cepa1-3* mutant lines.

**Supplementary Figure S3.** Selection of the complemented lines.

**Supplementary Figure S4.** Physical map of Cre01.g014000/*CrCEPA1*/*LGS1* and marker cassette insertion sites in CLiP mutants.

**Supplementary Figure S5.** CEPA1 and PSI subunit accumulation after lincomycin treatment.

**Supplementary Figure S6.** Phenotyping of *cepa1* mutants grown in the cold.

**Supplementary Figure S7.** Change of plastid RNA and ribosome footprint levels, and resulting translation efficiencies in *cepa1-3* in comparison to the WT.

**Supplementary Figure S8.** CEPA1 does not associate with high-molecular weight complexes in the absence of PSI biogenesis.

**Supplementary Figure S9.** The mature CEPA1-FLAG protein and CEPA1 fragments are pulled down by co-IP.

**Supplementary Figure S10.** *CEPA1* co-expression network.

**Supplementary Figure S11.** Representative BiFC assay with nYFP fused to the C-terminus of CEPA1.

**Supplementary Table S1.** CEPA short-list of candidates obtained from EnsembleNet.

**Supplementary Table S2.** Sequences of the CEPA1 gene and protein in *A. thaliana*.

**Supplementary Table S3.** Details of CLiP strains harboring a mutation in the *CrCEPA1* gene.

**Supplementary Table S4.** List of primers used in this study.

**Supplementary Table S5.** List of antibodies used in this study.

**Supplementary Table S6.** List of vectors used in MoBiFC.

**Supplementary Table S7.** Cloning of MoBiFC vectors level 1 transcription units.

**Supplementary Table S8.** List of accession numbers for the genes relevant to this study.

**Supplementary Data Set 1.** Data from array-based ribosome profiling experiments of *cepa1-3* and the WT.

**Supplementary Data Set 2.** Statistical tests.

## Dive Curated Terms

The following phenotypic, genotypic, and functional terms are of significance to the work described in this paper:

LHCA4 Gramene: AT3G47470
LHCA4 Araport: AT3G47470

PSAF Gramene: AT1G31330
PSAF Araport: AT1G31330

PSAG Gramene: AT1G55670
PSAG Araport: AT1G55670

PSAN Gramene: AT5G64040
PSAN Araport: AT5G64040

PSAO Gramene: AT1G08380
PSAO Araport: AT1G08380

PSA3 Gramene: AT3G55250
PSA3 Araport: AT3G55250

PYG7 Gramene: AT1G22700
PYG7 Araport: AT1G22700

PPD1 Gramene: AT4G15510
PPD1 Araport: AT4G15510

Y3IP1 Gramene: AT5G44650
Y3IP1 Araport: AT5G44650

CEPA1 Gramene: AT3G56010
CEPA1 Araport: AT3G56010

PIR1 Gramene: Cre01.g014000
PIR1 Araport: Cre01.g014000

PSAK Gramene: AT1G30380
PSAK Araport: AT1G30380

PSAL Gramene: AT4G12800
PSAL Araport: AT4G12800

LHCA1 Gramene: AT3G54890
LHCA1 Araport: AT3G54890

LHCA2 Gramene: AT3G61470
LHCA2 Araport: AT3G61470

LHCA3 Gramene: AT1G61520
LHCA3 Araport: AT1G61520

PsaA Gramene: ATCG00350
PsaA Araport: ATCG00350

PsaB Gramene: ATCG00340
PsaB Araport: ATCG00340

PsaC Gramene: ATCG01060
PsaC Araport: ATCG01060

PSAD Gramene: AT4G02770
PSAD Araport: AT4G02770

PSAE Gramene: AT4G28750
PSAE Araport: AT4G28750

PSAH Gramene: AT3G16140
PSAH Araport: AT3G16140

PsaI Gramene: ATCG00510
PsaI Araport: ATCG00510

PsaJ Gramene: ATCG00630
PsaJ Araport: ATCG00630

Ycf3 Gramene: ATCG00360
Ycf3 Araport: ATCG00360

Ycf4 Gramene: ATCG00520
Ycf4 Araport: ATCG00520
