# Supplementary material for: CO-EXPRESSED WITH PSI ASSEMBLY1 (CEPA1) is a photosystem I assembly factor in Arabidopsis
Source: Plant Cell. 2024 Feb 21;36(10):4179–211. doi: 10.1093/plcell/koae042 (PMC11449006; doi:10.1093/plcell/koae042)
Supplement: koae042_Supplementary_Data [file koae042_supplementary_data.zip › TPC2023-RA-00879R1_Supplementary_Figures_and_Tables.pdf]

**A**

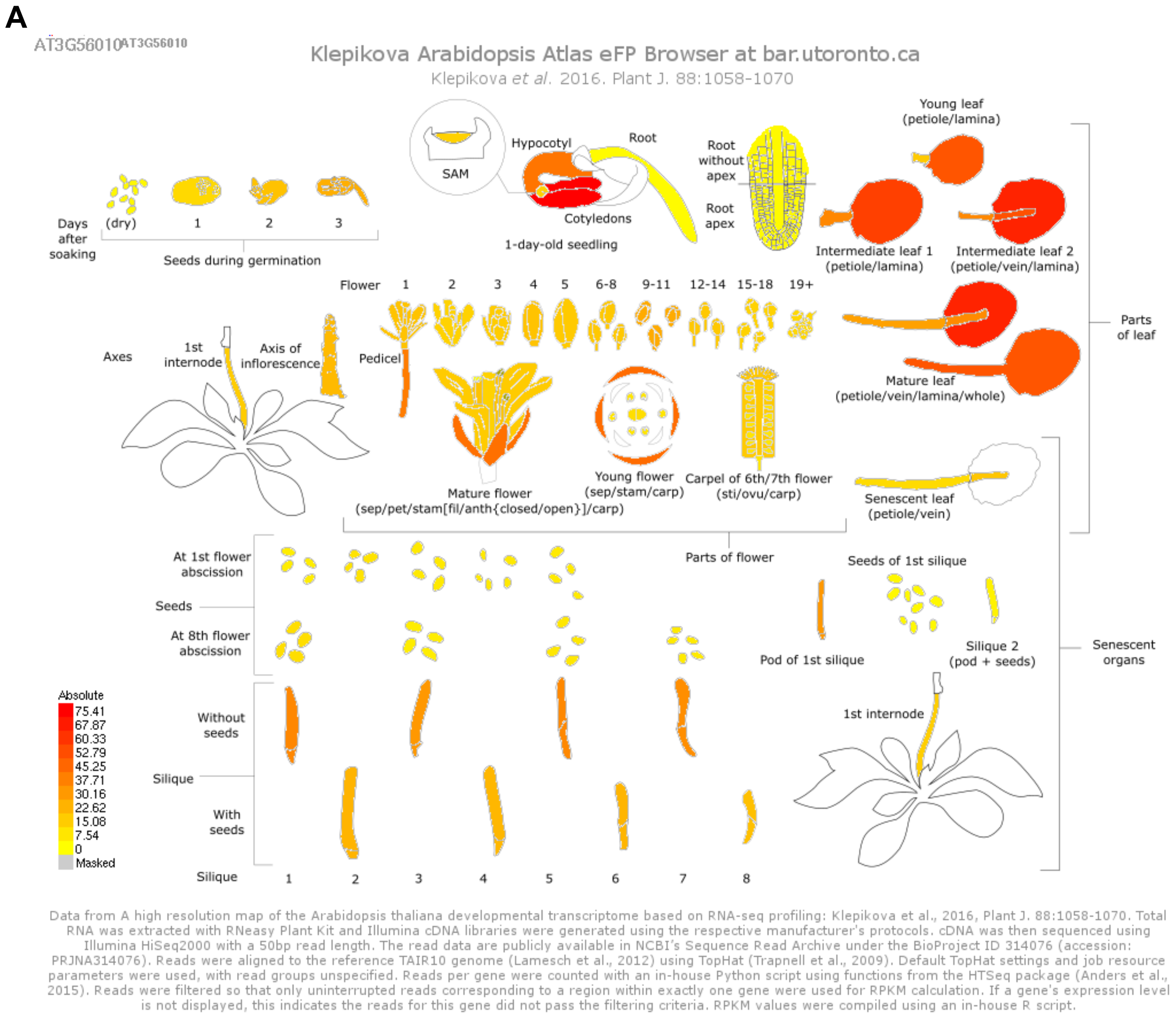


**B**

>CEPA1 tryptic sequence

MSVVLNAGFSSPLQNR SHHVIQLK PSPFASYISLNSSR R SLLCK R R

LVVSCLDTNDNSVTTTSVDSSSSSDSNK PVSESVESSNGTAK K APLTAR
PAp06471115

ER LR AAR VLSR YTEATPK PSK PK MGSQLLDVLK ESDK K SK R K

PGLPEAPTNMLDDSR R GMPK

SGLTFDLPGGSDILIIAFSFVFISTVMFATTFLVWK LGAIHFNE

**C**

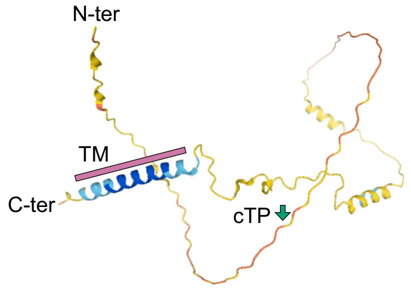


**Supplementary Figure S1.** Properties of the *CEPA1* gene in *Arabidopsis thaliana*. (Supports **Fig. 1** and **Table 1**)
**A)** Expression map of *CEPA1* (At3g56010) from the Arabidopsis Atlas (**Klepikova *et al.*, 2016**). Map was retrieved from the Arabidosis eFP Browser at https://bar.utoronto.ca/efp/cgi-bin/efpWeb.cgi?primaryGene=AT3G56010&dataSource=Klepikova_Atlas&modeInput=Absolute. The absolute expression in each tissue is indicated on a color scale from yellow to red (lowest to highest value). *CEPA1* is mostly expressed in developing green tissue (*i.e.*, cotyledons and leaves). **B)** Trypsin digestion map of CEPA1. The peptide PAp06471115 reported in the Arabidopsis PeptideAtlas (**van Wijk *et al.*, 2021**) is underlined in green. The cTP cleavage site is shown by a green arrow. The antigen and transmembrane (TM) sequences are shown in orange and pink, respectively. **C)** The CEPA1 precursor structure predicted by AlphaFold (AF-Q9LY44-F1; **Jumper *et al.*, 2021; Varadi *et al.*, 2022**). Structure was retrieved from the AlphaFold Protein Structure Database at https://alphafold.ebi.ac.uk/entry/Q9LY44. The residue colors indicate the model confidence: the blue region is predicted with high confidence while the yellow and orange regions are predicted with low confidence.

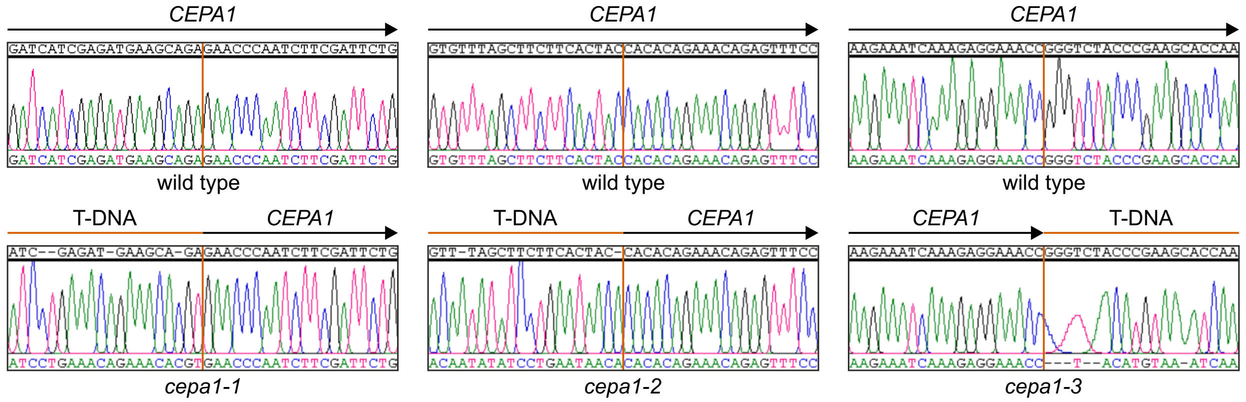


**Supplementary Figure S2.** Sequencing of the left borders of the T-DNA insertion sites in the *cepa1-1*, *cepa1-2* and *cepa1-3* mutant lines. (Supports **Fig. 1**)

The DNA region spanning the predicted T-DNA left border in the mutants was amplified by PCR from *cepa1-1* and *cepa1-2* (PCR 1 in **Supplementary Table S4**), *cepa1-3* (PCR 3 in **Supplementary Table S4**), and wild-type (WT; PCRs 2 and 4 in **Supplementary Table S4**) genomic DNA. Amplicon sequences were determined (LGC Genomics, Berlin, Germany) and aligned to the *CEPA1* gene sequence extracted from TAIR with SeqManPro 17 (version 17.2.0; DNASTAR Lasergene). The *CEPA1* gene reference and amplicon sequences are indicated above and below the chromatograms, respectively. The corresponding wild-type sequences are shown above the mutant sequences. The horizontal red bars mark the T-DNA sequence, and the vertical red bars indicate the T-DNA insertion sites in the genomes of the mutants. No deletion was observed at the T-DNA insertion site of each mutant. Adenine (A), cytosine (C), guanine (G) and thymine (T) are represented in green, blue, black and pink, respectively, in the amplicon sequences and the chromatograms.

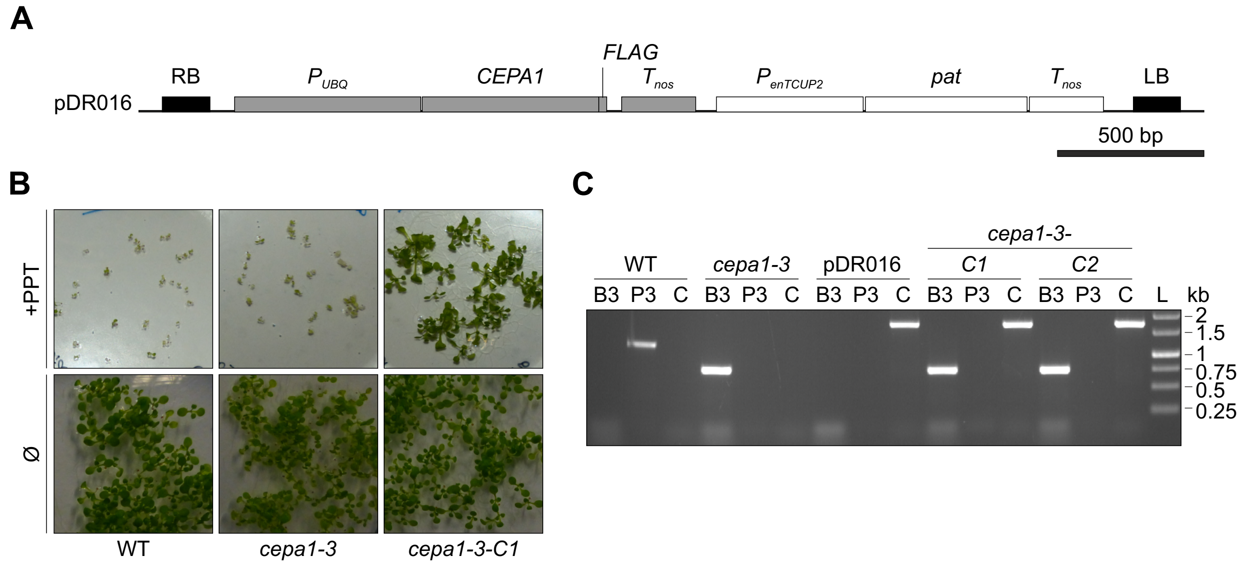


**Supplementary Figure S3.** Selection of the complemented lines. (Supports **Fig. 1**)
**A)** Physical map of the complementation cassette in vector pDR016. Black boxes indicate the T-DNA right and left borders (RB and LB, respectively) necessary for cassette insertion into the plant genome. The grey boxes indicate the $P_{UBQ}$:*CEPA1-FLAG* expression cassette composed of the *UBQ10* (At4g05320) promoter ($P_{UBQ}$), the *CEPA1* coding sequence, the FLAG tag and the *nos* terminator ($T_{nos}$). The open boxes indicate the selection cassette composed of the *enTCUP2* promoter ($P_{enTCUP2}$), the *phosphinothricin N-acetyltransferase* (*pat*) gene, and the *nos* terminator ($T_{nos}$) for selection of transformed plants on medium supplemented with phosphinothricin. Scale bar: 500 bp. **B)** Seedling survival test on selection medium. Wild-type, *cepa1-3* and *cepa1-3* seeds were sown on 0.5 MS medium supplemented with 1% (w/v) sucrose and 10 µg $mL^{-1}$ phosphinothricin (+ PPT) or without antibiotics (Ø), and germinated in long-day conditions. The picture was taken two weeks after seedling germination. The *cepa1-3-C1* complemented line survives on the selection medium, while WT and *cepa1-3* seedlings do not develop. **C)** Genotyping of complemented lines by PCR. The *cepa1-3* genomic background of the complemented lines was confirmed by PCR amplification of the region spanning the T-DNA insertion site. When the two primers are positioned upstream of the T-DNA insertion site and in the T-DNA left border (lanes B3 = PCR 3 in **Supplementary Table S4**), a 767 bp amplicon is expected only from the *cepa1-3* genomic background. When the two primers are positioned upstream and downstream of the T-DNA insertion site (lanes P3 = PCR 4 in **Supplementary Table S4**), a 1,159 bp amplicon is expected only from the wild-type (WT) genomic background. When the two primers are positioned upstream and downstream of the $P_{UBQ}$:*CEPA1-FLAG* cassette (lanes C = PCR 7 in **Supplementary Table S4**), a 1,750 bp amplicon is expected only with the vector pDR016. Thus, the complemented lines *cepa1-3-C1* and *cepa1-3-C2* carry the complete expression cassette in the *cepa1-3* background. PCR products were separated by 1% (w/v) agarose gel electrophoresis, and the GeneRuler 1 kb DNA Ladder (L; Thermo Scientific) was used for DNA band size estimation.

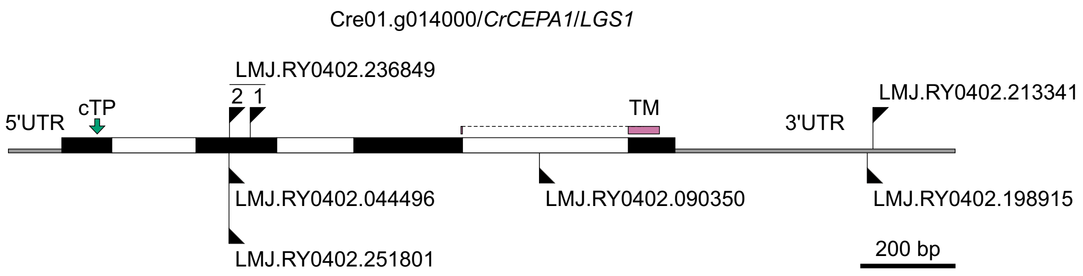


**Supplementary Figure S4.** Physical map of Cre01.g014000/*CrCEPA1*/*LGS1* and marker cassette insertion sites in CLiP mutants. (Supports **Fig. 2**)
Black and open boxes correspond to the four exons and the three introns, respectively. The green arrow shows the chloroplast transit peptide (cTP) cleavage site predicted by PredAlgo (**Tardif *et al.*, 2012**). The pink box shows the predicted transmembrane (TM) domain spanning exons 3 and 4. Black flags mark the insertion sites of the paromomycin resistance gene cassette in the genome of the different CLiP mutant lines. Scale bar: 200 bp.

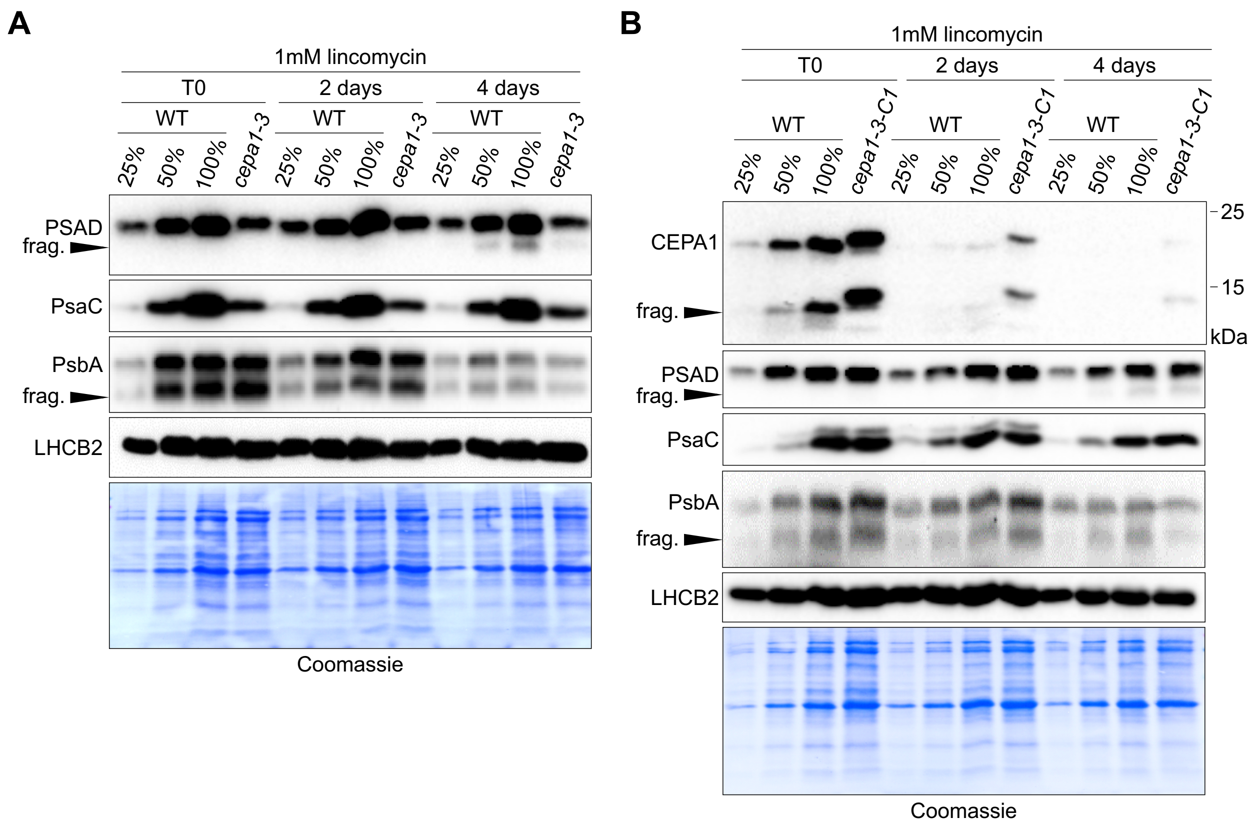


**Supplementary Figure S5.** CEPA1 and PSI subunit accumulation after lincomycin treatment. (Supports **Fig. 3**) 12-day-old *cepa1*-3, *cepa1-3*-C1 and wild-type seedlings grown on plates were infiltrated with 1 mM lincomycin, grown in low light (5 µmol photons $m^{-2}$ $s^{-1}$), and harvested 2 and 4 days after the treatment. As a control, seedlings infiltrated with water instead of lincomycin were harvested directly (T0). **A, B)** Proteins from isolated thylakoid membranes (equivalent to 2 µg chlorophyll) of *cepa1-3* and the wild type **(A)**, and *cepa1-3-C1* and the wild type **(B)** were separated by SDS-PAGE and subjected to immunoblotting with antibodies against PsaC, PSAD, PsbA and LHCB2 (and CEPA1 in panel B). A dilution series of the wild-type sample (25%, 50% and 100%) was loaded to be able to estimate protein accumulation in the mutants. The blotted membrane was stained with Coomassie as a loading control.

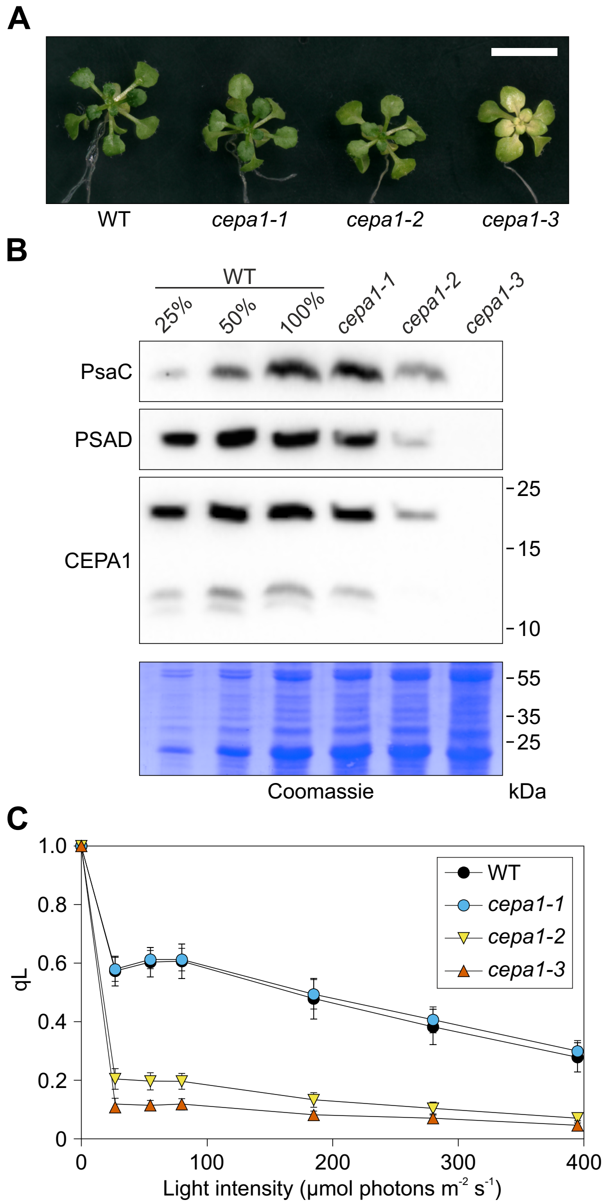


**Supplementary Figure S6.** Phenotyping of *cepa1* mutants grown in the cold. (Supports **Figs. 1** and **3**)
Seedlings were grown on plates for 5 days at 22°C, followed by 22 days in a diurnal cycle of 12°C during the day (16 hours) and 10°C during the night (10°C), at 100 µmol photons $m^{-2}$ $s^{-1}$. **A)** Comparison of plant development in T-DNA insertion lines and the wild type. Scale bar: 5 mm. **B)** Immunoblot against diagnostic PSI subunits and CEPA1. Proteins from isolated thylakoid membranes of each line (equivalent to 2 µg chlorophyll) were separated by SDS-PAGE, and subjected to immunoblotting with antibodies against PsaC, PSAD and CEPA1. A dilution series of the wild-type sample (25%, 50% and 100%) was loaded to estimate protein accumulation in the mutants. The blotted membrane was stained with Coomassie as a loading control. **C)** Light response curve of the qL parameter. Measurements were conducted on 16 seedlings per line. Error bars represent the standard deviation.

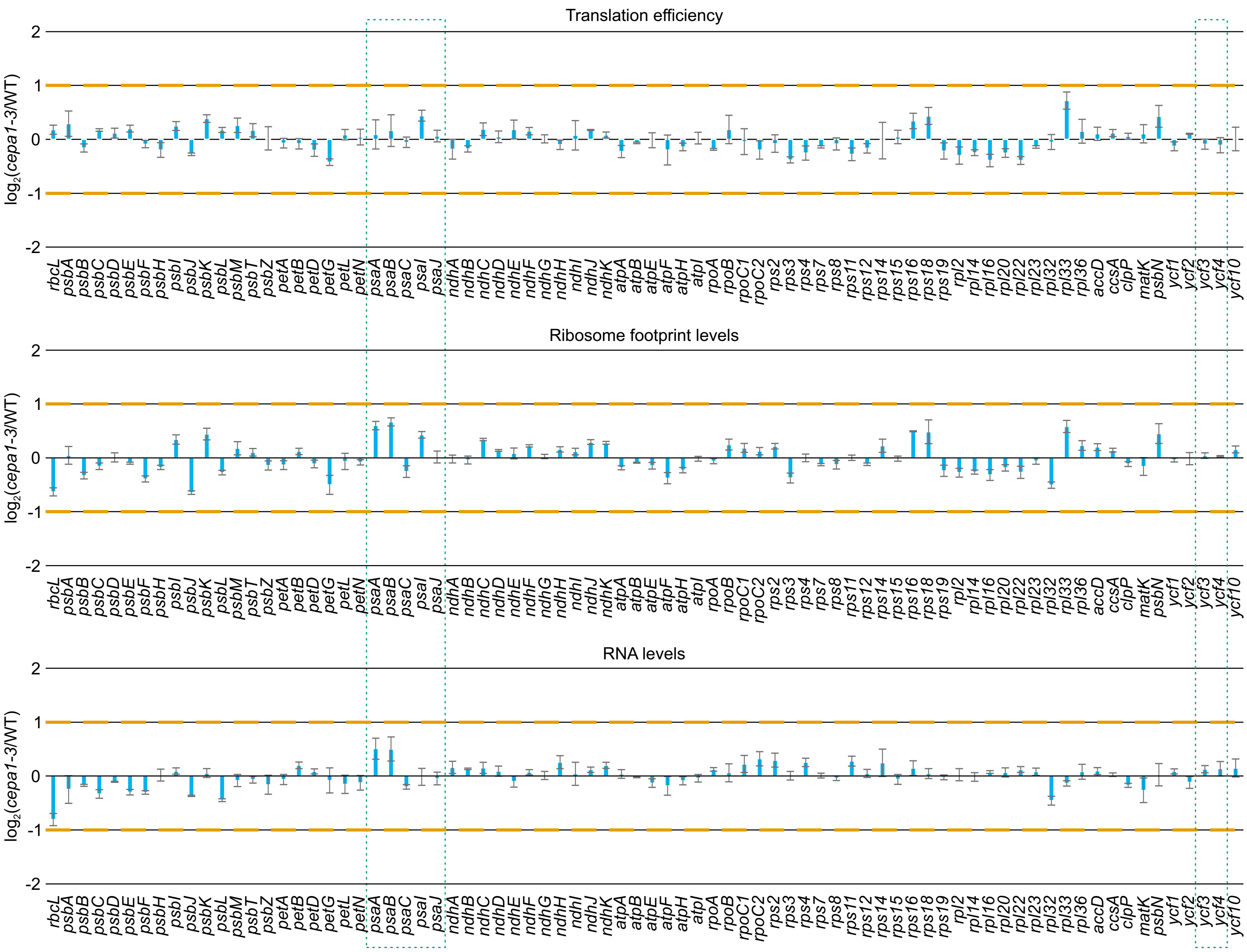


**Supplementary Figure S7.** Change of plastid RNA and ribosome footprint levels, and resulting translation efficiencies in *cepa1-3* in comparison to the wild type. (Supports **Fig. 6**)

Data for all plastid genes are shown. The green dotted frames highlight the PSI-related plastid genes: PSI subunit-encoding genes (*psaA*, *B*, *C*, *I*, and *J*) and PSI assembly factor-encoding genes (*ycf3* and *ycf4*). The dotted orange lines indicate the fold changes of 0.5 and 2 (*i.e.*, $\log_2(0.5) = -1$ and $\log_2(2) = 1$, respectively) in *cepa1-3* compared to the wild type (WT), the thresholds considered to be biologically relevant. The translation efficiency of the genes was obtained by normalizing the ribosome footprint levels to the RNA levels. The translation efficiency of all plastid genes is similar in *cepa1-3* and the wild type, including the *psaA-psaB-rps14* gene cluster. n = 3 independent biological replicates. Error bars represent the standard deviation. For more details, see **Supplementary Dataset S1**.

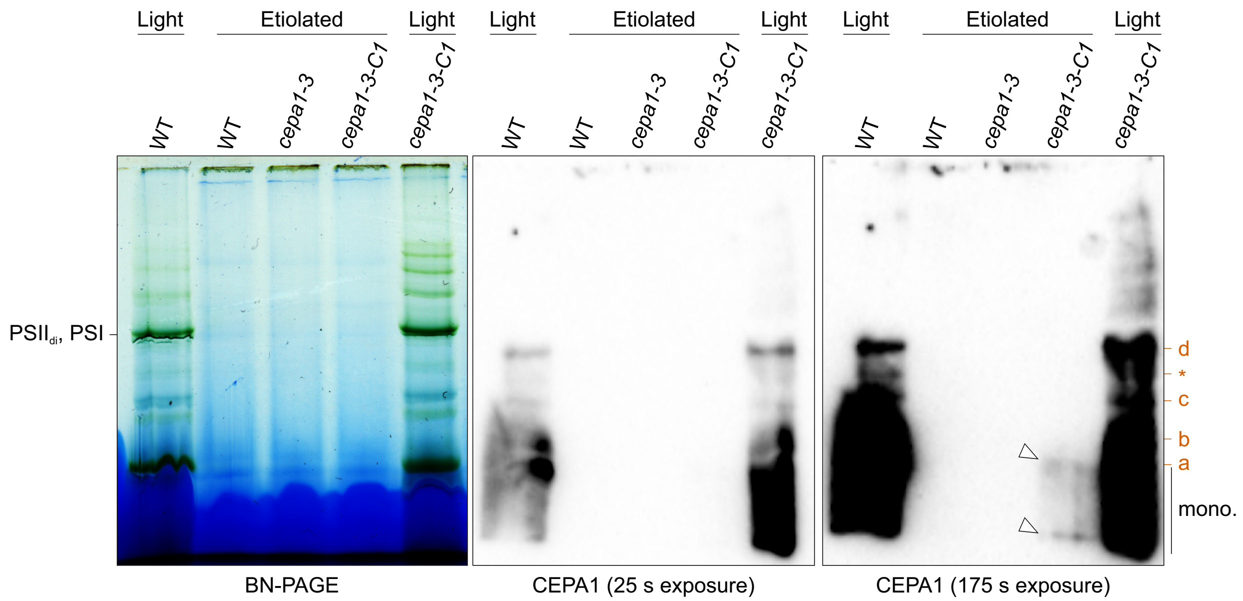


**Supplementary Figure S8.** CEPA1 does not associate with high-molecular weight complexes in the absence of PSI biogenesis. (Supports **Fig. 9**)
Native complexes from thylakoid membranes (equivalent to 5 µg chlorophyll) of light-grown plants and membranes (equivalent to 50 µg protein) of etiolated plants were solubilized in 1% (w/v) β-DM and separated by BN-PAGE. The native gel was scanned (left panel) and subjected to immunoblotting with antibodies against CEPA1 with a short (25 s; middle panel) and long (175 s; right panel) exposure time. Note that the strongest signals for light-grown wild type (WT) and *cepa1-3-C1* are saturated in the right panel. White arrows indicate the CEPA1 signals in the etiolated *cepa1-3-C1* sample. Red letters indicate the CEPA1-containing complexes in light-grown WT and *cepa1-3-C1* (*cf.* **Fig. 9**). PSII$_{di}$: PSII-LHCII dimer, PSI: mature PSI-LHCI, mono.: monomeric fraction.

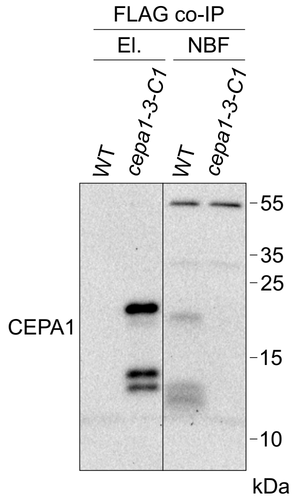


**Supplementary Figure S9.** The mature CEPA1-FLAG protein and CEPA1 fragments are pulled down by co-immunoprecipitation. (Supports **Table 2**)

Protein complexes from *cepa1-3-C1* and wild-type (WT) thylakoid membranes (non-cross-linked) were solubilized in 1% (w/v) β-DM, and subjected to co-immunoprecipitation (co-IP) using FLAG antibody-coated beads. Presence of CEPA1-FLAG in eluates (El.) and non-binding fractions (NBF) from the co-IP experiment was assessed by immunoblotting. Samples were separated by SDS-PAGE and subjected to immunoblotting using antibodies against CEPA1. Anti-IgG (Fc)-HRP secondary antibodies were used for detection of CEPA1 antibodies (avoiding cross-reaction with the denatured light chains of anti-FLAG antibodies at ~25 kDa). Both the El. and the NBF panels are from the same original blot - the black vertical line indicates unnecessary lanes that were digitally removed.

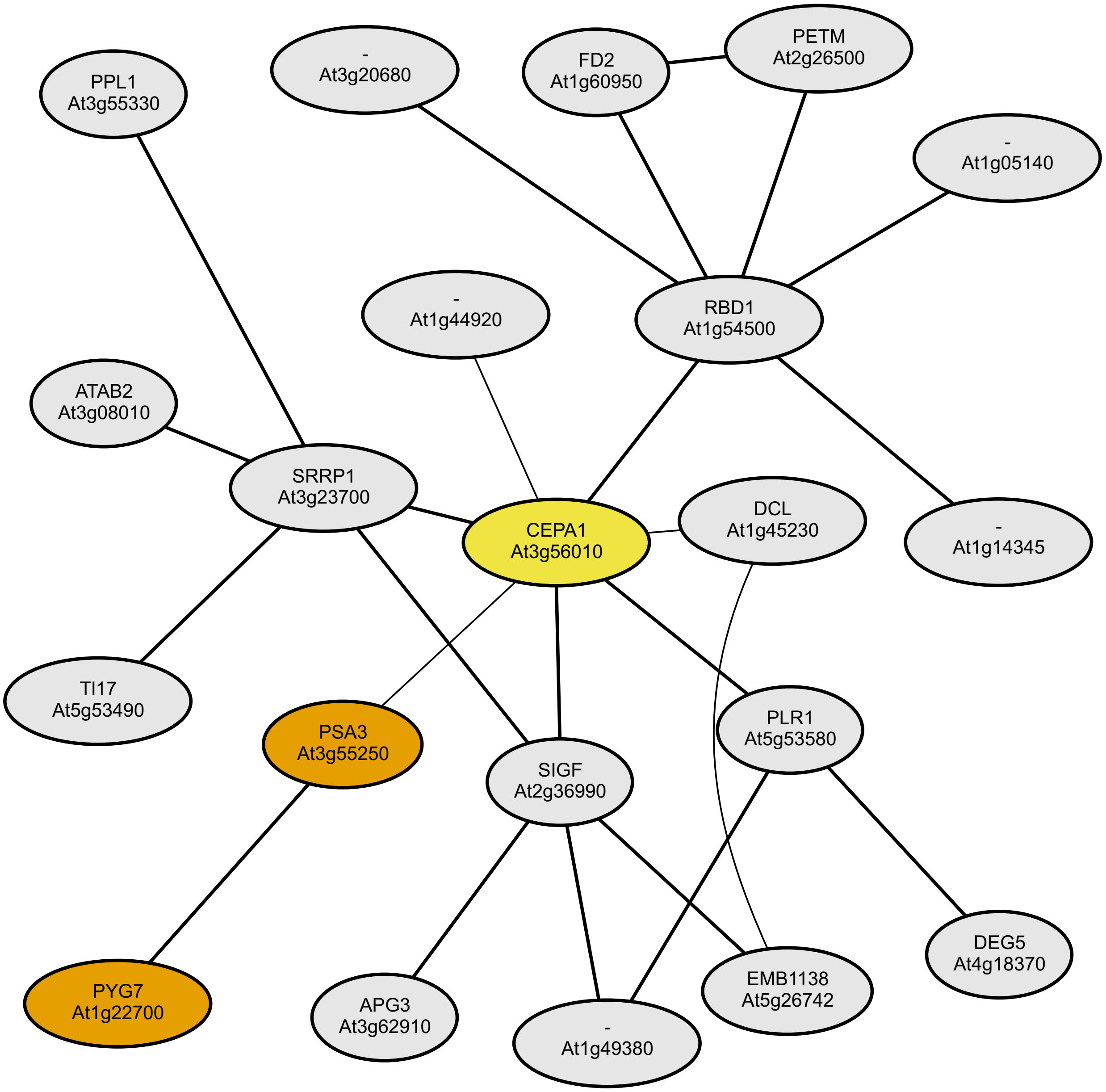


**Supplementary Figure S10.** *CEPA1* co-expression network. (Supports **Table 1**)
The diagram was extracted from the ATTED-II website (version 11.1; https://atted.jp/locus/?gene_id=AT3G56010; **Obayashi *et al.*, 2022**) and modified for clearer visualization. The AGI locus and alias (when applicable) of each gene of the network are indicated. *CEPA1* is highlighted in yellow, *PSA3* and *PYG7* are highlighted in orange.

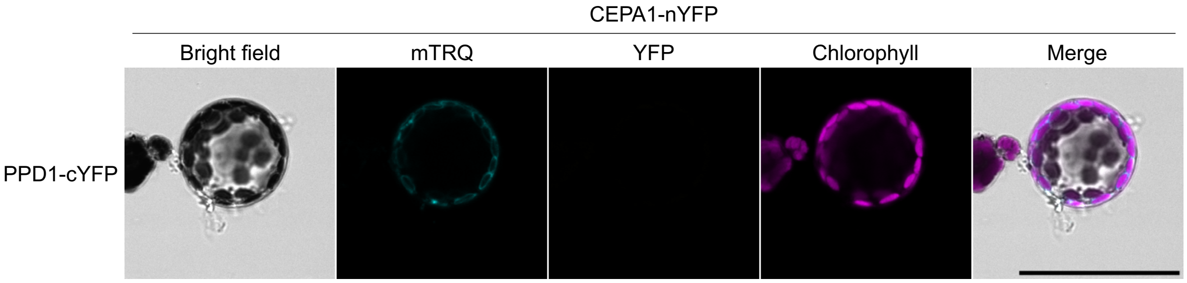


**Supplementary Figure S11.** Representative BiFC assay with nYFP fused to the C-terminus of CEPA1. (Supports **Fig. 13**)

The experiment was conducted as described in **Fig. 13**, but using the appropriate vector to test for CEPA1-nYFP interaction with PPD1-cYFP in BiFC. No YFP complementation was observed.

**Supplementary Table S1.** CEPA short-list of candidates obtained from EnsembleNet. (Supports **Fig. 1** and **Table 1**)
From the original EnsembleNet output list obtained with the nuclear genes *PYG7*, *Y3IP1*, *PPD1*, *PSA2* and *PSA3* as query, 22 genes of interest coding for proteins with predicted plastid-localization and uncharacterized function were selected for preliminary screening. Homozygous descendants of T-DNA insertion lines were identified by PCR genotyping (**Supplementary Table S4**), and five individuals per line were subjected to qL screening (at least one T-DNA insertion line per candidate gene). Some T-DNA insertion lines were wild type for the gene of interest, and, therefore, were not included in the qL screening. The qL screening identified At3g56010 as a PSI assembly factor candidate, with the *cepa1-3* mutant displaying a more severe qL LRC phenotype than the *cepa1-2* mutant (**Fig. 1B**). UTR: untranslated region. LB: left border primer of T-DNA. LP: left genomic primer.

| **Candidate gene** | **T-DNA insertion line** | **Insertion site** | **PCR genotyping** | |
|---|---|---|---|---|
| | | | **LB** | **LP** |
| At1g18060 | SALK_055016C | Intron 3 | PCR 5a | PCR 6a |
| At1g21500 | WiscDsLoxHs018_10F | 3’UTR | PCR 5b | PCR 6b |
| At1g64355 | SALK_026486C | 5’UTR | PCR 5c | PCR 6c |
| At1g64680 | SALK_021799 | 5’UTR | PCR 5d | PCR 6d |
| At1g65230 | SAIL_436_C01 | Exon 2 | PCR 5e | PCR 6e |
| At2g03420 | SALK_097642 | Exon 2 | PCR 5f | PCR 6f |
| At2g17972 | SAIL_287_D12 | 5’UTR | PCR 5g | PCR 6g |
| At2g29180 | SAIL_52_F11 | 3’UTR | PCR 5h | PCR 6h |
| | SALK_106162 | Exon 1 | PCR 5i | PCR 6i |
| At2g44920 | SAIL_651_G10 | Intron 1 | PCR 5j | PCR 6j |
| At3g09050 | GABI_065B12 | 5’UTR | PCR 5k | PCR 6k |
| | SALK_208285C | 5’UTR | PCR 5l | PCR 6l |
| At3g45050 | SALK_045946 | 5’UTR | PCR 5m | PCR 6m |
| At3g50685 | GABI_216E06 | Exon 1 | PCR 5n | PCR 6n |
| At3g56010 | SAIL_607_B08 (*cepa1-1*) | 5’UTR | PCR 1 | PCR 2 |
| (*CEPA1*) | SAIL_747_E01 (*cepa1-2*) | 5’UTR | PCR 1 | PCR 2 |
| | GABI_257C05 (*cepa1-3*) | Exon 2 | PCR 3 | PCR 4 |
| At3g61870 | WiscDsLox495F11 | Exon 1 | PCR 5o | PCR 6o |
| | SAIL_65_B07 | Exon 2 | PCR 5p | PCR 6p |
| At4g16410 | SALK_099132C | Exon 1 | PCR 5q | PCR 6q |
| At4g22830 | SAIL_333_D07 | 5’UTR | PCR 5r | PCR 6r |
| At4g24090 | SALK_012632 | Exon 2 | PCR 5s | PCR 6s |
| | GABI_124G02 | Exon 2 | PCR 5t | PCR 6t |
| At4g24930 | SALK_132657C | Exon 1 | PCR 5u | PCR 6u |
| At4g32590 | SALK_009697C | Exon 2 | PCR 5v | PCR 6v |
| At5g27290 | SALK_133015 | Intron 3 | PCR 5w | PCR 6w |
| At5g42070 | SALK_113372 | Exon 2 | PCR 5x | PCR 6x |
| | SALK_014079 | Intron 2 | PCR 5y | PCR 6x |
| At5g48790 | SALK_058635 | Intron 2 | PCR 5z | PCR 6y |

**Supplementary Table S2.** Sequences of the CEPA1 gene and protein in *Arabidopsis thaliana*. (Supports **Fig. 1**) In the *CEPA1* gene sequence, exons are indicated in black and introns in grey, 5'UTR and 3'UTR sequences are highlighted in grey, and the T-DNA insertion sites (and orientation) in *cepa1-1*, *cepa1-2*, and *cepa1-3* are highlighted with a blue, yellow, and red arrow, respectively. In the CEPA1 protein sequence, the chloroplast transit peptide, the predicted transmembrane domain and the antigen selected for antibody production are shown in green, pink and orange, respectively. For a clearer distinction between nucleotide and amino acid sequences, they are displayed in lowercase and uppercase characters, respectively. Gene and protein sequences were retrieved from TAIR (**Reiser *et al.*, 2022**) and UniProt (**The UniProt Consortium, 2021**) databases, respectively.

| CEPA1 | Accession number | Sequence |
|---|---|---|
| Gene | At3g56010 | tttaagagcgagtgggacgttgatcatcgagatgaagcaga→gaacccaatcttcgattctgtgtttagcttcttcactac<br>→cacacagaaacagagtttccgtctttcatcttcctccatatgcgtcgctcttaaaaacctaattcacaatgtccgtcgttctc<br>aacgccggcttcagttctccggtaagaatttgctctcttcctctttgataaccatataagattcttctactcatactcctgtataatt<br>tgctccagaaccactttaattgggattgaatcgtttactcttttgtgactgaaattcgaaagctttctcgtttcccttgatgaacttg<br>gttttgtagagattgattggtccttgattcgtctctctagagtttgaaaaaaccattgctttatgtacttcttcgatgcataaacca<br>atgctttcatataattccgctgatattaacaaaaatcttctctctttgaagcttcaaaacagatcacatcatgttatacagttaaa<br>gccttcgccttttgcgtcatacatatctctaaacagttcaagaaggtcactgttatgcaaaagaagattggttgtatcttgtttgg<br>atacaaatgacaattctgtcacaactacatccgtagattcttcttcttcttcagattctaataaaccagtaagtgaatcagttga<br>atcaagcaatggcactgccaaaaaagcaccattgacagcacgagagagactaagagcggctcgtgttcttagccgat<br>acactgaagcaacaccgaaaccgtcaaaacctaaaatggggagccaacttcttgatgtgctcaaggaaagtgataag<br>aaatcaaagaggaaacc←gggtctacccgaagcaccaactaacatgcttgatgatagcaggagagggatgccaaa<br>gagcggtcttacgtttgatttaccgggaggctcggatattctcatcattgctttctcctttgtgttcataagcacagtcatgtttgct<br>actacttttcttgtttggaaactcggtgcgatacacttcaacgaatagtaaagttgagatttggagaagtttaatggagtttgg<br>aaaccatcaagcagggaacgagagagagagagtgttttgttacaatgttagttaacaatgcatacttggggatgtgatgg<br>ggatcttcatcacttggcaaatccggcctgagtgtatatacatatactctgtagtacacataattttgctatgaactctcttcatg<br>cttgatttgtaattttaatctttacattgtaatttcttttcatatggaaatggatgcttaaagaccaacaacagccaatgtaaatat<br>gtatcagatgttagagagcaaaatatcacaagtttattttaagaagagaatgagagataggagggtttgggacaag |
| Protein | Q9LY44 | MSVVLNAGFSSPLQNRSHHVIQLKPSPFASYISLNSSRRSLLCKRRLVVSCLDTNDNS<br>VTTTSVDSSSSSDSNKPVSESVESSNGTAKKAPLTARERLRAARVLSRYTEATPKPSK<br>PKMGSQLLDVLKESDKKSKRKPGLPEAPTNMLDDSRRGMPKSGLTFDLPGGSDILIIA<br>FSFVFISTVMFATTFLVWKLGAIHFNE |

**Supplementary Table S3.** Details of CLiP strains harboring a mutation in the *CrCEPA1* gene. (Supports **Fig. 2**) All lines were generated in **Li *et al.* (2019)**. One to three paromomycin resistance cassettes were inserted into the genome of the strains of interest, and at least one cassette was inserted into the Cre01.g014000/*CrCEPA1*/*LGS1* gene. The orientation of the paromomycin resistance cassette is indicated relative to the orientation of the mutated gene. The confidence level corresponds to the estimated chance that the insertion site is correct, based on the PCR verification of insertion sites in randomly picked CLiP mutants, as detailed in **Li *et al.* (2019)**. The phenotype relates to the growth rate of the mutant in autotrophic compared to heterotrophic conditions (**Li *et al.*, 2019**). The CLiP database reports no phenotype associated with strains harboring mutations in Cre04.g217954 or Cre12.g560000 (**Li *et al.*, 2019**).

| | **Paromomycin resistance cassette(s)** | | | |
|---|---|---|---|---|
| **Strain ID** | **Insertion site(s)** | **Orientation** | **Confidence** | **Phenotype** |
| LMJ.RY0402.044496 | *CrCEPA1* exon 2 | Sense | 73% | No/little growth |
| | Cre04.g217954 intron | Sense | 95% | |
| | Cre04.g217954 CDS/intron | Sense | 95% | |
| LMJ.RY0402.236849 | *CrCEPA1* exon 2 | Antisense | 95% | No/little growth |
| | *CrCEPA1* exon 2 | Antisense | 95% | |
| LMJ.RY0402.251801 | *CrCEPA1* exon 2 | Antisense | 73% | No/little growth |
| LMJ.RY0402.090350 | *CrCEPA1* intron 3 | Sense | 73% | No/little growth |
| | Cre12.g560000 intron | Antisense | 73% | |
| LMJ.RY0402.198915 | *CrCEPA1* 3’UTR | Sense | 95% | Normal growth |
| LMJ.RY0402.213341 | *CrCEPA1* 3’UTR | Sense | 73% | Normal growth |

**Supplementary Table S4.** List of primers used in this study. (**Supports Fig. 1**)
Primers were synthesized by Sigma-Aldrich. In the primers for the In-Fusion and MoBiFC inserts, the CEPA1 and PsaC sequences are underlined in blue, the FLAG sequence in red, and the BbsI-recognition site in yellow. T-DNA primers were selected with the iSect Primers Tool (**O'Malley *et al.*, 2015**). LB: left border of T-DNA, LP: left genomic primer, RP: right genomic primer, for: forward primer, rev: reverse primer.

| **Primer** | **PCR** | **Description** | **Sequence 5'→3'** |
|---|---|---|---|
| oligo(dT)$_{18}$ | | cDNA synthesis | TTTTTTTTTTTTTTTTTT |
| SAIL_LB1 | PCRs 1 and 5e,g,h,j,p,r | LB primer | GCCTTTTCAGAAATGGATAAATAGC CTTGCTTCC |
| GABI_LB | PCRs 3 and 5k,n,t | LB primer | GACGTGAATGTAGACACGTCGA |
| SALK_LBb1.3 | PCRs 5a,c,d,f,i,l,m,q,s,u-z | LB primer | ATTTTGCCGATTTCGGAAC |
| Wisc_LB_P745 | PCRs 5b,o | LB primer | AACGTCCGCAATGTGTTATTAAGTT GTC |
| oJS105_LP | PCR 2 | *cepa1-1*, *-2* genotype | TAATCCACCGTTTCAATACGG |
| oJS106_RP | PCRs 1 and 2 | *cepa1-1*, *-2* genotype | ATATGTATGACGCAAAAGGCG |
| oDR395_LP | PCR 4 | *cepa1-3* genotype | TAGCAAAATTACGCCAGTGTG |
| oDR396_RP | PCRs 3 and 4 | *cepa1-3* genotype | TTGATTGGTCCTTGATTCGTC |
| oDR479_LP | PCR 6a | CEPA short list genotype | TTGGTTAAGTCATCGGTTTCG |
| oDR480_RP | PCRs 5a and 6a | CEPA short list genotype | CAAAAACTCGGAAAGTTGCAG |
| oDR481_LP | PCR 6b | CEPA short list genotype | TGGTTTCCAAATGTGCTCTTC |
| oDR482_RP | PCRs 5b and 6b | CEPA short list genotype | CTATGGAGTTGGAACGCTCTG |
| oDR489_LP | PCR 6c | CEPA short list genotype | AGAATCTCAGTCTGCGACGAG |
| oDR490_RP | PCRs 5c and 6c | CEPA short list genotype | TGCTCTGTGAATCAATGGTTG |
| oDR491_LP | PCR 6d | CEPA short list genotype | ATGCGTAGTTGATGGAGGTTG |
| oDR492_RP | PCRs 5d and 6d | CEPA short list genotype | CTCTTTGACACCTCCACGAAG |
| oDR049_LP | PCR 6e | CEPA short list genotype | CATTGTTGGTGACTTGGTGTG |
| oDR050_RP | PCRs 5e and 6e | CEPA short list genotype | AAACTTCTAACCGGATCCTGC |
| oDR059_LP | PCR 6f | CEPA short list genotype | ACTGTTCTAGGCCGAGCTACC |
| oDR060_RP | PCRs 5f and 6f | CEPA short list genotype | GCAATCTCCTACTCCCCATTC |
| oDR061_LP | PCR 6g | CEPA short list genotype | ACTTGCTACTCTCTCGCCTCC |
| oDR062_RP | PCRs 5g and 6g | CEPA short list genotype | TCCAACATAAAGCTCCAATGC |
| oDR505_LP | PCR 6h | CEPA short list genotype | TAGGCGATGATGAAGGATCAG |
| oDR506_RP | PCRs 5h and 6h | CEPA short list genotype | TTCTTGGGAAGAGGGAGAGAG |
| oDR031_LP | PCR 6i | CEPA short list genotype | TATCGACCATCAAGTTGCTTG |
| oDR032_RP | PCRs 5i and 6i | CEPA short list genotype | CATGGGATCGACTCATGTTTC |
| oDR509_LP | PCR 6j | CEPA short list genotype | GATTTTTGGTTTTGGTCTTTGG |
| oDR510_RP | PCRs 5j and 6j | CEPA short list genotype | GCAAGAGCAGGATCAACAAAG |
| oDR254_LP | PCR 6k | CEPA short list genotype | GCATATAAATCCACAATGGCG |
| oDR255_RP | PCRs 5k and 6k | CEPA short list genotype | GCTTCTTCAGCAAACTTGGTG |
| oDR324_LP | PCR 6l | CEPA short list genotype | TTCCCAATGCTGGAATACATC |
| oDR325_RP | PCRs 5l and 6l | CEPA short list genotype | GGAGAGACAGAGAAATTGGGG |
| oDR513_LP | PCR 6m | CEPA short list genotype | CCATCTTGTTCAGCTTCATGG |
| oDR514_RP | PCRs 5m and 6m | CEPA short list genotype | TGGTCAAGACAATGTTTGTCC |
| oDR517_LP | PCR 6n | CEPA short list genotype | GATGACGGTGAGGATGATGAC |
| oDR518_RP | PCRs 5n and 6n | CEPA short list genotype | ATAATCCCTGGCTAAATTGCG |

| | | | |
|---|---|---|---|
| oDR073_LP | PCR 6o | CEPA short list genotype | CCGTCATACCATCATGGGTAC |
| oDR074_RP | PCRs 5o and 6o | CEPA short list genotype | AGGTTTAAGGTACCTGCACCG |
| oDR257_LP | PCR 6p | CEPA short list genotype | TATCGTTCCCTTTCTGCATTG |
| oDR258_RP | PCRs 5p and 6p | CEPA short list genotype | CTTCTTCCTCGCACTCATCAC |
| oDR523_LP | PCR 6q | CEPA short list genotype | GGGATGAACAGAGGGAAAGAG |
| oDR524_RP | PCRs 5q and 6q | CEPA short list genotype | CTCCAGTGCTAACCGTGAGAG |
| oDR525_LP | PCR 6r | CEPA short list genotype | ACTCGGTCATGTCTCCATTTG |
| oDR526_RP | PCRs 5r and 6r | CEPA short list genotype | CAAAGCAGTAGCATCTGGTCC |
| oDR077_LP | PCR 6s | CEPA short list genotype | GAGCTTGTTCTCGAAATGGTG |
| oDR078_RP | PCRs 5s and 6s | CEPA short list genotype | TGTGAAAACAAGCTTTCCCAG |
| oDR075_LP | PCR 6t | CEPA short list genotype | TGCTTGATGGTCTGGAAAAAG |
| oDR076_RP | PCRs 5t and 6t | CEPA short list genotype | TTTGCCAGCTCATACTTCCAC |
| oDR529_LP | PCR 6u | CEPA short list genotype | CCTTTCTCTGCACAATTCTGC |
| oDR530_RP | PCRs 5u and 6u | CEPA short list genotype | TTCAAGAACCACAAACTTGGG |
| oDR531_LP | PCR 6v | CEPA short list genotype | TTGCTCAATCCATCCATCTTC |
| oDR532_RP | PCRs 5v and 6v | CEPA short list genotype | AAGTCACTTGCCTCCACAATG |
| oDR535_LP | PCR 6w | CEPA short list genotype | TTTTTGCAAAATCGTGAATGG |
| oDR536_RP | PCRs 5w and 6w | CEPA short list genotype | TAACGATAGCGGATGGAATTG |
| oDR039_LP | PCRs 5y and 6x | CEPA short list genotype | GAAGCATCGCAAGTTTATTGC |
| oDR040_RP | PCRs 5x and 6x | CEPA short list genotype | TCCAATTTCGTTGACATTAAGG |
| oDR541_LP | PCR 6y | CEPA short list genotype | TACGGGTATGCGACAAGAAAC |
| oDR542_RP | PCRs 5z and 6y | CEPA short list genotype | TGAAGAACGTGTGAGATTACGTC |
| oDR274_for | PCR 7 | *cepa1-3-C1*, *-C2* genotype | CCCAGTCACGACGTTGTAAAACG |
| oDR115_rev | PCR 7 | *cepa1-3-C1*, *-C2* genotype | CTTCAGGAAATAGAGATGCTTGCA |
| oDR316_for | PCR 8 | CEPA1-FLAG insert (In-Fusion) | GATTAACACTGAATTATGTCCGTCGTTCTCAACGC |
| oDR317_rev | PCR 8 | CEPA1-FLAG insert (In-Fusion) | CTAGTAAAAGGTACCCTACTTGTCATCGTCATCCTTATAATCTTCGTTGAAGTGTATCGCACCGA |
| oDR628_for | PCR 9 | CEPA1 insert (MoBiFC) | TTTGAAGACAAAGGTTTGGATACAAATGACAATTCTGTCACA |
| oDR629_rev | PCR 9 | CEPA1 insert (MoBiFC) | TTTGAAGACAACGAACCTTCGTTGAAGTGTATCGCACCGA |
| oDR626_for | PCR 10 | PsaC insert (MoBiFC) | TTTGAAGACAAAGGTTCACATTCAGTAAAAATTTATGATACTTGT |
| oDR627_rev | PCR 10 | PsaC insert (MoBiFC) | TTTGAAGACAACGAACCATAAGCTAGACCCATACTTCGAGT |

**Supplementary Table S5.** List of antibodies used in this study. (Supports **Figs. 1**, **3**, and **7-12**)
The dry commercial antibodies were dissolved in sterile mQ water. Working solutions of the primary antibodies were prepared by diluting the antibody stocks in TBS supplemented with 0.1% (v/v) Tween 20, 0.5% (w/v) BSA and 0.4% (v/v) Micr-O-protect (Roche). Working solutions of the secondary antibodies were prepared by diluting the antibody stock in the same solution without Micr-O-protect. All primary antibodies used in this study were raised in rabbit. Commercial secondary antibodies were raised in goat, either against both the heavy and light chains, or against the Fc portion only of rabbit IgG, and were conjugated with horseradish peroxidase (HRP) for chemiluminescence detection. Unless otherwise indicated, the standard secondary antibodies raised against both heavy and light chains of rabbit IgG were used.

| Target protein | AGI locus | Dilution | Company | Reference |
|---|---|---|---|---|
| ATPC | At4g04640 (1), At1g15700 (2) | 1:5,000 | Agrisera | AS08 312 |
| CEPA1 | At3g56010 | 1:1,000 | BioGenes | This study |
| CURT1A | At4g01150 | 1:500 | Agrisera | AS08 316 |
| LHCA1 | At3g54890 | 1:2,000 | Agrisera | AS01 005 |
| LHCA2 | At3g61470 | 1:2,000 | Agrisera | AS01 006 |
| LHCA3 | At1g61520 | 1:2,000 | Agrisera | AS01 007 |
| LHCA4 | At3g47470 | 1:2,000 | Agrisera | AS01 008 |
| LHCB2 | At2g05100 (.1), At2g05070 (.2), At3g27690 (.3) | 1:2,000 | Agrisera | AS01 003 |
| NdhH | AtCg01110 | 1:2,000 | Agrisera | AS16 4065 |
| PetA | AtCg00540 | 1:2,000 | Agrisera | AS06 119 |
| PsaA | AtCg00350 | 1:1,000 | Agrisera | AS06 172 |
| PsaB | AtCg00340 | 1:1,000 | Agrisera | AS10 695 |
| PsaC | AtCg01060 | 1:1,000 | Agrisera | AS10 939 |
| PSAD | At4g02770(1), At1g03130 (2) | 1:5,000 | Agrisera | AS09 461 |
| PSAF | At1g31330 | 1:5,000 | Agrisera | AS06 104 |
| PSAG | At1g55670 | 1:5,000 | Agrisera | AS04 048 |
| PSAH | At3g16240 (1), At1g52230 (2) | 1:1,000 | Agrisera | AS06 105 |
| PSAK | At1g30380 | 1:2,000 | Agrisera | AS04 049 |
| PSAL | At4g12800 | 1:1,000 | Agrisera | AS06 108 |
| PSAN | At5g64040 | 1:5,000 | Agrisera | AS06 109 |
| PSAO | At1g08380 | 1:5,000 | Agrisera | AS04 050 |
| PsbA | AtCg00020 | 1:2,500 | Agrisera | AS10 704 |
| PsbB | AtCg00680 | 1:2,000 | Agrisera | AS04 038 |
| PsbD | AtCg00270 | 1:5,000 | Agrisera | AS06 146 |
| PSBO | At5g66570 (1), At3g50820 (2) | 1:2,500 | Agrisera | AS05092 |
| RBCS | At1g67090 (1A), At5g38430 (1B) | 1:5,000 | Agrisera | AS07 259 |
| Rabbit IgG | - | 1:75,000 | Agrisera | AS09 602 |
| Rabbit IgG (Fc) | - | 1:75,000 | Invitrogen | 31463 |

**Supplementary Table S6.** List of vectors used in MoBiFC. (Supports **Fig. 13**)
Each level 0 vector contains a different module of the expression cassettes. $P_{35S}$ + $5'UTR_{35S}$ and $3'UTR_{35S}$ + $T_{35S}$ represent the cauliflower mosaic virus 35S promoter and 5' untranslated region (UTR), and the 35S 3'UTR and terminator, respectively. The chloroplast transit peptide (cTP) of the RubisCO small subunit (RBCS) from *Arabidopsis thaliana* is used to target all fusion proteins to the chloroplast. The genes of interest (GOI) cloned in CDS2ns modules correspond to the coding DNA sequence (CDS) after removal of the start codon, the cTP (for nucleus-encoded proteins), the lumenal signal peptide (for PPD1) and the stop codon. PPD1 and PSA3 were codon-optimized to remove the internal BbsI and BsaI restriction sites. nYFP and cYFP correspond to the $YFP_{V2\text{-}D174}$ and $YFP_{G175\text{-}K239}$ moieties, respectively. Each level 1 vector contains a different expression cassette (*i.e.*, transcription unit; **Supplementary Table S7**). All genes are expressed under the 35S promoter and 5'UTR (indicated by $P_{35S}$). The *p19* and *OEP7-mTRQ* cassettes were constructed with the *nos* terminator, while the tagged GOI cassettes were constructed with the 35S 3'UTR and terminator. The 3xFLAG-nYFP and cYFP-3xHA tags are denoted by nYFP and cYFP, respectively. Each level 2 vector contains the four expression cassettes (multigene units) necessary to conduct the BiFC assays. The p19 and OEP7-mTRQ cassettes are common to all multigene units. The two other cassettes correspond to the nYFP-tagged CEPA1 (N- or C-terminal fusion) and the cYFP-tagged candidate (N- or C-terminal fusion), whose interaction is being tested. The double selection of positive clones is based on colony resistance to antibiotics and color in blue-white (BW) assay (LB + 2% (w/v) agar supplemented with appropriate antibiotics, 20 µg $mL^{-1}$ X-Gal and 100 µM IPTG) or red-white (RW) assay (LB + 2% (w/v) agar supplemented with appropriate antibiotics). Correct sequences were further confirmed by Sanger sequencing. $Spec^R$, $Carb^R$, $Kan^R$ indicate that the vector confers resistance to spectinomycin, carbenicillin and kanamycin, respectively, in bacteria with the following antibiotic concentrations in the medium: 100 µg $mL^{-1}$ spectinomycin, 100 µg $mL^{-1}$ carbenicillin or 50 µg $mL^{-1}$ kanamycin.

| | Vector ID | MoClo syntax | Description | Selection | Addgene ID | Reference |
|---|---|---|---|---|---|---|
| MoBiFC level 0 : Modules | pICSL13001 | Pro-5U | $P_{35S}$ + $5'UTR_{35S}$ | $Spec^R$, white (BW) | 50265 | Engler *et al.*, 2014 |
| | pICH51266 | Pro-5U-NT1 | $P_{35S}$ + $5'UTR_{35S}$ | $Spec^R$, white (BW) | 50267 | Engler *et al.*, 2014 |
| | pLBC0010 | NT1 | RBCS cTP | $Spec^R$, white (BW) | 172774 | Velay *et al.*, 2022 |
| | pLBC0011 | NT2 | RBCS cTP | $Spec^R$, white (BW) | 172775 | Velay *et al.*, 2022 |
| | pLBC0050 | NT2 | 3xFLAG-nYFP | $Spec^R$, white (BW) | 172781 | Velay *et al.*, 2022 |
| | pLBC0060 | NT2 | cYFP-3xHA | $Spec^R$, white (BW) | 172783 | Velay *et al.*, 2022 |
| | pLBC0100 | CT | STOP | $Spec^R$, white (BW) | 172789 | Velay *et al.*, 2022 |
| | pLBC0051 | CT | 3xFLAG-nYFP + STOP | $Spec^R$, white (BW) | 172782 | Velay *et al.*, 2022 |
| | pLBC0061 | CT | cYFP-3xHA + STOP | $Spec^R$, white (BW) | 172784 | Velay *et al.*, 2022 |
| | pICH41414 | 3U-Ter | $3'UTR_{35S}$ + $T_{35S}$ | $Spec^R$, white (BW) | 50337 | Engler *et al.*, 2014 |
| | pAGM1299 | CDS2ns | Backbone for cloning of level 0 GOIs | $Spec^R$, blue (BW) | 47988 | Weber *et al.*, 2011 |
| | pDR140 | CDS2ns | CEPA1 | $Spec^R$, white (BW) | - | This study |
| | pDR146 | CDS2ns | PPD1 | $Spec^R$, white (BW) | - | This study |
| | pDR147 | CDS2ns | PSA3 | $Spec^R$, white (BW) | - | This study |
| | pDR151 | CDS2ns | PsaC | $Spec^R$, white (BW) | - | This study |

| | | | | | | |
|---|---|---|---|---|---|---|
| MoBiFC level 1: Transcript units | pLBT0160 | Position 1 | $P_{35S}$:*p19* | Carb$^{R}$, white (BW) | 172812 | Velay *et al.*, 2022 |
| | pLBT0190 | Position 4 | $P_{35S}$:*OEP7-mTRQ* | Carb$^{R}$, white (BW) | 172815 | Velay *et al.*, 2022 |
| | pICH41780 | - | End-linker of four level 1 vectors into level 2 | Spec$^{R}$, white (BW) | 48019 | Weber *et al.*, 2011 |
| | pICH47742 | Position 2 | Backbone for cloning of level 1 nYFP fusion | Carb$^{R}$, blue (BW) | 48001 | Weber *et al.*, 2011 |
| | pICH47751 | Position 3 | Backbone for cloning of level 1 cYFP fusion | Carb$^{R}$, blue (BW) | 48002 | Weber *et al.*, 2011 |
| | pDR157 | Position 2 | $P_{35S}$:*nYFP-CEPA1* | Carb$^{R}$, white (BW) | - | This study |
| | pDR158 | Position 2 | $P_{35S}$:*CEPA1-nYFP* | Carb$^{R}$, white (BW) | - | This study |
| | pDR159 | Position 3 | $P_{35S}$:*cYFP-CEPA1* | Carb$^{R}$, white (BW) | - | This study |
| | pDR160 | Position 3 | $P_{35S}$:*CEPA1-cYFP* | Carb$^{R}$, white (BW) | - | This study |
| | pDR174 | Position 3 | $P_{35S}$:*PPD1-cYFP* | Carb$^{R}$, white (BW) | - | This study |
| | pDR176 | Position 3 | $P_{35S}$:*PSA3-cYFP* | Carb$^{R}$, white (BW) | - | This study |
| | pDR184 | Position 3 | $P_{35S}$:*PsaC-cYFP* | Carb$^{R}$, white (BW) | - | This study |
| MoBiFC level 2 : Multigene units | pAGM4673 | - | Backbone for cloning of level 2 final vectors | Kan$^{R}$, red (RW) | 48014 | Weber *et al.*, 2011 |
| | pDR191 | - | nYFP-CEPA1 vs. cYFP-CEPA1 | Kan$^{R}$, white (RW) | - | This study |
| | pDR223 | - | CEPA1-nYFP vs. CEPA1-cYFP | Kan$^{R}$, white (RW) | - | This study |
| | pDR194 | - | nYFP-CEPA1 vs. PPD1-cYFP | Kan$^{R}$, white (RW) | - | This study |
| | pDR222 | - | CEPA1-nYFP vs. PPD1-cYFP | Kan$^{R}$, white (RW) | - | This study |
| | pDR196 | - | nYFP-CEPA1 vs. PSA3-cYFP | Kan$^{R}$, white (RW) | - | This study |
| | pDR201 | - | nYFP-CEPA1 vs. PsaC-cYFP | Kan$^{R}$, white (RW) | - | This study |

**Supplementary Table S7.** Cloning of MoBiFC vectors level 1 transcription units. (Supports **Fig. 13**)
List of level 0 modules and backbones used for each level 1 cloned in this study (**Supplementary Table S6**).

| | Vector | Description | pDR157 | pDR158 | pDR159 | pDR160 | pDR174 | pDR176 | pDR184 |
|---|---|---|---|---|---|---|---|---|---|
| Backbone | pICH47742 | (for nYFP fusions) | Yes | Yes | - | - | - | - | - |
| | pICH47751 | (for cYFP fusions) | - | - | Yes | Yes | Yes | Yes | Yes |
| vector level 0 modules | pICSL13001 | $P_{35S}$ + $5'UTR_{35S}$ | Yes | - | Yes | - | - | - | - |
| | pICH51266 | $P_{35S}$ + $5'UTR_{35S}$ | - | Yes | - | Yes | Yes | Yes | Yes |
| | pLBC0010 | RBCS cTP | Yes | - | Yes | - | - | - | - |
| | pLBC0011 | RBCS cTP | - | Yes | - | Yes | Yes | Yes | Yes |
| | pLBC0050 | 3xFLAG-nYFP | Yes | - | - | - | - | - | - |
| | pLBC0060 | cYFP-3xHA | - | - | Yes | - | - | - | - |
| | pLBC0100 | STOP | Yes | - | Yes | - | - | - | - |
| | pLBC0051 | 3xFLAG-nYFP + STOP | - | Yes | - | - | - | - | - |
| | pLBC0061 | cYFP-3xHA + STOP | - | - | - | Yes | Yes | Yes | Yes |
| | pICH41414 | $3'UTR_{35S}$ + $T_{35S}$ | Yes | Yes | Yes | Yes | Yes | Yes | Yes |
| | pDR140 | CEPA1 | Yes | Yes | Yes | Yes | - | - | - |
| | pDR146 | PPD1 | - | - | - | - | Yes | - | - |
| | pDR147 | PSA3 | - | - | - | - | - | Yes | - |
| | pDR151 | PsaC | - | - | - | - | - | - | Yes |

**Supplementary Table S8** List of accession numbers for the genes relevant to this study.
Gene loci in *Arabidopsis thaliana* (with homologs) are indicated, together with the *Chlamydomonas reinhardtii* gene homolog, if relevant. Sequences can be retrieved from TAIR and Phytozome databases.

| Alias | Full name/Description | Gene |
|---|---|---|
| *ATPC1, 2* | *(Plastidial) ATP SYNTHASE SUBUNIT γ1, 2* | At4g04640 (1), At1g15700 (2) |
| *CEPA1* | *CO-EXPRESSED WITH PSI ASSEMBLY 1* | At3g56010/Cre01.g014000 |
| *CURT1A* | *CURVATURE THYLAKOID 1A* | At4g01150 |
| *EMB3136* | *EMBRYO DEFECTIVE 3136* | At5g13510 |
| *LHCA1* | *LIGHT-HARVESTING COMPLEX I SUBUNIT 1* | At3g54890 |
| *LHCA2* | *LIGHT-HARVESTING COMPLEX I SUBUNIT 2* | At3g61470 |
| *LHCA3* | *LIGHT-HARVESTING COMPLEX I SUBUNIT 3* | At1g61520 |
| *LHCA4* | *LIGHT-HARVESTING COMPLEX I SUBUNIT 4* | At3g47470 |
| *LHCB2. 1, 2, 3* | *LIGHT-HARVESTING COMPLEX II SUBUNIT 2.1, 2, 3* | At2g05100 (1), At2g05070 (2), At3g27690 (3) |
| *nad9* | *(Mitochondrial) NADH dehydrogenase subunit 9* | AtMg00070 |
| *ndhH* | *(Plastidial) NADH-dehydrogenase-like subunit H* | AtCg01110 |
| *nptII* | *Neomycin phosphotransferase II* | - |
| *OEP7* | *OUTER ENVELOPE MEMBRANE PROTEIN 7* | At3g52420 |
| *pat* | *Phosphinothricin N-acetyltransferase* | - |
| *petA* | *Photosynthetic electron transfer A (Cytochrome f)* | AtCg00540 |
| *PPD1* | *PSBP-DOMAIN PROTEIN 1* | At4g15510/Cre08.g362900 |
| *PSA2* | *PHOTOSYSTEM I ASSEMBLY 2* | At2g34860 |
| *PSA3* | *PHOTOSYSTEM I ASSEMBLY 3* | At3g55250 |
| *psaA* | *Photosystem I subunit A* | AtCg00350 |
| *psaB* | *Photosystem I subunit B* | AtCg00340 |
| *psaC* | *Photosystem I subunit C* | AtCg01060 |
| *PSAD1, 2* | *PHOTOSYSTEM I SUBUNIT D1, 2* | At4g02770 (1), At1g03130 (2) |
| *PSAE1, 2* | *PHOTOSYSTEM I SUBUNIT E1, 2* | At4g28750 (1), At2g20260 (2) |
| *PSAF* | *PHOTOSYSTEM I SUBUNIT F* | At1g31330 |
| *PSAG* | *PHOTOSYSTEM I SUBUNIT G* | At1g55670 |
| *PSAH1, 2* | *PHOTOSYSTEM I SUBUNIT H1, 2* | At3g16140 (1), At1g52230 (2) |
| *psaI* | *Photosystem I subunit I* | AtCg00510 |
| *psaJ* | *Photosystem I subunit J* | AtCg00630 |
| *PSAK* | *PHOTOSYSTEM I SUBUNIT K* | At1g30380 |
| *PSAL* | *PHOTOSYSTEM I SUBUNIT L* | At4g12800 |
| *PSAN* | *PHOTOSYSTEM I SUBUNIT N* | At5g64040 |
| *PSAO* | *PHOTOSYSTEM I SUBUNIT O* | At1g08380 |
| *psbA* | *Photosystem II subunit A (D1)* | AtCg00020 |
| *psbB* | *Photosystem II subunit B (CP47)* | AtCg00680 |
| *psbD* | *Photosystem II subunit D (D2)* | AtCg00270 |
| *PSBO1, 2* | *PHOTOSYSTEM II SUBUNIT O1, 2* | At5g66570 (1), At3g50820 (2) |
| *PYG7* | *PALE YELLOW GREEN 1* | At1g22700 |
| *RBCS1A, B* | *RuBisCO SMALL SUBUNIT 1A, B* | At1g67090 (A), At5g38430 (B) |
| *RbcL* | *RuBisCO large subunit* | AtCg00490 |
| *RPL12-C* | *RIBOSOMAL PROTEIN L12-C* | At3g27850 |
| *rps14* | *Chloroplast ribosomal protein S14* | AtCg00330 |
| *TOM40* | *TRANSLOCASE OF THE OUTER MITOCHONDRIAL MEMBRANE 40* | At3g20000 |
| *UBQ10* | *UBIQUITIN 10 (promoter)* | At4g05320 |
| *Y3IP1* | *Ycf3-INTERACTING PROTEIN 1* | At5g44650 |
| *ycf3* | *Hypothetical chloroplast reading frame number 3* | AtCg00360 |
| *ycf4* | *Hypothetical chloroplast reading frame number 4* | AtCg00520 |
| - | (CEPA short-list candidate 2) | At1g18060 |
| - | (CEPA short-list candidate 3) | At1g21500 |
| - | (CEPA short list candidate 4) | At1g64355 |
| - | (CEPA short list candidate 5) | At1g64680 |
| - | (CEPA short list candidate 6) | At1g65230 |

| | | |
|---|---|---|
| - | (CEPA short list candidate 7) | At2g03420 |
| - | (CEPA short list candidate 8) | At2g17972 |
| - | (CEPA short list candidate 9) | At2g29180 |
| - | (CEPA short list candidate 10) | At2g44920 |
| - | (CEPA short list candidate 11) | At3g09050 |
| - | (CEPA short list candidate 12) | At3g45050 |
| - | (CEPA short list candidate 13) | At3g50685 |
| - | (CEPA short list candidate 14) | At3g61870 |
| - | (CEPA short list candidate 15) | At4g16410 |
| - | (CEPA short list candidate 16) | At4g22830 |
| - | (CEPA short list candidate 17) | At4g24090 |
| - | (CEPA short list candidate 18) | At4g24930 |
| - | (CEPA short list candidate 19) | At4g32590 |
| - | (CEPA short list candidate 20) | At5g27290 |
| - | (CEPA short list candidate 21) | At5g42070 |
| - | (CEPA short list candidate 22) | At5g48790 |
