# Supplementary material for: CO-EXPRESSED WITH PSI ASSEMBLY1 (CEPA1) is a photosystem I assembly factor in Arabidopsis
Source: Plant Cell. 2024 Feb 21;36(10):4179–211. doi: 10.1093/plcell/koae042 (PMC11449006; doi:10.1093/plcell/koae042)
Supplement: koae042_Supplementary_Data [file tpc.00879.2023-s05.pdf › tpc.00879.2023-s05.pdf]

THE PLANT CELL

CO-EXPRESSED WITH PSI ASSEMBLY 1 (CEPA1), a novel factor involved in photosystem I assembly in Arabidopsis

David Rolo, Omar Sandoval-Ibáñez, Wolfram Thiele, Mark Aurel Schöttler, Ines Gerlach, Reimo Zoschke, Joram Schwartzmann, Etienne Meyer, and Ralph Bock

Review Timeline:

Submission Date:	18-Sep-2023
Editorial Decision:	19-Oct-2023
Revision Received:	12-Jan-2024
Accepted:	24-Jan-2024

Prof. Dr. Ralph Bock
Max-Planck-Institut fur molekulare Pflanzenphysiologie
Dept. III
D-14476 Potsdam-Golm
Germany

Dear Dr. Bock/Dear Ralph,

We have received reviews of your manuscript entitled "CO-EXPRESSED WITH PSI ASSEMBLY 1 (CEPA1), a novel factor involved in photosystem I assembly in Arabidopsis." Thank you for submitting your best work to The Plant Cell. The editorial board agrees that the work you describe is substantive, falls within the scope of the journal, and may become acceptable for publication, pending revision and potential re-review.

In preparing your revision, please address all the specific points and questions raised by the reviewers. Streamline the presentation of the data as suggested, moving some to the supplemental section, and condensing detailed descriptions where appropriate. Finally, providing direct evidence to confirm the interaction between CEPA1 and PSI subunits would strengthen the work. However, if this is not possible, please discuss the limitation of the results of Figure 12.

Please contact us if there are ambiguous comments or if you wish to discuss the revision.

Given the nature of the comments, we are offering you 60 days from when we have issued this decision to complete the revision. If a revision is not returned within this time frame, and if you have not been granted an extension, we will withdraw the manuscript, which will leave you free to submit the work elsewhere. If you need an extension, we encourage you to contact us at any point before the 60 days have passed.

When you are ready to submit the revised version, please upload a highlighted copy that indicates all changes made in response to the editor and reviewer recommendations. Include an itemized list of all changes made in response to each of the reviewer's suggestions in the "Response to Reviewers" section; please note that reviewers do not have access to your cover letter, nor was this decision letter shared with them.

Thank you for the privilege of reviewing your work. We look forward to receiving your revised manuscript.

On behalf of the editorial board,

Roberta Croce, Board of Reviewing Editors
Nancy Eckardt, Senior Editor
Blake Meyers, Editor-in-Chief

The Plant Cell

Please note the following:

-The Plant Cell now requires authors to complete and submit an author revisions checklist upon submission of a revised manuscript. The aim of the checklist is to aid authors in preparing a high-quality manuscript, facilitate the review and assessment of revised manuscripts, and help to ensure that journal standards are maintained across the board. If your manuscript is accepted, the completed checklist will be published as supplemental material attached to the article online. Please download a copy of the checklist (pdf fillable form) at this link, for submission with your revised manuscript: https://tpc.msubmit.net/html/Author_Revisions_Checklist.pdf.

-Supplemental materials should be restricted to large datasets and tables, presentation of replicates, and validation of reagents, methods, or genotypes. Any data that are used to support the major claims must be in the main manuscript. Supplemental figure legends must indicate what figure in the main manuscript is supported by the supplemental data presented. Please justify how each of the supplemental figures meet the criteria.

-Sampling methods and nature of "biological replicates" should be described precisely (i.e. different plants, parts of plants, pooled tissue, independent pools of tissue, sampled at different times, etc), along with a clear description of and rationale for any statistical analyses conducted. The reader should know exactly what was sampled; what forms the basis of the calculation of any means and statistical parameters reported. This is also necessary to ensure that proper statistical analysis was conducted.

-Want to add this revision deadline to your calendar? Click below!

-- Reviewer comments:

Reviewer #1 (Comments for the Author):

The manuscript by Rolo et al. describes the identification and characterization of a new factor for the assembly of photosystem I in Arabidopsis thaliana. Based on a bioinformatical analysis of genes that are co-expressed with known PSI assembly factors as well as a phenotypic screening of promising candidates, the authors identified the factor CEPA1. A very comprehensive and thorough characterization of cepa1 mutant lines including complemented variants revealed that CEPA1 is specifically involved (but not strictly required) for proper accumulation of active PSI. Moreover, CEPA1 is shown to act on the posttranslational level. It localizes to stromal thylakoids and grana margin regions and co-migrates with PSI assembly intermediates in native gels. CoIP and BiFC assays revealed an interaction with PSA3 and most likely PPD1 two known PSI assembly factors. The clear findings lead the authors to a refined model for the PSI assembly process. In conclusion, this work provides new and interesting insights on the molecular organization of the highly coordinated biogenesis of PSI and adds a new factor to the growing list of associated eukaryotic assembly factors. Unfortunately, the molecular interrelationship to PSA3 could not be further dismantled due to the lack of an appropriate PSA3 antibody. This would have enabled the authors to dismantle the molecular working mode of PSA3 even further.

Regarding the phenotype of the cepa1 knockout mutant, the question arises whether it has been tested also under stress conditions (e.g., light and/or temperature) in order to challenge the residual PSI assembly activity?

In addition, the presentation of the enormous amount of data would benefit from some sorting/compacting as specified below.

- Figs. 1 and 4 could be fused because they address the initial characterization of cepa mutants.
- Figs. 2, 3, 5, 16 as well as Tab. 2 could be moved to the Supplemental section because they display mainly published data or control experiments.
- The description of Fig. 3 (lines 179 - 249) is overly detailed. In particular, the lengthy description of a putative CEPA1 homolog in Chlamydomonas is somewhat distracting.
- The experimental procedure is already well-described in the Methods section and does not need to be restated in the Results. Some of the statements in lines 153-154, 253-255, 260-262, 288-289, 294-296, 381-383, 400-403, 415-420, 427-429, 493-496, 419-421, 537-540 and 552-555 might be removed or shortened.

Reviewer #2 (Comments for the Author):

Rolo et al. report the discovery and functional characterization of CEPA1, a putative novel PSI assembly factor in Arabidopsis thaliana. This is an impressive work analyzing CEPA1 using a variety of methods. The work characterizes basic CEPA1 protein properties and thylakoid membrane localization. Overall, the data are sound and suggest a function of CEPA1 in PSI stability and possibly assembly. The data suggest that CEPA1 co-migrates with PSI higher molecular mass protein complexes and co-localizes with PSI in non-appressed thylakoid membranes. The only evidence of involvement in PSI assembly is protein-protein interaction with the PSI assembly factor PSA3. Despite the numerous efforts, the work remains descriptive and lacks mechanistic insights. Yet, the work identifies a new factor required for PSI accumulation.

Other comments.

1. An open question that remains is whether other PSI assembly factors are depleted in the absence of CEPA1. Mass spectrometric analyses should be conducted e.g. on leaves and/ or isolated chloroplasts comparing WT and CEPA1 deficient mutants.

2. The additional lower band in the PSAD immuno-blot in cepa1-3 is intriguing (Fig. 6). It could be a degradation product. Is this a reproducible observation? Could it be that CEPA1 is required for stabilizing PSAD? It is known that in the absence of PSAD, PSI is not stable. Does CEPA1 interact with PSAD? BiFC might be an option to test this interaction.

3. Is this proteolytic cleavage of PASD in the absence of CEPA1sufficient to destabilize PSI?

4. CEPA1 is co-localizing with PSI higher molecular mass protein complexes (Fig. 12). Is this localization indeed dependent on the presence of PSI? An alternative question is, whether CEPA1 is present in these higher molecular mass fractions in the absence of PSI (see also comment 5)?

5. Fig. 12, it is very difficult to judge with which protein complexes CEPA1 is co-migrating, as these are not all PSI containing complexes?

6. Fig. 13, proteolytic digestion is suggested, does degradation occur in the absence of PSI or in the absence of other PSI assembly factors, such as PSA3?

7. Minor. For the 10 LHCA in C. reinhardtii PSI better cite Ozawa et al 2018.

Reviewer #3 (Comments for the Author):

The manuscript "Co-expressed with PSI assembly 1 (CEPA1), a novel factor involved in photosystem I assembly in Arabidopsis" by Rolo et al. reports a novel factor involved in PSI assembly using a new approach to search for unknown PSI assembly factors. The authors searched for genes that are co-expressed with the five nucleus-encoded PSI assembly factors in Arabidopsis, finally identified CEPA1 as a PSI assembly factor, disrupted CEPA1 to find that a specific decrease in PSI accumulation and indicated that CEPA1 cooperates with PSA3. This gene is conserved in photosynthetic prokaryotes but is likely absent from cyanobacteria. Overall, the experiments are carefully designed, and the results obtained are clear and sound. Although it is usually difficult to provide evidence for PSI assembly factors, almost all possible experiments were performed in the present study. However, it is unfortunate that the interaction between CEPA1 and PSI subunits is not fully confirmed in the present study. It is clearly shown that CEPA1 and PSI proteins are in the same regions of thylakoid membranes, that is, grana margins and stroma lamellae. However, the direct interaction between CEPA1 and PSI proteins is suggested based on the co-migration by BN-PAGE. This result indicates that the sizes of CEPA1 in complexes are like those of PSI subcomplexes. Thus, it is necessary to show more direct evidence for the interaction. Pull-down experiments such as Co-IP can be good evidence for that, but unfortunately, Figure 16 shows no PSI proteins in FLAG co-IP fraction.
This data is important to discuss how CEPA1 is involved in the PSI assembly. It is likely that CEPA1 interacts with other assembly factors, such as PSA3, and assists or regulates PSI accumulation at wild-type levels. This reviewer expects the authors to identify components in the large complexes CEPA1 associates with.

Specific points:
Figure 2 shows the T-DNA insertion sites in cepa1-1, cepa1-2, and cepa1-3. Are there any deletions around the insertion sites? If yes, it is interesting to know the deletion regions, particularly in cepa1-1 and cepa1-2.
Figure 4C shows that CEPA1 is fragmented in older plants. Does this suggest that CEPA1 turns over fast? It is important to know this because Ycf3 and CGL71(PYG7) show rapid turnover.
Figure 8F: The authors suggested that the slight blue shift of the PSI-LHCI emission maximum is ascribed to small amounts of uncoupled LHCI. Figure 6 shows that most LHCIs except for LHC3 accumulated to the wild-type levels, although the amount of PSI was decreased in cepa1-2 and cepa1-3. These results indicate that substantial amounts of LHCI are disconnected from the PSI core complex.
Figure 9: Please confirm that the expression of the plastid-encoded Ycf3 and Ycf4 was not affected in cepa1-3.
Figures 12A and 15, and L433 and L546-547; The absence of PSI* may suggest that CEPA1 is involved in more initial steps of PSI assembly. The detection of CEPA1 as high molecular complexes is an interesting finding, and it is anticipated to know how CEPA1 is from large complexes and what interacting proteins are. Although several PSI complexes are separated in these size regions, it does not necessarily indicate that CEPA1 forms complexes with PSI subcomplexes (presumably newly synthesized PSI subcomplexes). It is necessary to show more direct evidence for the interaction between CEPA1 and PSI subcomplexes rather than solely discussing the sizes between CEPA1 and PSI subunits. The pull-down experiments in Figure 16 should detect some PSI proteins but not (L577-578). Thus, the evidence for the interaction between CEPA1 and PSI subunits is not sufficiently provided in the present study.
Figure 14: Indicate the positions of (a), (b), and (e) in the figure.

L656; The reference cited here (Kubota-Kawai et al. 2019) is not appropriate because ten LHCIs in Chlamydomonas PSI-LHCI were first reported by Ozawa et al. (Plant Physiology (2018) 178: 583-595) using biochemical methods and were then confirmed by analyzing the structure with cryo-EM by Suga et al. (Nature Plants (2019) 5: 626-636) and Su et al. (Nature Plants (2019) 5: 273-281)

We would like to thank the editor and the three reviewers for their thoughtful comments and the constructive feedback.

Below please find our point-by-point response to all comments. Reviewer comments are in black, our responses are in blue. In the marked-up copy of our revised manuscript, the textual changes are highlighted in yellow.

Editor:

In preparing your revision, please address all the specific points and questions raised by the reviewers. Streamline the presentation of the data as suggested, moving some to the supplemental section, and condensing detailed descriptions where appropriate. Finally, providing direct evidence to confirm the interaction between CEPA1 and PSI subunits would strengthen the work. However, if this is not possible, please discuss the limitation of the results of Figure 12.

As you will see from our point-by-point response below, we have (i) streamlined the presentation according to the reviewers suggestions, (ii) moved several display items to the supplements, as suggested, and (iii) added a substantial amount of new experimental data to address the reviewers' suggestions. Specifically, we have performed a set of lincomycin treatment experiments to address protein turnover (Supplemental Figure S5), performed chilling stress treatments to determine mutant phenotypes under conditions that are known to be challenging upon PSI deficiency (Supplemental Figure S6), and conducted BN-PAGE experiments with etiolated samples to determine CEPA1 association with HMW complexes in the absence of PSI biogenesis (Supplemental Figure S8).

The only point that remains unaddressed (and has been discussed accordingly, as suggested by the Editor) concerns the possible interaction of CEPA1 with PSI subunits. We tried extensively to co-purify CEPA1 with PSI subunits by conducting co-IP experiments coupled to mass spectrometry (IP-MS). Among the proteins enriched in the co-IP were two PSI assembly factors, namely PSA3 and PPD1, but no PSI subunit. Additional IP-MS experiments included the following technical modifications:

(i) We suspected that the beads could benefit from harsher washing steps (*e.g.*, using RIPA buffer) before elution to remove the non-specific background, although 6 washing steps were performed (as opposed to 3 as recommended by the beads manufacturer).

(ii) We applied crosslinkers to fix physical interactions between proteins, which is particularly relevant when working with potentially weak interactions, lowly abundant complexes and/or transient complexes, as it is likely the case in the context of PSI assembly. We tried crosslinking of the solubilized thylakoid membranes with 1 mM DSP or 1 mM DTSSP prior to IP, with no success.
(iii) We changed the bait: Instead of using anti-FLAG-coated beads, we attached anti-CEPA1 antibodies to protein G-coated beads, permanently fixed the anti-CEPA1-coated beads with the BS^3 crosslinking agent, and conducted the co-IP of native CEPA1. However, CEPA1 was not eluted, which may be due to the antibodies not recognizing CEPA1 after crosslinking.
As suggested by the Editor, we briefly discuss these attempts in our revised manuscript and the resulting limitation of our study that PSI subunits that could be clients of CEPA1 currently remain unknown (l.682-691).

Please note that, due to the requested change in figure arrangement (especially the merge of former Figure 1 and 4 into one figure, as suggested), several paragraphs needed to be moved accordingly. Since this did not result in textual changes, moved paragraphs are not highlighted in yellow in the marked-up manuscript version, to avoid confusion and retain readability of the text.

-- Reviewer comments:

Reviewer #1 (Comments for the Author):

The manuscript by Rolo et al. describes the identification and characterization of a new factor for the assembly of photosystem I in Arabidopsis thaliana. Based on a bioinformatical analysis of genes that are co-expressed with known PSI assembly factors as well as a phenotypic screening of promising candidates, the authors identified the factor CEPA1. A very comprehensive and thorough characterization of cepa1 mutant lines including complemented variants revealed that CEPA1 is specifically involved (but not strictly required) for proper accumulation of active PSI. Moreover, CEPA1 is shown to act on the posttranslational level. It localizes to stromal thylakoids and grana margin regions and co-migrates with PSI assembly intermediates in native gels. CoIP and BiFC assays revealed an interaction with

PSA3 and most likely PPD1 two known PSI assembly factors. The clear findings lead the authors to a refined model for the PSI assembly process. In conclusion, this work provides new and interesting insights on the molecular organization of the highly coordinated biogenesis of PSI and adds a new factor to the growing list of associated eukaryotic assembly factors. Unfortunately, the molecular interrelationship to PSA3 could not be further dismantled due to the lack of an appropriate PSA3 antibody. This would have enabled the authors to dismantle the molecular working mode of PSA3 even further.

We agree with the Reviewer that it will be interesting to further investigate the nature of the CEPA1-PSA3 relationship, to better understand the precise roles of CEPA1 and PSA3 in PSI assembly. Antibodies raised against PSA3 from *Zea mays* had previously been reported, but unfortunately, were shown to be functional in maize but not in *Arabidopsis* (Agrisera; Shen et al., 2017). We plan to invest in raising antibodies against the *Arabidopsis* PSA3 in the future to provide this tool.

Regarding the phenotype of the cepa1 knockout mutant, the question arises whether it has been tested also under stress conditions (e.g., light and/or temperature) in order to challenge the residual PSI assembly activity?

The *cepa1-3* knockout mutant shows a clear PSI deficiency (and mutant phenotype) already under standard growth conditions. To challenge the remaining PSI assembly activity, as suggested by the Reviewer, we performed chilling stress experiments (known to cause PSI photoinhibition; Lima-Melo *et al.*, 2021). Indeed, the phenotype of the knockout is strongly aggravated under these conditions, with the emerging leaf tissue (where PSI assembly is mainly occurring) being completely pale. Consistent with this severe phenotype, the PSI subunits PsaC and PSAD are below detectable levels in the cold-treated knockout.

These data are displayed in the new Supplemental Figure S6, a paragraph has been added to the Results (l.334-344), and experimental procedures have been added in the Materials and Methods (l.1,098-1,119).

In addition, the presentation of the enormous amount of data would benefit from some sorting/compacting as specified below.

- Figs. 1 and 4 could be fused because they address the initial characterization of cepa mutants.
- Figs. 2, 3, 5, 16 as well as Tab. 2 could be moved to the Supplemental section because they display mainly published data or control experiments.

As suggested by the reviewer, Figures 1 and 4 were merged. We chose to also include the former Figure 2B showing the gene map of *CEPA1* with its features and T-DNA insertion sites in the mutants, as it provides essential information for the reader to understand the mutant phenotypes. We have moved Figures 2 (re-assembled without panel 2B), 5 and 16, and Table 2 to the Supplemental data. We decided to keep Figure 3 (now Figure 2, displaying the alignment of putative CEPA1 homologs in photosynthetic eukaryotes) in the main manuscript, to properly illustrate the domain structure of the protein and the description of the (non-trivial!) homology searches.

As a consequence of the merged Figure, the paragraphs referring to the former Figure 4 had to be moved, as mentioned in the response to the Editor's comments.

- The description of Fig. 3 (lines 179 - 249) is overly detailed. In particular, the lengthy description of a putative CEPA1 homolog in Chlamydomonas is somewhat distracting.

We agree and have significantly condensed this section (l.251-256).

- The experimental procedure is already well-described in the Methods section and does not need to be restated in the Results. Some of the statements in lines 153-154, 253-255, 260-262, 288-289, 294-296, 381-383, 400-403, 415-420, 427-429, 493-496, 419-421, 537-540 and 552-555 might be removed or shortened.

Revised as suggested.

Reviewer #2 (Comments for the Author):

Rolo et al. report the discovery and functional characterization of CEPA1, a putative novel PSI assembly factor in Arabidopsis thaliana. This is an impressive work analyzing CEPA1 using a variety of methods. The work characterizes basic CEPA1 protein properties and thylakoid membrane localization. Overall, the data are sound and suggest a function of CEPA1 in PSI stability and possibly assembly. The data

suggest that CEPA1 co-migrates with PSI higher molecular mass protein complexes and co-localizes with PSI in non-appressed thylakoid membranes. The only evidence of involvement in PSI assembly is protein-protein interaction with the PSI assembly factor PSA3. Despite the numerous efforts, the work remains descriptive and lacks mechanistic insights. Yet, the work identifies a new factor required for PSI accumulation.

While we agree that the exact mode of action of CEPA1 in PSI assembly remains to be elucidated, we think that evidence of CEPA1 involvement in PSI assembly other than the CEPA1-PSA3 interaction is provided in our study, and is further supported by newly generated data.

We demonstrate that CEPA1 is involved in PSI accumulation specifically at the post-translational level. The accumulation of PSI subcomplexes, including the previously characterized PSI* intermediate (Wittenberg et al., 2017) are decreased in the absence of CEPA1. Additionally, CEPA1 co-migrates with complexes of high molecular weight which are likely to represent PSI subcomplexes, including PSI*, but not with the mature PSI-LHCI (which would not be expected, if CEPA1 would only play a role in PSI stability). Motivated by the reviewer's comments, we generated new data investigating the accumulation of PSI subunits in the *cepa1-3* knockout after lincomycin treatment. The data show that the absence of CEPA1 does not provoke an accelerated loss of PSI when *de novo* synthesis is blocked, suggesting that the fully assembled PSI is as stable in *cepa1-3* as in the wild type.

Moreover, CEPA1 associates with complexes containing not only one (PSA3), but at least a second (PPD1) PSI assembly factor. CEPA1 and PPD1 were co-purified not only in our co-IP assays with the CEPA1-FLAG, but also in co-IP assays with the *Chlamydomonas* PPD1 (Mackinder et al., 2017).

Other comments.

1. An open question that remains is whether other PSI assembly factors are depleted in the absence of CEPA1. Mass spectrometric analyses should be conducted e.g. on leaves and/ or isolated chloroplasts comparing WT and CEPA1 deficient mutants.

The fate of other PSI assembly factors in the absence of CEPA1 is an interesting question. As suggested by the Reviewer, we conducted mass spectrometric analyses of leaf proteins from *cepa1-3*, *cepa1-3-C1* and the wild type. While most

PSI assembly factors could not be reproducible detected across the four replicates conducted for each genotype, the assembly factor PSA3 was consistently detected. PSA3 was slightly upregulated in *cepa1-3* compared to the wild type and *cepa1-3-C1*, which may reflect a regulatory response to the CEPA1 deficiency. These results are briefly discussed in our revised manuscript (l.749-755).

2. The additional lower band in the PSAD immuno-blot in cepa1-3 is intriguing (Fig. 6). It could be a degradation product. Is this a reproducible observation? Could it be that CEPA1 is required for stabilizing PSAD? It is known that in the absence of PSAD, PSI is not stable. Does CEPA1 interact with PSAD? BiFC might be an option to test this interaction.

The presumptive degradation product of PSAD observed in *cepa1-3* is not always detectable. For instance, PSAD degradation is not observed in young *cepa1-3* seedlings grown on plates in more controlled growth conditions compared to growth on soil (now Figure 1C), but PSI accumulation is still reduced. To address the Reviewer's question, we investigated the fate of PSAD in *cepa1-3* treated with lincomycin. PSAD degradation after the treatment does not seem to be accelerated in *cepa1-3*. Therefore, we preliminarily conclude that the PSAD degradation intermediate may be accounted for by *cepa1-3* being more prone to photodamage, due to the general PSI deficiency.

We have added the new data as Supplemental Figure S5, added a statement to the Results section (l.270-282), and the experimental procedures were included in the Materials and Methods section (l.1,098-1,119).

Additionally, we tried to assess whether CEPA1 and PSAD directly interact in BiFC assays, but have not been able to detect an interaction.

3. Is this proteolytic cleavage of PASD in the absence of CEPA1sufficient to destabilize PSI?

As explained above, the lower band (potentially representing a PSAD degradation product) is not consistently detectable in the *cepa1-3* mutant. To address protein stability, *cepa1-3* and wild type seedlings were treated with lincomycin to block PSI biogenesis (by inhibiting plastid translation and, in this way, supply of the essential PSI subunits PsaA, PsaB and PsaC). The PSAD degradation product starts to accumulate in the wild type 4 days after the treatment, and this degradation does not

seem to be more pronounced in the absence of CEPA1 (Supplemental Figure S5). Even at timepoint T0, no PSAD degradation product accumulates in the 12-day-old *cepa1-3* seedling grown on plates. Therefore, it is more likely that the PSAD degradation that can be observed in the 4-week-old *cepa1-3* gown on soil is the result of the reduced PSI amounts in the mutant, rather than the cause.

In the 12-day-old *cepa1-3* seedlings, in which PSAD is not degraded (at T0; Supplemental Figure S5), PSAD and PsaC amounts (as proxy for PSI subunit accumulation) are lower than in the wild type. We, therefore, speculate that the presence of degraded PSAD can be explained by the generally lower amounts of PSI, which make the complex more susceptible to photooxidative degradation.

4. CEPA1 is co-localizing with PSI higher molecular mass protein complexes (Fig. 12). Is this localization indeed dependent on the presence of PSI? An alternative question is, whether CEPA1 is present in these higher molecular mass fractions in the absence of PSI (see also comment 5)?

In the absence of an *Arabidopsis* mutant that completely lacks PSI, we, alternatively, isolated membranes from etiolated *cepa1-3*, *cepa1-3-C1* and wild-type seedlings (in which no photosystem biogenesis occurs). Membranes were solubilized in 1% (w/v) β-DM and resolved in BN-PAGE to assess CEPA1 migration patterns by immunoblotting. In *cepa1-3-C1* small amounts of CEPA1-FLAG are anchored to the membrane, but only in the monomeric and low-molecular weight regions of the gel. Thus, in the absence of PSI (and active PSI assembly), CEPA1-FLAG does not associate with high-molecular weight complexes.

These new data are presented as Supplemental Figure S8, described in the Results (l.497-506), and the experimental details have been added to the Materials and Methods (l.1,098-1,119).

5. Fig. 12, it is very difficult to judge with which protein complexes CEPA1 is co-migrating, as these are not all PSI containing complexes?

The Reviewer correctly noticed that, while some of the complexes contain PSI subunits such as PSI* and complexes (d), (f), (g), no PsaA/PsaB was detected in complexes (a), (b), and (c). Such complexes could represent modules composed of PSI assembly factors. CEPA1 does not interact with itself according to our BiFC

assays (now Figure 13), and therefore, these complexes probably do not represent CEPA1 oligomers.

A statement was added to the Discussion to address this point (l.667-668)

6. Fig. 13, proteolytic digestion is suggested, does degradation occur in the absence of PSI or in the absence of other PSI assembly factors, such as PSA3?

Since we currently do not have an *Arabidopsis* mutant that completely lacks PSI or a *psa3* knockout mutant, we analyzed etiolated seedlings as alternative material that is devoid of PSI. CEPA1-FLAG is found in the monomeric fraction in BN-PAGE analysis of etiolated *cepa1-3-C1* samples, and the pattern in the low-molecular weight region of the gel is very similar in light-grown and etiolated seedlings (new Supplemental Figure S8). However, the one-dimensional BN gels cannot confidently resolve the two protein forms (2D separation has been attempted, but the low amount of material did not allow reliable detection).

The results from the analysis of CEPA1 in etiolated seedlings are displayed in the new Supplemental Figure S8, and briefly described in the Results section. In our revised manuscript, we also point out that analysis of CEPA1 in a PSA3-deficient mutant potentially can provide additional insight in the relationship between the two assembly factors (l.755-757).

7. Minor. For the 10 LHCA in C. reinhardtii PSI better cite Ozawa et al 2018.

The citation was replaced by Ozawa *et al.* (2018), as suggested (l.652).

Reviewer #3 (Comments for the Author):

The manuscript "Co-expressed with PSI assembly 1 (CEPA1), a novel factor involved in photosystem I assembly in Arabidopsis" by Rolo et al. reports a novel factor involved in PSI assembly using a new approach to search for unknown PSI assembly factors. The authors searched for genes that are co-expressed with the five nucleus-encoded PSI assembly factors in Arabidopsis, finally identified CEPA1 as a PSI assembly factor, disrupted CEPA1 to find that a specific decrease in PSI accumulation and indicated that CEPA1 cooperates with PSA3. This gene is conserved in photosynthetic prokaryotes but is likely absent from cyanobacteria. Overall, the experiments are carefully designed, and the results obtained are clear

and sound. Although it is usually difficult to provide evidence for PSI assembly factors, almost all possible experiments were performed in the present study. However, it is unfortunate that the interaction between CEPA1 and PSI subunits is not fully confirmed in the present study. It is clearly shown that CEPA1 and PSI proteins are in the same regions of thylakoid membranes, that is, grana margins and stroma lamellae. However, the direct interaction between CEPA1 and PSI proteins is suggested based on the co-migration by BN-PAGE. This result indicates that the sizes of CEPA1 in complexes are like those of PSI subcomplexes. Thus, it is necessary to show more direct evidence for the interaction. Pull-down experiments such as Co-IP can be good evidence for that, but unfortunately, Figure 16 shows no PSI proteins in FLAG co-IP fraction.

This data is important to discuss how CEPA1 is involved in the PSI assembly. It is likely that CEPA1 interacts with other assembly factors, such as PSA3, and assists or regulates PSI accumulation at wild-type levels. This reviewer expects the authors to identify components in the large complexes CEPA1 associates with.

Specific points:

Figure 2 shows the T-DNA insertion sites in cepa1-1, cepa1-2, and cepa1-3. Are there any deletions around the insertion sites? If yes, it is interesting to know the deletion regions, particularly in cepa1-1 and cepa1-2.

No deletion was observed at the T-DNA insertion site in any of the mutants. This was confirmed by sequencing the regions of interest in each mutant. A corresponding statement was added to the legend of the Supplemental Figure S2.

Figure 4C shows that CEPA1 is fragmented in older plants. Does this suggest that CEPA1 turns over fast? It is important to know this because Ycf3 and CGL71(PYG7) show rapid turnover.

This is a very good point. To estimate CEPA1 turnover, we have assessed the fate of CEPA1 when PSI assembly is blocked. To do so, wild type and *cepa1-3-C1* seedlings were treated with lincomycin (an inhibitor of plastid gene expression), and seedlings were harvested directly (T0), and after 2 and 4 days of treatment for thylakoid isolation and immunoblotting. CEPA1 accumulation (both full-length and fragmented) was strongly decreased already 2 days after the treatment, and was undetectably low 4 days after treatment in wild type. This suggests that, in the

absence of active PSI assembly, CEPA1 does not stably accumulate, presumably due to fast turnover.

These data are shown in the new Supplemental Figure S5, a description was added to the Results (l.491-496), and the experimental procedures were added to the Materials and Methods section (l.1,098-1,119).

Figure 8F: The authors suggested that the slight blue shift of the PSI-LHCI emission maximum is ascribed to small amounts of uncoupled LHCI. Figure 6 shows that most LHCIs except for LHC3 accumulated to the wild-type levels, although the amount of PSI was decreased in cepa1-2 and cepa1-3. These results indicate that substantial amounts of LHCI are disconnected from the PSI core complex.

We agree that “small” does not represent an accurate description and have rephrased the sentence, as suggested (l.329).

Figure 9: Please confirm that the expression of the plastid-encoded Ycf3 and Ycf4 was not affected in cepa1-3.

None of the plastid gene, including the genes encoding Ycf3 and Ycf4, is affected in *cepa1-3*, as revealed by our ribosome profiling (Figure 6, Supplemental Figure S7 and Supplemental Dataset S1). To highlight this observation, all PSI-related genes were labeled in Figure 6, and an explicit statement was added to the legend and the Results (l.363).

Figures 12A and 15, and L433 and L546-547; The absence of PSI* may suggest that CEPA1 is involved in more initial steps of PSI assembly. The detection of CEPA1 as high molecular complexes is an interesting finding, and it is anticipated to know how CEPA1 is from large complexes and what interacting proteins are. Although several PSI complexes are separated in these size regions, it does not necessarily indicate that CEPA1 forms complexes with PSI subcomplexes (presumably newly synthesized PSI subcomplexes). It is necessary to show more direct evidence for the interaction between CEPA1 and PSI subcomplexes rather than solely discussing the sizes between CEPA1 and PSI subunits. The pull-down experiments in Figure 16 should detect some PSI proteins but not (L577-578). Thus, the evidence for the interaction between CEPA1 and PSI subunits is not sufficiently provided in the present study.

Please see our response to the Editor’s comments. Briefly, despite extensive attempts and different sets of co-IP experiments (modifying a number of parameters, as described above), we have been unable to detect PSI subunits that would be specifically enriched in the tagged line. Possible reasons are now discussed in our revised manuscript, as explained in our comments to the Editor. We acknowledge that this is a limitation of our study, and will need to be addressed in future research (as now stated in our revised manuscript).

Figure 14: Indicate the positions of (a), (b), and (e) in the figure.
Done.

L656; The reference cited here (Kubota-Kawai et al. 2019) is not appropriate because ten LHCIs in Chlamydomonas PSI-LHCI were first reported by Ozawa et al. (Plant Physiology (2018) 178: 583-595) using biochemical methods and were then confirmed by analyzing the structure with cryo-EM by Suga et al. (Nature Plants (2019) 5: 626-636) and Su et al. (Nature Plants (2019) 5: 273-281)
The citation was replaced by Ozawa *et al.* (2018), as suggested (l.652).

Dear Ralph / Dr. Bock:

We are pleased to inform you that your paper entitled "CO-EXPRESSED WITH PSI ASSEMBLY 1 (CEPA1), a novel factor involved in photosystem I assembly in Arabidopsis" has been accepted for publication in The Plant Cell, pending a final editorial review by a science editor. At this stage, your manuscript will be evaluated by a science editor with respect to its presentation of scientific content, compliance with journal policies, and presentation for a broad readership. The Plant Cell has contracted with Plant Editors (planteditors.com) to provide this service to our authors, and you will soon receive additional information on this process.

Please note that each author needs to link their ORCID identifier to their account in the system before your manuscript can be published. If any authors do not have an ORCID linked to their account, they will receive a message with a link to complete this task. Please ensure that ALL of your coauthors have completed this task as soon as possible.

ASPB offers an OPEN option that allows authors to have their online articles available for free to all users immediately upon publication. For more information about the ASPB OPEN option, refer to the Final Submission Checklist Form.

The Plant Cell and The Arabidopsis Information Resource (TAIR) are collaborating to collect functional annotation data about Arabidopsis genes from authors. This includes information about the gene's molecular function (e.g., kinase activity, ATP synthetase activity), the biological process/es it is involved in (e.g., endosperm development, threonine biosynthesis), its subcellular location (e.g., nucleus, ER), anatomical or developmental expression pattern (e.g., leaf, ovule, flower stage 10, seedling stage), or its partner in a protein-protein interaction (e.g., AT1G01010 interacts with AT1G01020).

If your paper contains results falling into one or more of these categories for Arabidopsis genes, we request that you now submit these data for inclusion in TAIR by filling in the form provided at the following URL: https://www.arabidopsis.org/doc/submit/functional_annotation/123. If you need further clarification on what types of data can be submitted please contact curator@arabidopsis.org.

Finally, we encourage your submission of artwork for the journal cover. Monthly issues will have an online cover image and selected covers will be used for posters, other promotional items, and "wallpaper" for mobile devices. For more information, refer to Cover Submission in the Instructions for Authors https://tpc.msubmit.net/cgi-bin/main.plex?form_type=display_auth_instructions.

We look forward to seeing your paper published.

On behalf of the editorial board,

Roberta Croce, Board of Reviewing Editors
Nancy Eckardt, Senior Editor
Blake Meyers, Editor-in-Chief

The Plant Cell

=====
IMPORTANT REMINDER: PEER REVIEW REPORTS
=====

If you opted to publish a peer review report along with your article during the original submission process, it will be prepared by the editorial staff and publicly posted with your manuscript, inside the zip file that contains any other supplemental material. As a reminder, the peer review report is a public record of all comments from editors and reviewers, as well as your prior responses, as you received them in the decision letters for each draft of your manuscript. If you agreed to publish this report and have changed your mind, or are not sure if you selected this option, please contact the editorial office as soon as possible before signing the license agreement from our publisher.

----FOR ASPB OFFICE USE ONLY (DO NOT EDIT)----
MSID: 36202
Corresponding Author: Prof. Dr. Bock
